# Science‐Towards‐Technology Breakthrough in CO_2_ Electroreduction: Multiphysics, Multiscale, and Artificial Intelligence Insights

**DOI:** 10.1002/adma.202516978

**Published:** 2026-03-20

**Authors:** Ping Hong, Changfan Xu, Huaping Zhao, Yong Lei

**Affiliations:** ^1^ School of Environmental & Chemical Engineering Jiangsu University of Science and Technology Zhenjiang Jiangsu P. R. China; ^2^ Fachgebiet Angewandte Nanophysik Institut für Physik & IMN MacroNano Technische Universität Ilmenau Ilmenau Germany

**Keywords:** CO_2_ electroreduction, eCO_2_RR, multiscale regulation, synergistic effect

## Abstract

Electrochemical carbon dioxide reduction reactions (eCO_2_RR) are a key technology for converting greenhouse gas CO_2_ into high‐value‐added chemicals. In recent years, significant progress has been made in material design, catalytic mechanism analysis, and electrolyzer optimization. However, there remains a gap between “laboratory science” and “engineering practice” in current research. Most reviews are primarily based on the “material‐structure‐performance” model and have not yet established an integrated technical landscape combining multi‐physics, multi‐scale, and artificial intelligence (AI). This review centers on the industrialization goals of eCO_2_RR, establishing a multi‐scale research framework spanning from fundamental mechanisms to systems engineering. It covers four core areas: atomic‐level mechanism interpretation and characterization, interface microenvironment regulation, external field‐assisted optimization, and AI‐driven material design and reaction prediction. Through the closed‐loop integration of mechanism‐characterization‐optimization, this review emphasizes an overall synergistic strategy from materials to devices and from experiments to systems, aiming to establish a systematic research pathway for eCO_2_RR. This work not only provides a comprehensive research blueprint for the eCO_2_RR field but also offers methodological and strategic references for AI‐enabled catalytic material development, external field‐coupled performance enhancement, and the engineering of electrochemical carbon resource conversion.

## Graphical abstract

Electrochemical CO_2_RR is a key technology for converting CO_2_ into chemicals, but there remains a gap between “laboratory science” and “engineering practice” in current research. This review establishes a multi‐scale research framework, encompassing atomic‐level characterization, microenvironment regulation, external field‐assisted optimization, and AI‐driven material design and prediction. This provides a comprehensive roadmap and research guidance for bridging fundamental understanding to industrial applications.

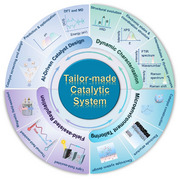

## 1 Introduction

Amid the profound transformation of the global energy structure and the gradual implementation of carbon neutrality commitments, the resource utilization of carbon dioxide (CO_2_) has transcended the scope of mere environmental governance and has become an important pillar in addressing climate change and building a green chemical industry system [1, 2, 3]. Among these, electrochemical CO_2_ reduction reactions (eCO_2_RR) have demonstrated unique advantages among numerous carbon conversion pathways due to their ability to convert CO_2_ into high‐value‐added carbon‐based products under mild conditions and their potential for high integration with renewable power systems (such as solar and wind energy) [4, 5]. In recent years, at the laboratory level, through the rational design of catalysts, electrodes, and electrolyte systems, eCO_2_RR has been able to selectively produce a series of useful chemicals and fuels, including single‐carbon products (such as CO, HCOOH, CH_4_) and multi‐carbon products (such as C_2_H_4_, C_2_H_5_OH, C_3_H_7_OH, etc.), thereby providing the possibility of constructing a closed‐loop chemical supply chain with CO_2_ as a carbon source [6, 7, 8, 9, 10]. Over the past decade, eCO_2_RR has made breakthroughs in catalyst development, electrolyzer engineering, interface control, and mechanism investigation [11, 12, 13, 14, 15]. Various novel metal single atoms [16, 17], alloys [18, 19], metal‐nonmetal synergistic systems [20, 21], two‐dimensional (2D) materials [22, 23], and defect‐controlled systems [24, 25] have been constructed to optimize reaction pathways and enhance multi‐electron transfer efficiency. In the field of reaction engineering, the introduction of gas diffusion electrodes (GDEs), flow cells, and membrane electrode assembly (MEA) electrolyzers has effectively alleviated the limitations imposed by the low solubility of CO_2_, significantly enhancing reaction current density and extending operational lifespan [26, 27, 28]. More importantly, rapid advances in theoretical simulation and in situ characterization have enabled the analysis of reaction intermediates, electronic structure responses, and interfacial processes at the atomic level, providing a solid foundation for establishing structure–property causal models [29, 30, 31, 32].

**FIGURE 1:**
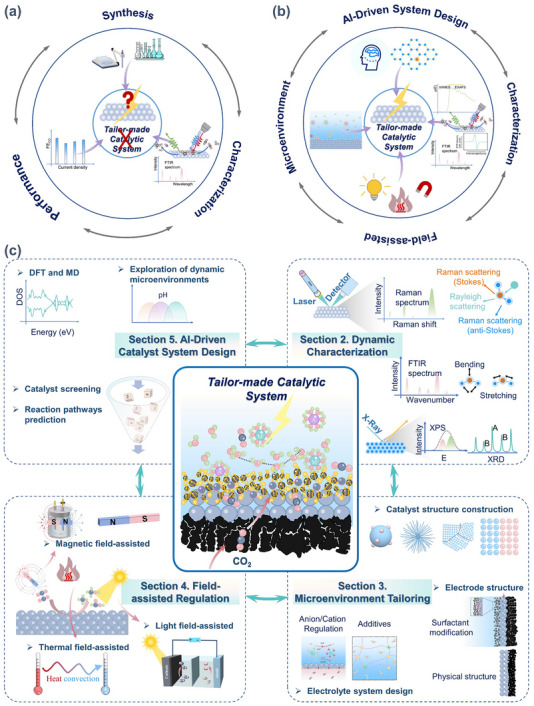
(a) Schematic illustration of the existing “synthesis‐performance‐characterization” model and (b) “multiscale”, “multi‐physics,” and “artificial intelligence” assisted investigation model. (c) A multiscale review framework of science to technology in this review, covering atomic‐scale mechanistic insights via in situ/operando characterization, microenvironment modulation, external field‐coupled optimization, and AI‐driven prediction.

However, despite continuous improvements in performance metrics at the laboratory level, CO_2_RR still faces fundamental obstacles to engineering feasibility. First, eCO_2_RR is a multi‐step, highly competitive electrocatalytic process whose efficiency and selectivity depend not only on the catalyst itself, but also on multiple factors such as electrode structure, electrolyte composition, interfacial microenvironment, and external physical fields, thereby constituting a complex, multivariable, nonlinear system [33, 34, 35]. Existing research has mostly focused on optimizing isolated variables, lacking sufficient exploration of the interactive patterns between key variables within the system, resulting in poor generalizability of research findings across different systems. Secondly, the vast majority of structure–property correlation studies are still based on static structural models or ex situ characterization (Figure 1a). However, in real reaction processes, active centers are often in a metastable or dynamic state, and their charge distribution, coordination environment, and even physical phase may change significantly with the reaction process and local environment. These changes have a decisive influence on the reaction pathway selection and product distribution. Furthermore, experimental designs are often based on idealized test platforms (such as H‐type electrolytic cells and CO_2_‐saturated electrolytes), which are significantly disconnected from real industrial environments (high current density, gas‐liquid interface reactions, and stability requirements). A more fundamental challenge lies in the fact that current mainstream research remains rooted in an “experience‐driven + single‐variable optimization” linear approach. This approach has increasingly revealed its inefficiencies, high costs, and lack of reproducibility. When applied to the complex, multi‐scale, multi‐physics, and multi‐variable eCO_2_RR systems, both research efficiency and industrial scalability face significant bottlenecks. Therefore, addressing these challenges requires expanding the traditional “material‐structure‐performance” model to a research framework centered on scientific mechanisms, with cross‐scale modelling as the framework, information science as the accelerator, and systems engineering as the basis for verification. This shift gives rise to the following new perspectives and breakthroughs (Figure 1b):

- At the microscopic level (dynamic catalytic mechanisms at the atomic/molecular scale), current catalyst design primarily relies on static characterization and ex situ material analysis. However, the dynamic evolution of catalyst surface morphology, crystalline phase composition, and active sites during catalytic processes often determines product pathways and selectivity, and is also the foundation for understanding complex reaction mechanisms [36, 37, 38]. There is an urgent need to develop or design in situ/operando characterization techniques with spatiotemporal resolution to capture the structural evolution of catalysts, the dynamics of interface intermediates, and the mechanisms of local environment modulation, thereby revealing the intrinsic connection between reaction pathways and structural regulation. ii) At the mesoscale, CO_2_RR is a typical gas–liquid–solid three‐phase interface reaction process. The selectivity and reaction kinetics of CO_2_RR are influenced by the microenvironment at the interface, including pH, electric field strength, ion concentration distribution, and local CO_2_ concentration [39, 40, 41]. By constructing an interface structure with spatial gradients, chemical heterogeneity, and coordinated hydrophilicity and hydrophobicity, it is possible to synergistically regulate the reaction barrier and the stability of intermediates, thereby providing a potential means of improving product yield. iii) At the macro level, CO_2_RR is a typical multi‐physics field synergistic process. In addition to the electric field, external physical fields such as thermal, optical, and magnetic fields can dynamically regulate key factors at the reaction interface, including electron density distribution, intermediate adsorption strength, and local reaction environment, without altering the intrinsic structure of the catalyst. This opens up new avenues beyond traditional electrocatalysis. Such multiple physical fields often exhibit nonlinear synergistic effects, providing higher‐dimensional control means for optimizing reaction system performance. For example, photoelectrochemical coupling can enhance CO_2_ adsorption and intermediate activation, magnetic field control can induce spin‐selective reaction pathways, and temperature fields can significantly influence the coupling process of polycarbonate products [42, 43, 44]. However, the current understanding of multi‐physics field regulation mechanisms remains at an empirical stage, lacking predictable and designable quantitative models, as well as systematic modelling of the synergistic relationships between catalyst structures and reaction pathways. iv) At the same time, the introduction of artificial intelligence (AI) is profoundly changing the research logic of electrocatalytic reactions. AI can integrate data resources from experiments, theoretical simulations, and high‐throughput screening to reveal nonlinear relationships between structure, performance, and environment, enabling high‐throughput screening, performance prediction, and reverse design [45, 46, 47]. When combined with traditional physical modelling methods such as density functional theory (DFT) or molecular dynamics simulations (MD), AI can construct hybrid models with causal logic and predictive capabilities, enabling information connectivity across different scales and improving modelling accuracy. Furthermore, AI has demonstrated unique value in control system optimization, reaction parameter scheduling, and intelligent operation of electrolyzers, offering the potential to fundamentally enhance the response efficiency and industrial scalability of CO_2_RR systems.

Based on the above considerations, the future development of CO_2_RR technology should not be limited to optimization at a single scale or stage, but should instead adopt a system engineering approach to promote the organic integration of atomic‐scale mechanism understanding, nano‐interface environment control, reaction field synergy enhancement, AI algorithm‐driven design, and macro‐scale system integration. Although there have been several reviews on eCO_2_RR catalysts, electrolytes, electrode engineering, or reactor design, these studies have primarily focused on single scales or material dimensions, lacking cross‐scale integration from atomic mechanism analysis, multi‐physics field regulation, and AI‐enabled pathways to system integration [48, 49, 50, 51, 52, 53]. Therefore, this review attempts to organize cutting‐edge research around three main structural axes, i.e., “multiscale”, “multi‐physics”, and “artificial intelligence”, to summarize key advances in CO_2_RR research, from microscopic mechanism exploration to macroscopic system construction. Specifically, this review is organized into the following core sections (Figure 1c): (1) Atomic‐level catalytic mechanism analysis via in situ/operando characterization methods; (2) Dynamic regulation of nano‐ and mesoscale interface environments; (3) Control strategies under multi‐physics field coupling; (4) Application of AI in catalyst design and reaction prediction; (5) Outlook for industrialization. This review will systematically discuss the above five core components, aiming to provide a theoretical framework, methodological tools, and future directional guidance for deepening the scientific understanding and engineering transformation of eCO_2_RR technology. It will also offer strategic guidance for constructing efficient, stable, and scalable CO_2_ resource utilization platforms, and explore new application frontiers for the integration of AI and multi‐physics field‐based catalytic research.

## 2 Revealing Catalytic Mechanisms at Atomic/Molecular Levels

Understanding the key to eCO_2_RR at the atomic/molecular scale lies in clarifying how the initial structure of a catalyst evolves into a “dynamic reaction interface” with unique electronic states and adsorption properties under typical reaction potentials, which governs reaction pathways and product selectivity [54, 55, 56, 57]. Hence, mechanism insights need not only to identify the initial active centers, but also to capture their structural responses during the reaction, the pathways for forming key intermediates, and potential failure modes. This chapter is organized around two complementary aspects: (i) the complete lifecycle of catalysts, from intrinsic actions through dynamic evolution to eventual failure; and (ii) how in situ and quasi‐in‐situ techniques redefine our perceptions of these processes.

### 2.1 Mechanism of Catalyst Action and Deactivation Process

The function of CO_2_RR catalysts can be summarized as lowering the energy barrier of key steps, stabilizing target intermediates, and selectively guiding product distribution [58, 59, 60, 61, 62]. This process typically involves the adsorption and activation of CO_2_ molecules, the formation of key intermediates, the synergistic transfer of proton‐electron pairs, and the branching of product pathways. Therefore, a comprehensive understanding of the mechanism of CO_2_RR catalysts requires an analysis at three levels: (i) how the catalyst activates CO_2_ at the molecular level and guides product branching (intrinsic function); (ii) structure reconstruction and dynamic evolution driven by reaction conditions; and (iii) how evolution leads to irreversible deactivation (the essence of failure) along specific thermodynamic and kinetic pathways.

#### 2.1.1 The Intrinsic Role of Catalytic Function

CO_2_ molecules have a linear configuration and stable C═O bonds, which result in a high energy barrier for direct electron transfer processes [63, 64]. The intrinsic function of catalysts is to regulate electron density, adsorption models, and interfacial electric fields to achieve stepwise CO_2_ activation and selective conversion.

**FIGURE 2:**
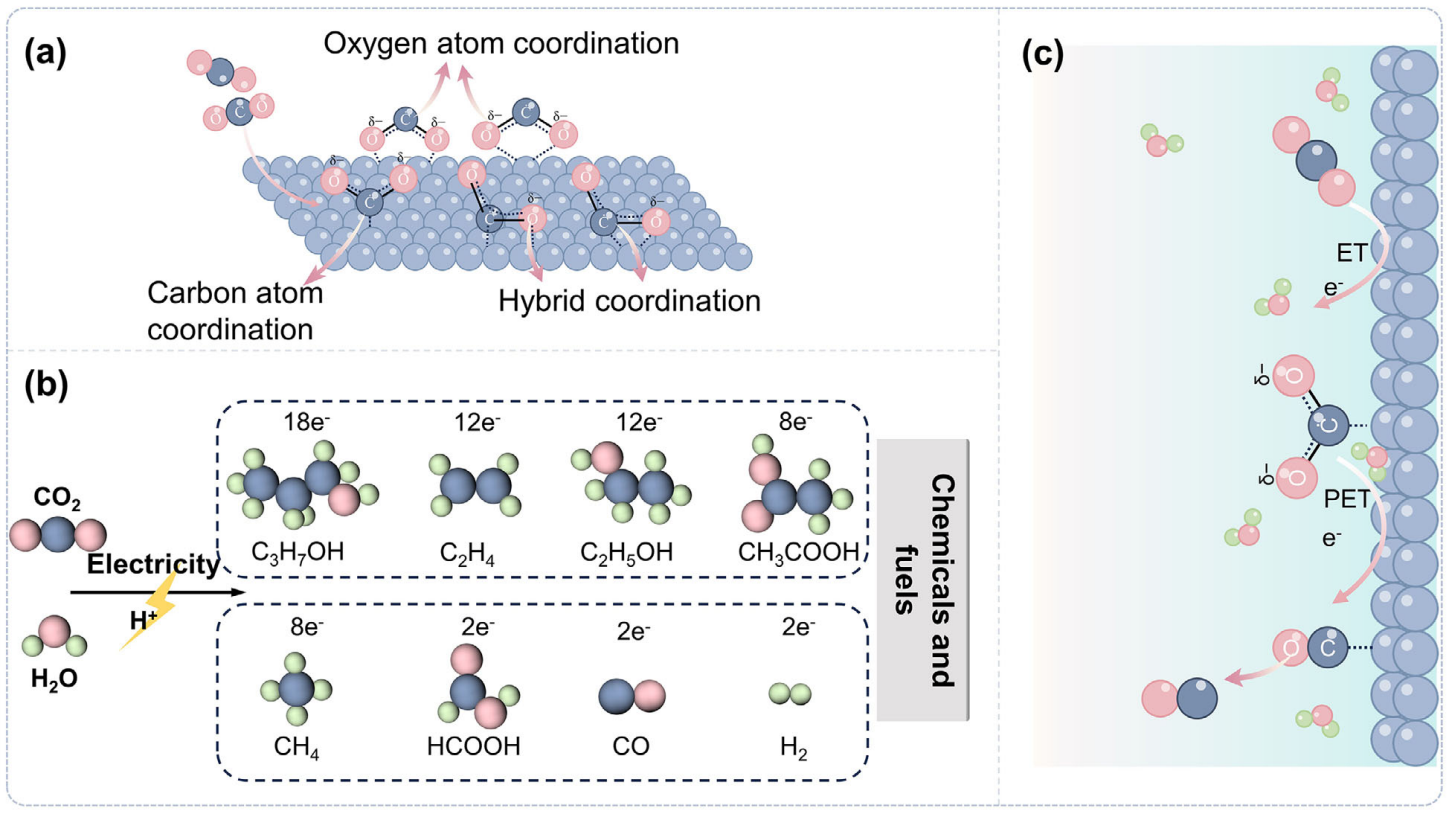
(a) Different coordination modes of CO_2_ molecules at catalytic sites during the initial activation step; (b) Products from the reaction pathway branching; (c) Schematic of the CPET process (using CO as an example).

1. Electron excitation and stabilization of the initial activation step (*CO_2_^−^ generation). In eCO_2_RR, the first step is to activate the inert CO_2_ molecules. CO_2_ molecules are fixed at catalytic sites through bonding and acquire electrons, forming partially negatively charged intermediates (denoted as *CO_2_^−^ or polarized CO_2_). Efficient activation depends on two factors [65, 66, 67]: (i) they must provide sufficient electron density to effectively inject electrons into the lowest unoccupied molecular orbital (LUMO) of CO_2_. (ii) after electron injection, the bent or polarized intermediates must be stabilized in an appropriate bonding mode to prevent rapid electron rebound or premature protonation of the intermediates. Additionally, atoms on the catalytic site surface can interact with CO_2_ through different coordination modes (Figure 2a) [68, 69]: O‐terminated sites binding with Lewis acid sites enhance molecular polarization; C‐terminated sites interacting with Lewis base sites induce electron transfer, forming carbonate‐like structures. In some systems, C‐ and O‐termini may simultaneously participate in bonding, forming mixed coordination [68, 70]. The resulting initial activated state not only determines the accessibility of the subsequent reaction coordinate but also often becomes a kinetic bottleneck.
2. Reaction pathway branching control. After CO_2_ is successfully activated, the direction of the reaction is determined by the formation and stability of key intermediates. Typically, branching points include (Figure 2b): (i) The formation of *COOH and *OCHO determines whether the reaction proceeds along the CO or HCOOH (formic acid) pathway [71, 72, 73]. (ii) The further conversion of *CO triggers a second branching point, leading to the hydrogenation of C_1_ products (e.g., formaldehyde, methanol, methane) [74, 75, 76, 77] or the formation of C_2+_ products via C─C coupling [78, 79, 80]. The adsorption strength of the catalyst on intermediates can modulate selectivity: weak adsorption may lead to premature desorption of intermediates, while strong adsorption may deactivate active sites or hinder desorption, which will influence subsequent reactions [81, 82, 83]. Therefore, product selectivity is essentially an energy gmatching issue between the adsorption energy of intermediates and the desorption/coupling kinetics. Specifically, metals (Bi, Sn, In, Pb, etc.) selective for *COOH primarily generate formate or HCOOH [84, 85, 86, 87]; metals (Au, Ag, etc.) with weak CO adsorption favor CO generation [88, 89]; catalysts with strong CO adsorption (primarily Cu and its oxides) that can promote C−C coupling of adsorbed *CO and proton/electron transfer to generate C_2+_ products [90, 91].
3. Concerted proton–electron transfer regulation (CPET). CPET is another factor influencing pathway selection (Figure 2c). In electrocatalysis, the sources of protons (H_2_O or H_3_O^+^) and electrons are not synchronized, and concerted transfer is required to avoid the accumulation of high‐energy barrier hydrogenation states [92, 93, 94]. Both experimental and theoretical studies have shown that regulating the structure of interfacial water or the composition of the electrolyte can significantly alter the rate matching of CPET, thereby influencing the C_1_/C_2_ ratio [95, 96]. Highly efficient CO_2_RR catalysts need to balance multiple energy barriers in activation, diversion, and synergistic transfer, but these high‐activity features may also trigger structural instability or enhanced side reactions, laying the ground for subsequent failure.

#### 2.1.2 Catalyst Structural Reconstruction and Dynamic Evolution

A growing field of cutting‐edge studies continues to confirm that catalysts form a “true active phase” under operating conditions, which is completely different from their initial state [97, 98]. These transformations primarily stem from three driving forces: electrochemical, interfacial chemical, and physical‐field driving forces.

1. Electrochemical‐driven. Primarily manifested in changes in valent states and phases [99]. A typical example is the transformation of transition metal oxides into metallic states at reducing potentials, resulting in metastable phases rich in grain boundaries or low‐coordination sites [100]. Such metastable states often significantly enhance the stability of certain intermediates (e.g., *CO) over short timescales (seconds to minutes), favoring C−C coupling reactions, but finally collapse to thermodynamically stable states [101]. Valence state changes also affect local electron density distributions, thereby altering the adsorption energies of intermediates [102, 103, 104].
2. Interface chemistry‐driven. Coordination/doping from reaction intermediates or electrolyte molecules [105]. For example, single‐atom sites (M–N–C type) may undergo partial coordination dissociation under the combined action of an electric field and solvent, forming low‐coordination active centers or small clusters, which can temporarily boost catalytic activity. Concurrently, selective enrichment of anions/cations at the interface alters the double layer and solvation shell, inducing redistribution of local pH and electric field gradients, thereby driving structural self‐reorganization [106].
3. Physically driven (including bubble impact, localized Joule heating, and mechanical/thermal stresses induced by electric field gradients, etc.). These external mechanical events can promote particle migration, interface peeling, or preferential crystal plane reconstruction at the nanoscale. The formation and desorption of bubbles particularly cause instantaneous mechanical shear, which drives particle migration and agglomeration over time, reducing specific surface area [107].

In fact, these dynamic evolutionary processes exhibit duality. On short timescales, evolution may generate highly active metastable sites; for instance, the “start‐up activity” could be observed in many catalytic systems [108]. However, on longer timescales, if these metastable states cannot be locked or periodically regenerated through interface design or operational strategies, the system will irreversibly progress toward agglomeration, dissolution, or coverage, thereby losing its long‐term performance [97].

#### 2.1.3 Fundamental Attribution of Catalyst Deactivation Mechanisms

**FIGURE 3:**
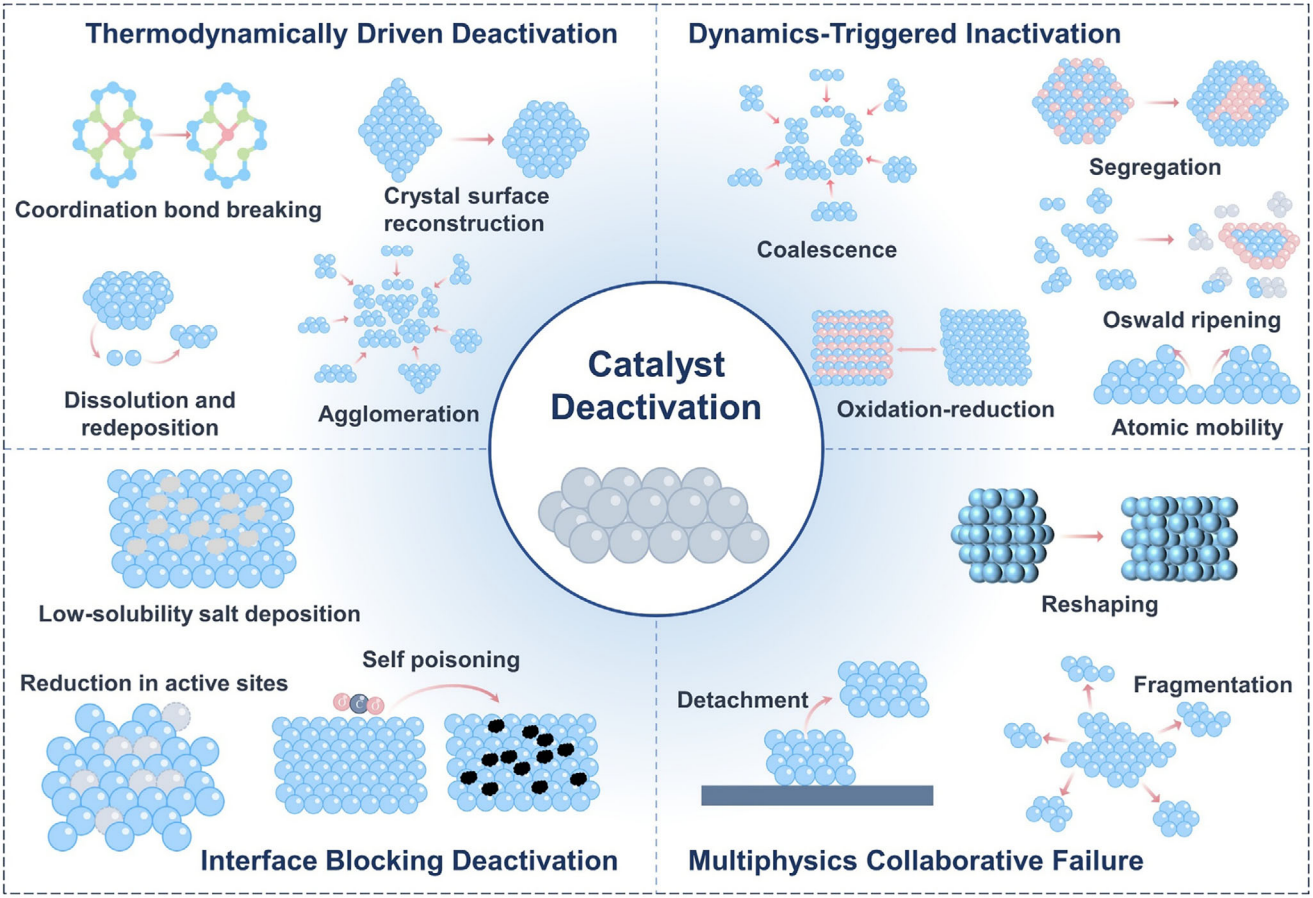
Schematic diagram of catalyst deactivation.

The failure of electrocatalytic systems is essentially a multiscale, cascade‐amplified chain reaction triggered by multiple driving factors. Here, these are summarized into four core mechanisms: thermodynamic‐driven failure, kinetic‐triggered failure, interfacial blockage failure, and multi‐physics field‐synergistic failure (Figure 3).

1. Thermodynamic‐driven failure. High‐energy structures (i.e., single atoms, metastable phases) tend to spontaneously evolve toward states with reduced free energy [108, 109]. Under electrochemical conditions, coordination bond breakage or metal dissolution may occur (e.g., Fe or Ni dissolution at high negative potentials or pH fluctuations), forming metal ions in solution or cluster precipitates [110, 111, 112]. Once this occurs, active sites are irreversibly lost, and the local interfacial charge and solvation environment are altered, which in turn increases the degradation probability of neighboring sites.
2. Kinetic‐triggered failure. Certain intermediates (especially strongly adsorbed multi‐carbon species, such as *CO or *OCCO) may alter the local atomic bonding environment [113], leading to a reduction in the migration energy barrier of surface atoms and triggering atomic rearrangement and local agglomeration. These processes exhibit autocatalytic behavior: larger particles formed are more easily adsorbed and promote the dissolution of surrounding smaller particles (Osterwald maturation), resulting in a nonlinear increase in deactivation rates over time and potentially triggering phase separation [108]. It becomes particularly pronounced under high current densities or high intermediate coverage conditions.
3. Interface blockage failure. In neutral or weakly alkaline electrolytes, carbonate/bicarbonate ions in the solution form low‐solubility salts with metal cations, which preferentially precipitate at the three‐phase interface, blocking the mass transfer pathways between gas, liquid, and solid phases [114]. It will decrease CO_2_ supply, leading to a shift in product distribution toward solvent‐accessible short‐range reactions (such as the hydrogen evolution reaction, HER), while exacerbating local pH and electric field inhomogeneities, promoting the formation of metal hydroxides that cover the catalytic surface, and further deteriorating CO_2_RR activity.
4. Multi‐physics field synergistic failure. Under industrial conditions with high current densities, the combined effects of bubble‐induced mechanical impact (providing migration/detachment momentum), electric field gradient‐induced stress (influencing ion distribution and electron‐injection pathways), and localized Joule heating (lowering migration energy barriers) collectively drive nanoparticle migration, crystal plane reconstruction, and even sintering. Such synergistic failure often manifests at industrial scaling and represents a critical engineering challenge for achieving long‐term stable operation.

These failure mechanisms are causally coupled across temporal and spatial scales: short‐timescale microscopic behaviors such as coordination bond breaking and redeposition may alter current/potential distribution within hours; whereas agglomeration and phase transitions occurring over hours to hundreds of hours manifest macroscopically as sustained decays in activity and selectivity. Therefore, assessing catalyst lifetime requires establishing a unified evolutionary process at the atomic, interfacial, and device scales, rather than basing it solely on a single “deactivation half‐life”. With this point, in situ and quasi‐in‐situ characterization techniques are not merely tools for validating the above mechanisms but are essential for redefining the true active centers and reaction pathways in eCO_2_RR.

### 2.2 The Role of In Situ/Quasi‐In‐Situ Characterization in eCO_2_RR

**FIGURE 4:**
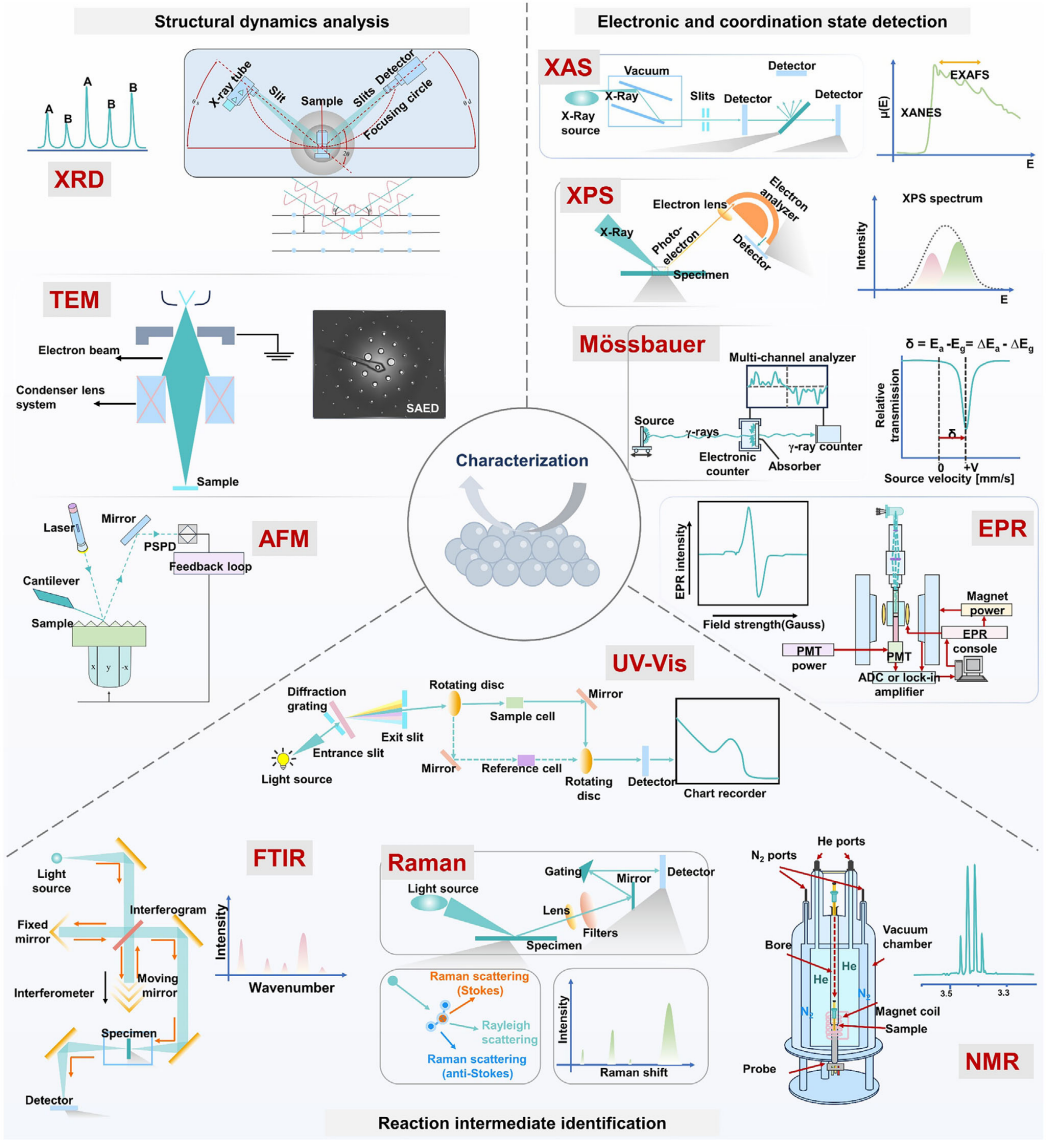
A summary of representative in situ/quasi‐in‐situ techniques, including apparent structural dynamics analysis (XRD, TEM, AFM), electronic and coordination state detection (XAS, XPS, Mössbauer, EPR), and reaction intermediate identification (UV–Vis, FTIR, Raman, NMR).

In Section 2.1, we introduced a dynamic reaction framework from CO_2_ activation pathways, adsorption intermediate distribution mechanisms, to failure kinetics. However, these insights depend on the real‐time capture and analysis of the catalyst structure, electronic states, coordination environment, and interfacial reaction intermediates at working potentials. These characteristics may form and disappear within milliseconds to seconds, directly determining reaction pathways and product distributions. Therefore, the core challenge in understanding the eCO_2_RR mechanism lies in whether it is possible to simultaneously capture and analyze these processes under real operating conditions, and correlate causally with reaction selectivity. Traditional ex situ characterization can only provide “static snapshots” before and after the reaction and cannot resolve transient active species during operation, which may lead to errors in mechanism interpretation. In recent years, in situ and quasi‐in‐situ characterization techniques have emerged as essential tools for understanding these challenges. This section will focus on three key aspects (structural dynamics, electronic state regulation, and intermediate identification) to discuss the relevant techniques and their complementarities (Figure 4).

#### 2.2.1 Visualization of Structural Evolution

**FIGURE 5:**
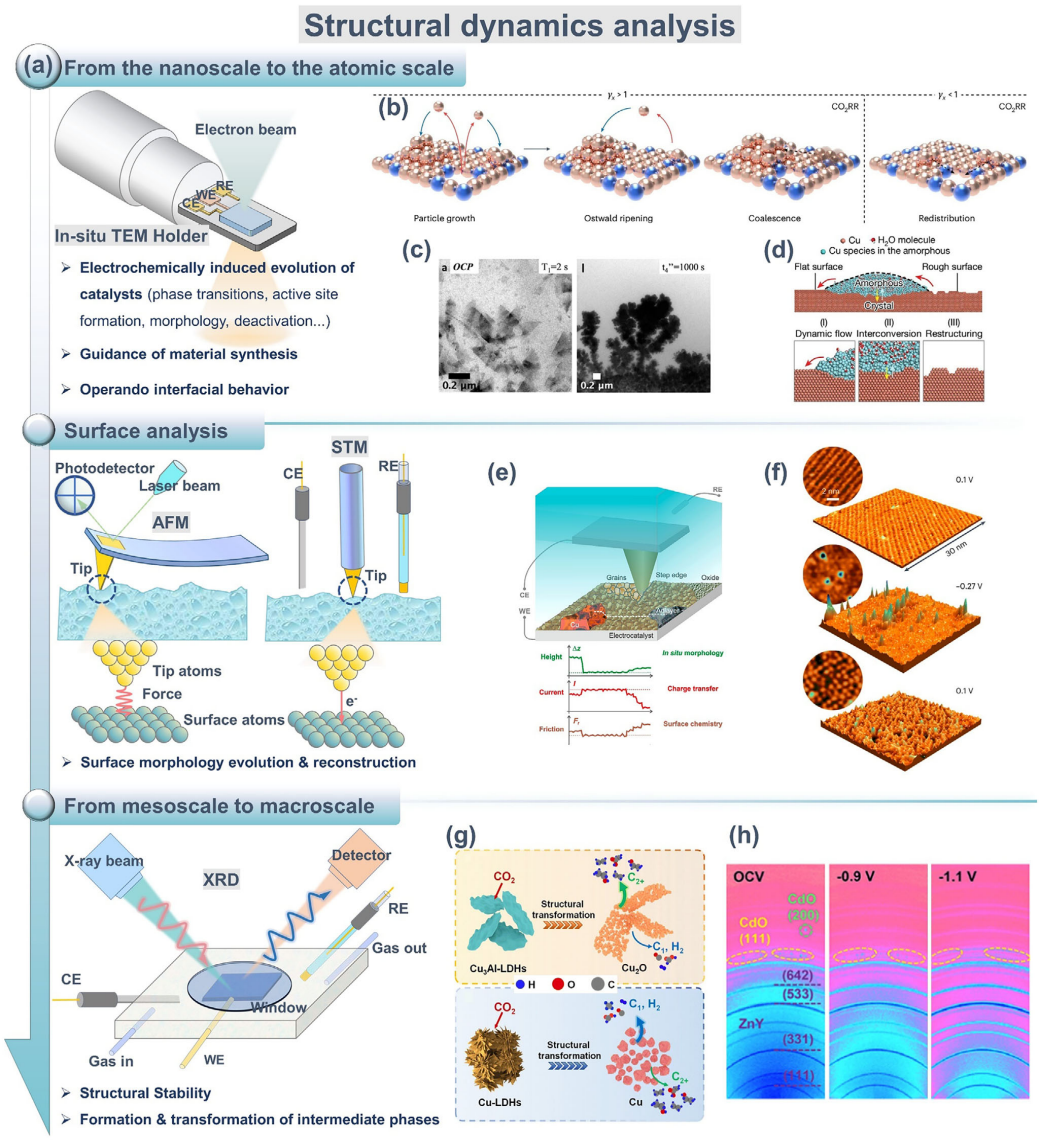
(a) TEM, AFM, STM, and XRD for structural dynamic analysis (CE, counter electrode; RE, reference electrode; WE, working electrode). (b) Reconstruction mechanism of the Cu–X catalyst. (Black, red, brown, and blue spheres denote C, O, Cu, and X, respectively. Red and blue arrows indicate dissolution and redeposition.) Reproduced with permission [117]. Copyright 2025, The Author(s). (c) Catalyst structure at OCP and after 1000 s under operating conditions. Reproduced with permission [118]. Copyright 2021, The Author(s). (d) Schematic showing the reconstruction of the crystal Cu surface mediated by the amorphous phase. Reproduced with permission [120]. Copyright 2024, The Author(s). (e) Illustration of an in situ c‐AFM device. Reproduced with permission [124]. Copyright 2023, The Authors. (f) In situ STM images of Cu(100). Reproduced with permission [126]. Copyright 2023, The Author(s). (g) Schematic reconstructions of Cu_3_Al‐LDHs and Cu‐LDHs. Reproduced with permission [127]. Copyright 2025, The Royal Society of Chemistry. (h) In situ 2D‐XRD patterns of CdO@ZnY. Reproduced with permission [128]. Copyright 2024, American Chemical Society.

The transient structure of catalysts (including morphology, crystal phase, surface reconstruction, grain boundaries, and defect density) continuously evolves under operating conditions and directly influences the formation and deactivation pathways of active sites. Therefore, building a structural evolution map of catalysts under realistic conditions is a prerequisite for understanding reaction mechanisms and designing stable electrocatalysts. Structural visualization usually relies on three complementary in situ/quasi‐in‐situ techniques (Figure 5a): direct imaging techniques (e.g., in situ transmission electron microscopy, TEM) can capture transient events like single‐atom migration, formation and flow of amorphous layers, and lattice rearrangement at the nanometer to atomic scale, revealing the generation, destruction, and regeneration of active states; surface probe techniques (like in situ atomic force microscopy (AFM) and scanning tunneling microscopy (STM)) can resolve atomic‐level reconstruction of steps, edges, and adsorption layers, as well as the response of local density of states to structural fluctuations; diffraction analysis methods (such as in situ X‐ray diffraction, XRD) provide statistical information on crystal orientation, strain evolution, and phase stability, linking local structural disturbs to a collective behavior of the overall catalyst system. These three techniques bridge atomic, interfacial, and crystal levels to construct a cross‐scale dynamic structure map, offering structural constraints and physical boundary conditions for subsequent electronic state and intermediate analysis (Sections 2.2.2 and 2.2.3).

##### 2.2.1.1 In Situ Transmission Electron Microscopy

In situ transmission electron microscopy (TEM) is one of the few techniques capable of directly visualizing catalyst structural evolution at the nanometer to atomic scale. Consequently, it plays an irreplaceable role in investigating the formation of active sites, dynamic phase transitions, and interfacial restructuring in eCO_2_RR. Benefitting from advancements in liquid‐phase electrochemical TEM (LP‐TEM/e‐LCTEM), researchers can track nanocrystal dissolution–re‐nucleation, oxide reduction, reversible transformations between crystalline and amorphous phases, and metal–interface phase flow behavior under near‐operando liquid and potential conditions, while observing nonequilibrium structural dynamics on millisecond‐to‐second timescales. These dynamic phenomena often directly determine the selectivity and stability of CO_2_ reduction, serving as the major factor in studying the structure‐activity‐failure relationship. More importantly, by integrating with selected area electron diffraction (SAD), energy‐dispersive spectroscopy (EDS), and other techniques, in situ TEM has progressed from taking “structural snapshots” to being a multidimensional platform for delivering structural dynamics data. It offers great support for constructing time‐resolved structural evolution maps of eCO_2_RR catalysts.

Taking palladium (Pd)‐based catalysts as an example, researchers have utilized in situ LP‐TEM and SAD techniques to systematically track the electrochemical‐induced phase transitions and subsurface species insertion behavior of Pd/PdH*_x_* catalysts under catalytic conditions [115]. By applying different electrode potentials (e.g., −0.2 V_RHE_), real‐time observations revealed that Pd particles underwent lattice expansion due to hydrogen absorption, forming the β‐PdH*_x_* phase, accompanied by morphological reconstruction and particle agglomeration, ultimately resulting in deactivation (e.g., particle detachment). Combined SAD analysis and DFT calculations revealed that subsurface *H in β‐PdH*_x_* favors CO_2_ hydrogenation at low overpotentials, promoting formate formation. At high potentials, the selective conversion to CO and H_2_ occurs, driven by changes in reaction energy due to electrode potential, rather than observed morphological or phase structural changes. Copper is a typical material capable of efficiently producing multicarbon (C_2+_) products in heterogeneous electrocatalysis, but the nature of its active sites under operating conditions remains under discussion. To elucidate the true active sites and their structural evolution, in situ electrochemical liquid‐phase scanning transmission electron microscopy (EC‐STEM) combined with time‐resolved techniques was employed to systematically investigate the structural evolution of Cu nanoparticles of different sizes (7, 10, and 18 nm) during the CO_2_RR process [116]. 7 nm Cu nanoparticles were reduced from an oxidized state to polycrystalline and disordered metallic Cu nanoparticles. The nanocrystalline grain boundaries were rich in coordination‐deficient active sites, promoting the key C─C coupling reaction, thereby exhibiting higher C_2+_ selectivity (approximately six times that of 18 nm particles). Notably, these active aggregated particles exist solely during the electrolysis process and are completely oxidized into single‐crystal Cu_2_O nanocubes upon exposure to air. Similarly, the structural reconstruction behavior of copper‐based bimetallic catalysts (Cu‐X, X = Ag, Fe, Zn, Pd) during the eCO_2_RR process and its influence on product selectivity were also observed using in situ e‐LCTEM technology [117]. For immiscible systems (such as Cu–Ag), selective copper dissolution‐redeposition occurred on the surface during the initial reaction stage to form Cu‐rich nanoparticles. This process evolves through nucleation, rapid growth, Ostwald ripening, and particle agglomeration, accompanied by pore formation and an increase in lattice defects (Figure 5b). It promotes *CO dimerization and enhances the spillover effect, significantly improving the selectivity ratio of C_2_H_5_OH/C_2_H_4_. In contrast, in miscible systems (such as Cu–Zn), Cu atoms are uniformly incorporated into the Zn lattice, with low defect density and strong structural stability. Consequently, the formation of sufficient Cu‐rich active sites is suppressed, and CO becomes the dominant product. This comparison clearly demonstrates that rational tuning of alloy miscibility can purposefully guide or inhibit Cu surface reconstruction under operating conditions, thereby synergistically regulating C_2+_ selectivity in concert with particle size.

Furthermore, e‐LCTEM was employed to elucidate the electrolysis mechanism of 2D Cu(II) oxide nanosheets (CuO‐NS) with (001) orientation [118]. CuO‐NS were gradually reduced and evolved into dendritic metallic Cu^0^ with undercoordinated active sites (Figure 5c). The abundant active sites in the dendritic structure promoted efficient formation of C_2+_ products. The Faradae efficiency (FE) of C_2_H_4_ reached 33% at neutral pH and exhibited long‐term stability. Additionally, e‐LCTEM can be used to reveal the dynamic synthesis mechanism of confined catalysts, guiding the design and synthesis of efficient nano‐confined electrocatalysts. Xiao et al. [119] employed this technique to study the intercalation and reduction of palladium ions between 2D black phosphorus nanosheets. Under an external electric field, palladium ions were driven to intercalate into the interlayer of black phosphorus nanosheets and were reduced to form palladium clusters, resulting in a significant expansion of the interlayer spacing. Guided by this mechanism, structurally stable Pd‐intercalated black phosphorus confined catalysts (Pd‐i‐BP) were successfully synthesized under ex situ conditions. The confined structure stabilizes reaction intermediates, exhibiting up to 90% formate selectivity and excellent cycling stability. This demonstrates the unique value of in situ TEM in studying synthesis mechanisms and structure–property relationships. Finally, innovative high‐resolution polymer liquid cells (PLCs) have overcome the spatial resolution limitations of traditional liquid‐phase TEM, enabling direct observation of atomic dynamics at electrified solid–liquid interfaces (ESLIs) under an electric bias [120]. The study found that during the Cu‐catalyzed CO_2_RR process, a fluctuating liquid‐like amorphous interface phase reversibly transforms between crystalline and amorphous states under an applied potential and flows along the crystalline Cu surface, thereby inducing surface reconstruction and atomic migration (preferentially occurring at atomic steps and crystalline–amorphous interfaces). The schematics are shown in Figure 5d. Combined with rapid cryo‐fixation and EDS/Electron energy loss spectroscopy (EELS) analysis, the interface phase was confirmed to contain Cu^0^/Cu^+^, C, H, and O, with its thickness positively correlated with C_2_H_4_ selectivity.

As can be seen, in situ TEM, with its high spatial resolution at the nanometer to atomic scale and real‐time imaging capability under reaction conditions, combined with diffraction, energy spectroscopy, and theoretical calculations, has become an indispensable platform for revealing dynamic structural evolution, analyzing reaction mechanisms, and guiding catalyst design.

##### 2.2.1.2 Surface Probe Techniques

While TEM emphasizes the morphology and phase transitions at the particle scale, surface probe techniques focus on atomic arrangements and adsorption layer structures. Identical grain atomic force microscopy (AFM) was used to systematically investigate the surface reconstruction behavior of copper catalysts in the eCO_2_RR process [121]. With changes in potential, the copper surface undergoes increased roughness, grain boundary movement, and the formation of new crystal faces. This process is associated with improved current density and Faraday efficiency within a particular potential window. Combined with electron backscatter diffraction (EBSD) analysis, the study revealed an increase in the relative abundance of the (111) and (212) crystal planes on the copper surface during the reconstruction process, with these two crystal planes exhibiting superior catalytic activity in CO_2_RR. Moreover, in situ AFM demonstrated greater potential, as it can track surface morphology evolution and quantify reconstruction extent using surface roughness changes (Δ*R*_a_). Yue et al. [122] demonstrated that in an acidified K_2_SO_4_ solution without HCO_3_^−^, the ΔR_a_ of the Cu catalyst was only 2.04 nm after −0.8 V electrolysis under a CO_2_ atmosphere, indicating only minor reconstruction. In KHCO_3_ solution containing HCO_3_^−^, ΔR_a_ reached 10.50 nm, accompanied by the generation of a large amount of Cu^+^, consistent with the oxidative radicals‐driven dissolution‐redeposition mechanism. This clearly reveals that Cu reconstruction relies on the presence of Cu^+^ in solution and occurs significantly only under electrolytic conditions containing HCO_3_^−^, which implies that the solvent/electrolyte composition holds a pivotal influence on surface reconstruction and subsequent catalytic behavior. Unlike electrolyte‐driven reconstruction, electrochemical gas chromatography atomic force microscopy (EC‐AFM) studies of the Cu(100) model catalyst under real conditions reveal that its surface structure is primarily potential‐driven [123]. At open‐circuit voltage (OCV), the surface forms an epitaxial Cu_2_O(111) plate‐like morphology. At −0.5 V_RHE_, the surface transforms into a smooth hill‐and‐valley structure accompanied by the emergence of a p(2×2) superlattice (favorable for the formic acid pathway). When the potential decreased to −1.0 V_RHE_, the surface exhibited rectangular step structures aligned along the <110> direction, revealing (1×1) metallic Cu surfaces (promoting the C_2+_ pathway). Quantitative analysis revealed that the density of coordination‐unsaturated sites (i.e., step edges) exhibited a non‐monotonic change with potential: from the rougher surface at OCV (line defect density ∼ 0.12 nm^−1^) to the smoother structure at −0.5 V_RHE_ (∼0.07 nm^−1^), followed by a rebound to ∼ 0.10 nm^−1^ at −1.0 V_RHE_ due to step orientation changes (C_2+_). These results directly confirm the dynamic correlation between the surface morphology, defect density, and potential of the Cu catalyst during the CO_2_RR process, providing a structural basis for understanding the selective changes in C_2+_ products at different potentials.

More interestingly, by combining in situ correlative AFM (c‐AFM) with lateral force microscopy (LFM) technology, Munz et al. [124] successfully obtained in situ correlated imaging of electronic transport and molecular interface structure on the surface of an Au–CuO*_x_* bimetallic model catalyst, establishing a key experimental method for revealing the microscopic mechanisms at solid–liquid interfaces. At the in situ and nanoscale, the electrical conductivity, chemical friction, and morphological characteristics of Au–CuO*_x_* for CO_2_RR were simultaneously investigated. (Figure 5e) In air, the local current on the Au surface was significantly higher than that on the CuO*_x_* islands, and the CuO*_x_* region exhibited significantly higher friction. In ultrapure water, the interfacial water dipole layer increases contact resistance. In 0.1 M KHCO_3_, the friction force comparison reverses, indicating that the specific adsorption of bicarbonate ions significantly alters the structure and order of the interfacial hydration layer, further regulating the interfacial electron transfer rate. Meanwhile, the electrical conductivity of Au grain protrusion regions is significantly superior to that of grain boundary regions, potentially due to enhanced local electric field effects. In addition, in situ EC‐AFM verified that the electrodeposition of a prototypical phenanthrolinium‐based organic thin film (∼12 nm thick) on a polycrystalline Cu surface markedly suppresses substrate morphology reconstruction during the CO_2_RR process [125]. Combined with other characterization techniques, it was confirmed that the organic membrane can retain approximately 13% of Cu^+^ species under strong reducing potentials (as low as −0.1 V) and maintain the Cu−O_ad_ characteristic signal for over 90 min, providing dual support for the morphology and chemical state of selectivity and stability.

In situ scanning tunneling microscopy (STM) can also track changes in surface structure and adsorption layers at the atomic scale. High‐resolution dynamic observations of the surface structure evolution of a Cu(100) in a 0.1 M KHCO_3_ solution were conducted [126]. As the potential was gradually reduced to −0.22 V, nanoscale Cu clusters began to appear, with a significant increase in cluster density at −0.27 V. As the potential was restored, the clusters gradually disappeared, indicating that the formation of nanoclusters is reversible to some extent. However, the formation of the clusters was accompanied by the irreversible destruction of the original ordered carbonate adsorption layer on the surface (Figure 5f). After potential restoration, only a short‐range ordered disordered adsorption layer formed, which remained relatively stable for several minutes to hours. This atomic‐scale evidence directly supports the *CO‐induced clustering and adsorption layer deconstruction mechanism, providing crucial insights into the dynamic restructuring of Cu electrodes during the initial stage of CO_2_RR and its influence on reaction pathways.

##### 2.2.1.3 Diffraction Analysis Methods

While atomic‐level imaging and interface‐localized probes present details of microstructure and surface reconstruction, a technique capable of tracing overall crystalline phase progression and structural stability across larger volumes and extended time scales remains desirable to compensate for the limitations of localized views. In situ X‐ray diffraction (XRD) provides statistical information on phase transitions and preferred orientations in large‐volume samples, complementing atomic‐level imaging. For example, the structural stability of CuO/SnO_2_ nanosheet heterojunctions in CO_2_RR was monitored [129]. As reaction progressed, the diffraction peaks of CuO gradually weakened, while those associated with metallic Cu appeared, indicating that CuO was reduced to Cu. Simultaneously, the SnO_2_ signals remained stable, suggesting that its crystal structure is largely preserved. A similar result was also observed in the CuO/In_2_O_3_ catalyst [130]. Such component‐selective phase transitions highlight the synergy and dynamic restructuring characteristics of different components in multiphase catalytic systems. By choosing structurally stable components as support or framework, it is possible to maintain the overall stability of the catalyst while allowing the active components to reconstruct to form active sites. The effect of surface modification on crystal plane exposure and catalytic activity can also be observed via in situ XRD. For example, dodecyl mercaptide (DDT)‐modified hierarchical nanostructured CuO electrodes (CuO‐SH) formed a richer Cu(100) crystal face during the reaction, with a Cu(100)/Cu(111) peak intensity ratio exceeding three times that of unmodified CuO [131]. This crystal plane ratio was positively correlated with C_2_H_4_ FE, proving that Cu(100) plays a key role in the C−C coupling, ultimately achieving a maximum C_2_H_4_ FE of 79.5% with excellent stability.

Doping strategies can also significantly influence catalyst performance by regulating crystal structure. Taking Al‐doped Cu layered double hydroxides (Cu_3_Al‐LDHs) as an example [127], upon application of a reducing potential, the initial (003) diffraction peak (23.4°) reconstructed into Cu_2_O‐dominated crystal phases, along with the enhancement of Cu_2_O (111), (200), and (220) peaks. It is noted that the introduction of Al helps to stabilize Cu^+^ species during the reaction process. Conversely, Cu‐LDHs were directly reduced from Cu_2_(OH)_2_CO_3_ to metallic Cu (Figure 5g). The results indicate that the introduction of Al hinders the complete reduction and reconstruction of Cu, favoring retention of active Cu^+^, thereby regulating the electronic structure of the catalyst and promoting C−C coupling, which enhances selectivity and FE of C_2+_ products. Similarly, lattice compression of Cu‐doped Ag nanosheets (AgCu) catalysts was identified by XRD [132]. As the Cu doping ratio increases, the lattice compression of AgCu rises from −1.90% (AgCu_3%_) to −2.75% (AgCu_6%_). Under −0.8 V_RHE_, the peak positions and intensities of AgCu_5%_ remained stable over 80 minutes in continuous reaction, without noticeable reconstruction or phase transition. This demonstrates excellent catalyst stability.

Beyond copper‐based catalysts, bismuth (Bi)‐based catalysts also display remarkable structural evolution characteristics [133]. Specifically, the regulatory effects of different halogens on reduction kinetics and crystal plane evolution in Bi‐based oxyhalides (BiOX, X = Cl, Br, I) catalysts were investigated [134]. Under OCV, each BiOX presented its characteristic diffraction peaks. After applying a reduction potential, BiOBr was rapidly converted to metallic Bi at −1.15 V_RHE_, quickly forming a crystal structure dominated by Bi(003) and (006) planes. BiOI was reduced more slowly, exposing multiple Bi planes, while BiOCl required a more negative potential to complete reduction, primarily exposing Bi(012) planes. These differences show that the type of halide not only affects the onset potential, phase transformation rate, and final facet configuration, but also governs catalytic performance. Owing to its faster phase transformation and favorable facet exposure, BiOBr exhibits the highest formate selectivity.

Utilizing operando synchrotron 2D‐XRD, the structural changes and active crystal plane evolution of CdO@ZnY during the eCO_2_RR process were precisely tracked [128]. Zn‐doped Y‐type zeolite molecular sieves (ZnY) retained the typical crystallographic features of Y‐type molecular sieves, indicating that Zn successfully replaced Al sites in the framework without disrupting the molecular sieve framework. The introduced CdO nanoclusters (∼1.2 nm) are uniformly distributed in dodecagonal ring supercages of ZnY, with their (111) and (200) crystal planes clearly identifiable. As reaction progresses (Figure 5h), the diffraction peaks of the ZnY framework remained stable, while the (200) peak of CdO gradually decayed and the (111) became dominant, indicating that this crystal plane was the truly active surface for CO_2_ reduction intermediates. This structural evolution matched theoretical calculations well, revealing stability and changes in the active sites of the catalyst during reaction, providing important structural evidence for its excellent electrocatalytic performance.

#### 2.2.2 Electronic States and Coordination Structure Detection

**FIGURE 6:**
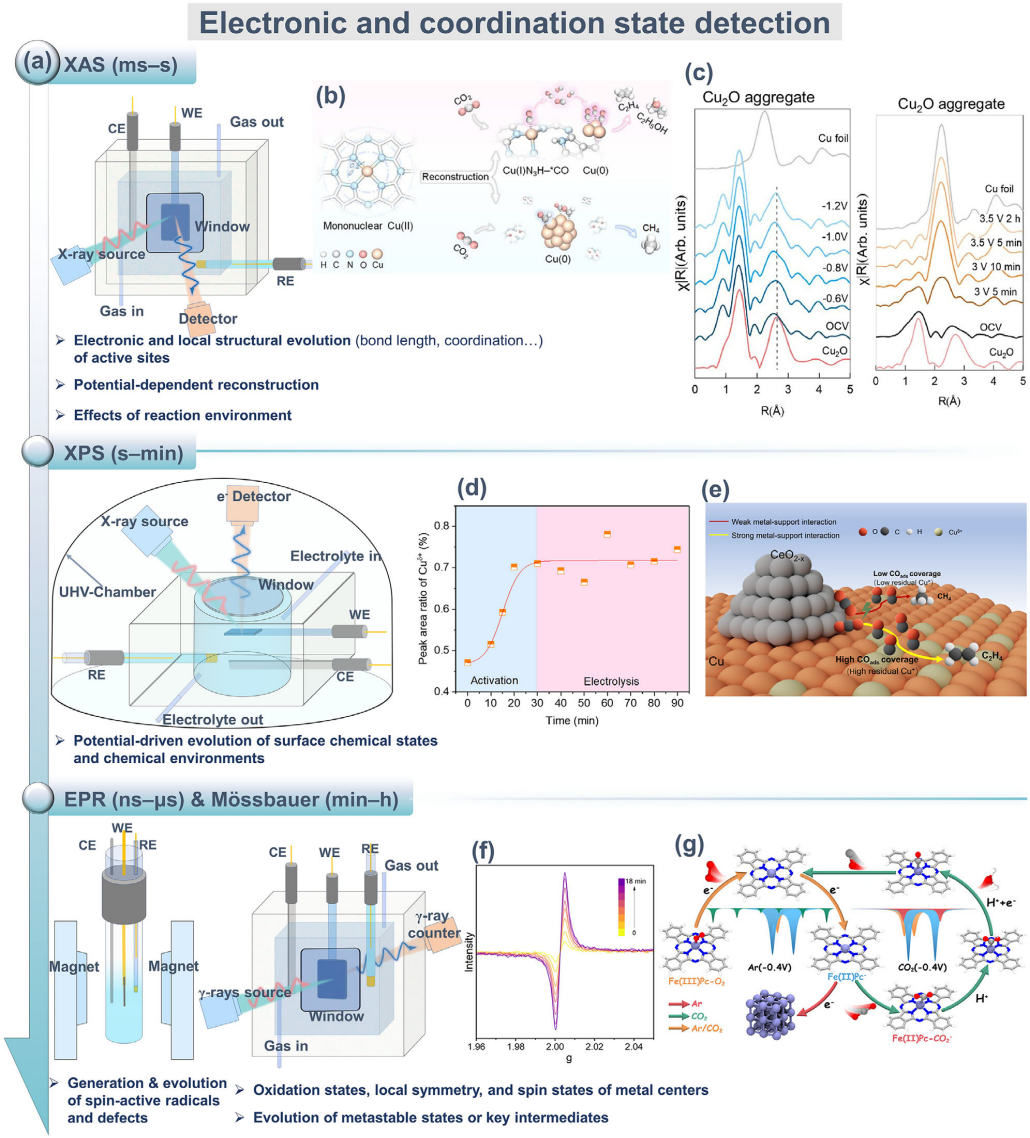
(a) XAS, XPS, EPR, and Mössbauer spectroscopy for electronic and coordination state detection analysis. (b) Mechanism of catalyst function. Reproduced with permission [144]. Copyright 2025, American Chemical Society. (c) FT‐EXAFS of Cu_2_O aggregates in H‐type cells and in MEA cells. Reproduced with permission [147]. Copyright 2024, The Author(s). (d) Area ratio of Cu^δ+^ on Cu_300_‐Act. Reproduced with permission [149]. Copyright 2025, The Author(s). (e) Schematic of selective promotion of C_1_ and C_2+_ formation in the CO_2_RR process by MSI. Reproduced with permission [151]. Copyright 2024, American Chemical Society. (f) In situ EPR spectra during pre‐reduction for P‐Cu_2_O. Reproduced with permission [152]. Copyright 2025, American Chemical Society. (g) Proposed dynamic evolution of Fe species in CO_2_ and Ar. Reproduced with permission [153]. Copyright 2023, American Chemical Society.

At electrochemical interfaces, valence transitions of active centers, reconstruction of coordination environments, and changes in d‐orbital occupation collectively affect electron transfer pathways and intermediates stability. It is vital to understand these electronic structure evolutions for elucidating the eCO_2_RR mechanism. Since such changes typically occur at solid–liquid–electric field interfaces and display transient properties, a synergistic characterization of in situ and quasi‐in‐situ spectroscopic techniques is required to capture these fleeting structural signals. (Figure 6a) X‐ray absorption spectroscopy (XAS) analyzes the near‐edge absorption structure (XANES) and extended fine structure (EXAFS) to quantitatively obtain information on valence state transitions of metal centers, reorganization of their local coordination configurations, and bond length variations [135, 136]. Owing to the excellent temporal resolution of synchrotron radiation sources, it is suitable for capturing the dynamic processes of short‐lived intermediate states [137]. Surface‐sensitive in situ/quasi‐in‐situ X‐ray photoelectron spectroscopy (XPS) provides insights into the valence states and elemental chemical environments of catalysts at reaction interfaces, thereby linking electronic structural changes to adsorption properties and reaction selectivity [138]. In situ electron paramagnetic resonance spectroscopy (EPR) provides unique electronic spin information for radical‐type intermediates containing unpaired electrons (*CO_2_^−^ radicals, ·COOH), revealing the electronic transfer characteristics of key intermediates [139, 140]. Furthermore, for systems containing Mössbauer‐active nuclei (e.g., Fe and Sn), Mössbauer spectroscopy offers extremely high energy resolution to distinguish different valence states, electron density distributions, and local symmetries to precisely characterize the metastable structures of catalytic centers during the reaction [141].

##### 2.2.2.1 In Situ X‐Ray Absorption Spectroscopy (XAS)

By continuously monitoring changes in the valence state and coordination structure of catalysts, in situ XAS supplies vital evidence for explaining the progression from precursor catalysts to true active states of metal centers. The most extensively studied class of catalysts is Cu‐based materials. Liu et al. [142] utilized in situ X‐ray absorption fine structure spectroscopy (XAFS) to prove that alkali‐cation‐induced cathodic corrosion leads to the formation of electron‐rich Cu intermediates, which in turn trigger irreversible structural reconstruction of Cu catalysts. In fact, Cu‐based catalysts typically exhibit typical potential‐dependent structural rearrangement throughout the catalytic process. In Cu–Ni heteronuclear atom catalysts (CuNi‐HAC) [143], Cu K‐edge XANES spectra combined with EXAFS fitting reveal that Cu atoms in the pristine catalyst were coordinated in a Cu–S_1_N_3_ configuration and form heteronuclear dual‐atom sites with Ni (Cu–S_1_N_3_/Ni–S_1_N_3_). As the potential was shifted negative, the average oxidation state of Cu continuously decreased from +1.8, accompanied by a gradual reduction in Cu−N/C coordination numbers and a concomitant increase in Cu−Cu/Ni coordination, indicating potential‐driven aggregation of Cu atoms. Subsequently, Cu_5_−Ni atomic clusters emerge as the dominant species and further evolve into larger Cu_7_−Ni clusters at −1.2 V. Among these, the Cu_5_−Ni cluster was identified as the key active structure during catalysis. Its formation was associated with cooperative regulation of the Cu oxidation state and coordination environment, leading to a pronounced enhancement in catalytic performance. It can be seen that the stability and activity of Cu single‐atom catalysts are also influenced by the neighboring coordination environment and external substituents. Catalysts with different coordination structure models, represented by CuPc (first and third shell nitrogen coordination), CuTPP (first shell nitrogen coordination, Cu−N_4_), and CuPAN (first shell nitrogen and oxygen coordination, Cu‐N_2_O_2_), were used to investigate the influence of neighboring coordination shells on the reconstruction behavior of copper unit sites [144]. CuPc‐F (fully fluorine‐substituted) and CuPc‐NH_2_ (tetra‐amino‐substituted) were used to investigate the mechanism of external substituents. CuPc undergoes partial reconstruction at −0.6 V_RHE_, with the appearance of Cu(0)−Cu bonds. At −1.2 V_RHE_, the Cu(0) ratio was 27%. In contrast, CuTPP maintained structural stability over the entire reaction window. CuPAN underwent rapid and complete reconstruction into large‐sized Cu(0) clusters, confirming that the planar Cu–N_4_ configuration possesses markedly higher structural stability than the Cu–N_2_O_2_ configuration. For catalysts with different external substituents, the electron‐donating group CuPc‐NH_2_ promotes the transformation of copper units into Cu(0) clusters, while the electron‐withdrawing group CuPc‐F stabilizes Cu(I) sites and preserves their coexistence with small‐sized Cu(0) clusters (Figure 6b). Electrocatalytic results indicated that the in situ generated Cu(0)/Cu(I) is the actual active phase for carbon product formation and exhibited a strong correlation with the formation of C_1_ and C_2_ products.

Additionally, the enhanced selectivity mechanism for C_2+_ products achieved by combining organic molecular modification strategies with protective layer design was also validated via in situ XAS [145]. Beyond investigations focused solely on the catalyst itself, operando XAS has also provided critical insights into the influence of impurities in actual industrial applications. Targeting common contaminants in industrial CO_2_ feeds, such as NO*_x_* and CN^−^ species, researchers combined operando XAS with DFT calculations to elucidate both the working mechanism of Ni single‐atom catalysts (NiSACs) during eCO_2_RR and the transient poisoning behavior induced by impurities, including NO_2_^−^ and SCN^−^ [146]. Under reducing potentials, Ni consistently retained a tetragonal‐planar Ni−N*_x_* coordination geometry with a stable valence state. While the distance of the first coordination layer of Ni−N remained constant, the average distance of the second coordination layer slightly increased, confirming the structural stability of the active site. Spectral changes associated with electron transfer from the Ni 3d*z_2_* orbital to the LUMO orbital of CO_2_ were observed. DFT calculations further validated this electron transfer pathway and indicated that impurity NO_2_^−^ can bind and be reduced at lower overpotentials. The dynamic binding of SCN^−^ with Ni sites (with a longer binding time than CO_2_) leads to reversible poisoning, i.e., activity recovery after impurity removal. Collectively, these findings provide operando structural evidence for evaluating the tolerance of single‐atom catalysts toward poisoning in complex reaction environments. Note that even the same catalyst may exhibit different structural evolution in different electrolytic cells. Choi et al. [147] investigated the morphological reconstruction of three different Cu‐based catalysts (Cu_2_O nanocubes, Cu_2_O nanoclusters, and commercial Cu (cCu) nanoparticles) in MEA cells and H‐type cells, as well as their effects on C_2+_ product selectivity. In the H‐type cell, Cu_2_O cubic catalysts coexist in both metallic Cu^0^ and Cu_2_O phases, with a relatively stable structure. Cu_2_O agglomerates largely retain their original Cu_2_O state (Figure 6c), indicating slow reduction kinetics, which is conducive to the formation of C_2+_ products. In contrast, in MEA cells, Cu_2_O cubic and cCu were more prone to reconstitution, being completely reduced to metallic Cu and forming interconnected nano‐copper networks; this structure exhibited higher selectivity toward C_2+_ compounds. Cu_2_O aggregates exhibited a mixed phase of Cu and Cu_2_O (Figure 6c), accompanied by morphological transformations forming defect‐rich hollow nanoparticles. However, during long‐term operation in the MEA, both Cu_2_O cubes and cCu decomposed into small Cu nanoparticles, leading to catalyst deactivation over time. It highlights the trade‐off between selective enhancement and structural stability under high reaction intensity.

In situ XAS not only revealed potential‐driven dynamic evolution of the catalyst's electronic states and local structure but also clarified key regulatory roles of local coordination environment, electronic effects, and reaction environment on the catalyst's stability and activity. These systematic studies have laid a solid structural foundation for understanding and designing efficient and stable electrocatalysts for CO_2_ reduction and provided important theoretical guidance and experimental strategies for the functionalization design of future catalysts.

##### 2.2.2.2 In Situ/Quasi‐In‐Situ X‐Ray Photoelectron Spectroscopy (XPS)

In situ and quasi‐in‐situ XPS focuses on the electronic valence states and chemical environment of the catalyst surface, further complementing the macroscopic information on the overall coordination structure provided by XAS. For example, by directly tracking the changes in Cu valence states under reaction conditions, researchers revealed formation, stability, and transformation mechanisms of Cu^δ+^ (1 < δ < 2) species in the CO_2_RR of various Cu‐based catalysts, and clarified their close relationship with C_2+_ product selectivity [148]. Shao's group [149] successfully constructed a catalyst rich in Cu^δ+^ active sites (Cu*_x_*‐Act, *x* denotes annealing time) through the synergistic effects of thermal annealing and electrochemical activation in the study of Cu‐MOF‐derived catalysts. Quasi‐in‐situ XPS quantitative analysis (Figure 6d) revealed that in the first 10 min of activation, Cu^2+^ significantly converted to Cu^+^, with the Cu^δ+^ ratio rapidly increasing from 0.4 to 0.7 and remaining stable throughout CO_2_RR process. This highly stable Cu^δ+^ directly promotes Cu−O dimerization and *C−C coupling, resulting in higher C_2+_ product FE (78±2% vs. 45%) and partial current density (−46 vs. −24 mA cm^−2^) compared to unactivated samples, thereby achieving high selectivity and activity toward C_2+_ products. A similar phenomenon was observed in Ce‐doped Cu oxide (Ce/CuO*_x_*) [150]. In situ XPS revealed that during the reaction, a portion of Cu^2+^ was reduced to Cu^+^ while the overall Cu species remained in a Cu^δ+^ state. This partial reduction is attributed to a shift of the Ce binding energy toward lower values, facilitating electron transfer from Cu to Ce and enabling dynamic Ce^3+^/Ce^4+^ interconversion, which prevents complete reduction of Cu^δ+^. Under −1.2 V_RHE_, the FE for C_2+_ products reached 60% (C_2_H_4_ 40%, CH_3_CH_2_OH 14%, CH_3_COOH 6%), with stable operation maintained over 25 h, demonstrating excellent catalytic performance.

In the study of CeO_2_‐modified Cu_2_O nanocube catalysts, precise analysis combining quasi‐in‐situ XPS with Cu LMM Auger spectra further indicated that the CeO_2−_ *_x_* carrier not only stabilizes residual Cu^+^ at the interface during the CO_2_RR process but also retains its quantity depending on the strength of the metal–carrier interaction (MSI) (Figure 6e) [151]. After reaction, the pure Cu_2_O retained only 4.8% ± 1.5% Cu^+^ on its surface, while Cu_2_O–CeO_2_‐w (hydrothermal synthesis) and Cu_2_O–CeO_2_‐s (thermal oxidation‐reduction preparation) retained 9.3% ± 3.2% and 20.5% ± 3.8% Cu^+^, respectively. The MSI formed by thermal oxidation‐reduction (s samples) was stronger than that formed by direct deposition (w samples), thereby retaining more Cu^+^ species. The Cu_2_O–CeO_2_‐s with a higher Cu^+^ ratio exhibited higher selectivity (52% ± 0.57%) in C_2_H_4_ production, while the Cu_2_O–CeO_2_‐w with a lower Cu^+^ ratio tended to produce CH_4_ (40% ± 1.4%), directly demonstrating the positive correlation between surface Cu^+^ stability and C_2+_ product selectivity.

Overall, these in situ and quasi‐in‐situ XPS studies demonstrate that generation and stability of Cu^δ+^ species not only depend on the initial structure and activation mode but are also closely related to electronic regulation of carrier and metal‐carrier interactions; long‐term maintenance of Cu^δ+^ is crucial for achieving high selectivity and stability of C_2+_ products in CO_2_RR.

##### 2.2.2.3 Electron Paramagnetic Resonance (EPR)

For free radicals and short‐lived intermediates containing unpaired electrons, EPR spectroscopy, with its unique ability to detect electron spin, can directly capture key species during reactions, providing crucial evidence for elucidating the mechanisms of electrocatalytic reactions. In a typical carbonic acid electrolyte, in situ EPR directly captured the generation and evolution of highly oxidative hydroxyl radicals (OH•) [154]. In CO_2_‐saturated KHCO_3_ solutions, 5,5‐dimethyl−1‐pyrroline N‐oxide (DMPO) spin trapping experiments showed that DMPO‐OH signal continued to increase after 20 min, while DMPO‐H signal weakened, indicating that OH• radicals were continuously generated during the reaction. Temperature and HCO_3_^−^ concentration influence OH• generation. When the temperature exceeds 20°C, the OH• radical signal increases. When the HCO_3_^−^ concentration is less than 0.1 M, the amount of OH• generated increases with increasing HCO_3_^−^ concentration, but decreases in the range of 0.1–3.0 M. Corrosion experiments indicate that OH• radicals in KHCO_3_ solutions cause rapid oxidation and significant corrosion of copper electrodes, thereby controlling the dynamic equilibrium of Cu^2+^/Cu^0^ and promoting the formation of C_2+_. The widespread presence of solution‐phase radicals has sparked interest in their interactions with catalyst structures. For example, a CuAg catalyst with a grain boundary oxidation structure was constructed by an electric shock (ES) reduction strategy [155]. In situ EPR detected H signals at −0.8 V_RHE_, and after switching to OCP, stable OH• signals were detected and persisted for 1.5 h without decay. OH•, as a strong oxidizing agent, causes rapid oxidation of Cu, forming Cu^δ+^ active sites. Combined with in situ XAS, it was found that the grain boundary oxidation structure effectively suppresses the complete reduction of Cu^δ+^ at high cathodic potentials, achieving 50% CH_4_ FE. The results indicate that specific structures can utilize solution‐phase radicals to achieve valence state regulation and performance enhancement.

In addition to radicals, EPR can also directly track the dynamic behavior of defects during the catalytic process. For example, by introducing oxygen vacancies (OVs) into Cu_2_O lattice via a phosphorus (P)‐doped intermediate strategy, a FE of 87% for C_2+_ and a partial current density of 347.8 mA cm^−2^ were achieved, with stable operation in an MEA for 30 h [152]. In situ EPR measurements (under −0.5 mA pre‐reduction conditions) revealed that OVs persist and their concentration increases with reduction time, confirming the dynamic generation of OVs (Figure 6f). Quasi‐in‐situ EPR of the post‐reaction samples further indicated that high OVs concentrations are maintained throughout the catalytic process. Combined with other characterization analyses, the breaking‐reforming of P−O bonds confer dynamic regulation capabilities to OVs, enabling them to remain stable at high concentrations. This significantly enhances the adsorption and conversion of *CO intermediates, ultimately improving the selectivity of C_2+_ products and suppressing by‐product formation.

EPR not only revealed the generation patterns of solution‐phase free radicals such as OH• in CO_2_RR electrolytes and their influence on the valence state of the electrode surface but also elucidated the synergistic mechanism between free radicals and specific catalyst structures, as well as the generation, stability, and performance correlation of defects during the reaction process. These studies collectively demonstrate that multi‐scale tracking of radical and defect dynamics via in situ/quasi‐in‐situ EPR is a core approach to understanding the coupled regulation mechanisms of activity and stability in catalysts (especially Cu‐based catalysts).

##### 2.2.2.4 Mössbauer Spectroscopy

Mössbauer spectroscopy provides element‐specific in situ electronic state and coordination environment information for catalysts containing Fe or Sn elements, addressing the limitations of traditional spectroscopic methods in distinguishing such details. This further enhances the precise characterization of the metastable structures of catalytic centers. In Fe‐based single‐atom catalysts, ^57^Fe Mössbauer spectroscopy revealed the reversible valence state regulation and coordination changes of the Fe−N_4_ active centers during catalysis. For example, Zeng and his colleagues systematically investigated the dynamic evolution of active sites in FePc‐CNT single‐atom catalysts [153]. The high‐spin (HS) Fe(III) species in O_2_–Fe(III)Pc gradually reduces to two HS Fe(II) species at negative potentials, corresponding to Fe(II)Pc and Fe(II)Pc‐CO_2_ intermediates with axial CO_2_ adsorption (Figure 6g). Signal of Fe(II)Pc‐CO_2_ increases with increasing potential and disappears upon removal of potential, indicating its role as a key intermediate in the CO_2_RR. Additionally, FeN_4_ sites partially convert to metallic Fe in the absence of CO_2_, suggesting that CO_2_ adsorption stabilizes the Fe(II)Pc species. This study highlights the unique ability of Mössbauer spectroscopy to quantitatively analyze the electronic structure of single‐atom centers under dynamic conditions. Similar dynamic evolution was also observed in Sn‐based systems. In situ ^119^Sn Mössbauer spectroscopy was used to identify Sn‐containing metastable structures generated by single‐atom tin in copper oxide carriers (Sn_1_‐CuO) during operation [156]. Sn^4+^ sites dynamically transformed from the initial Sn^4+^−O_4_−Cu^2+^ to a metastable Sn^4+^−O_3_−Cu^+^. This metastable state promoted the stable existence of neighboring Cu^+^ sites and enhanced the desorption process of *CO. At −0.8 V_RHE_, the catalyst achieves 98% FE_CO_. This valence‐coordination regulation mechanism implies that the metastable structure not only reflects the catalyst's transient configuration but may also be a vital factor in the reaction pathway.

In addition, rapid freeze‐quench (RFQ) Mössbauer spectroscopy of ^119^Sn has also been employed in recent years to track the dynamic evolution of specific catalysts. For example, Chen et al. [157] reported that a single‐atom copper‐modified tin disulfide catalyst (Cu_1_/SnS_2_) achieved ∼90.9% FE_HCOOH_ at 158 mA cm^−2^. This was primarily due to the gradual reduction of SnS_2_ to metallic Sn and SnS as the voltage increased. When the voltage reached −1.4 V, the content of Sn reached 74.2%, while the SnS_2_ content dropped to 17.5%. The final reduction of the more active Cu_1_/Sn promoted formation of the *CO_2_^−^/*OCHO intermediate, thereby enhancing selectivity toward HCOOH. Similarly, Zhang et al. [158] monitored the dynamic evolution of tin species in Ag‐modified SnS_2_ hollow microbox (Ag@SnS_2_). At OCV, the spectrum exhibited a double‐peak of SnO_2_ (Sn^4+^) and SnS_2_ (Sn^2+^). As potential gradually increased, the contents of Sn^4+^ and Sn^2+^ decreased, while the signal of Sn^0^ increased. At −0.8 V, the formation of Ag_3_Sn and Sn was observed. Finally, at −0.8 V, a high FE of 92.4% and current density of 97.3 mA cm^−2^ were achieved for CO_2_‐to‐formate, with working in a flow cell for 8 h, yielding FE_formate_ ≥ 95% and *j*_formate_ ≥ 220 mA cm^−2^.

In summary, Mössbauer spectroscopy can precisely analyze the valence state transitions, coordination environment changes, and structural rearrangement processes of active centers under working, and is highly consistent with theoretical calculations. This technique not only reveals synergistic effects and interface configuration stability in multi‐metal systems but also captures transient intermediates, providing indispensable spectroscopic support for exploring the mechanism of electrocatalytic CO_2_ reduction reactions and improving catalyst performance.

#### 2.2.3 Interface Reaction Intermediates and Dynamic Process Tracking

**FIGURE 7:**
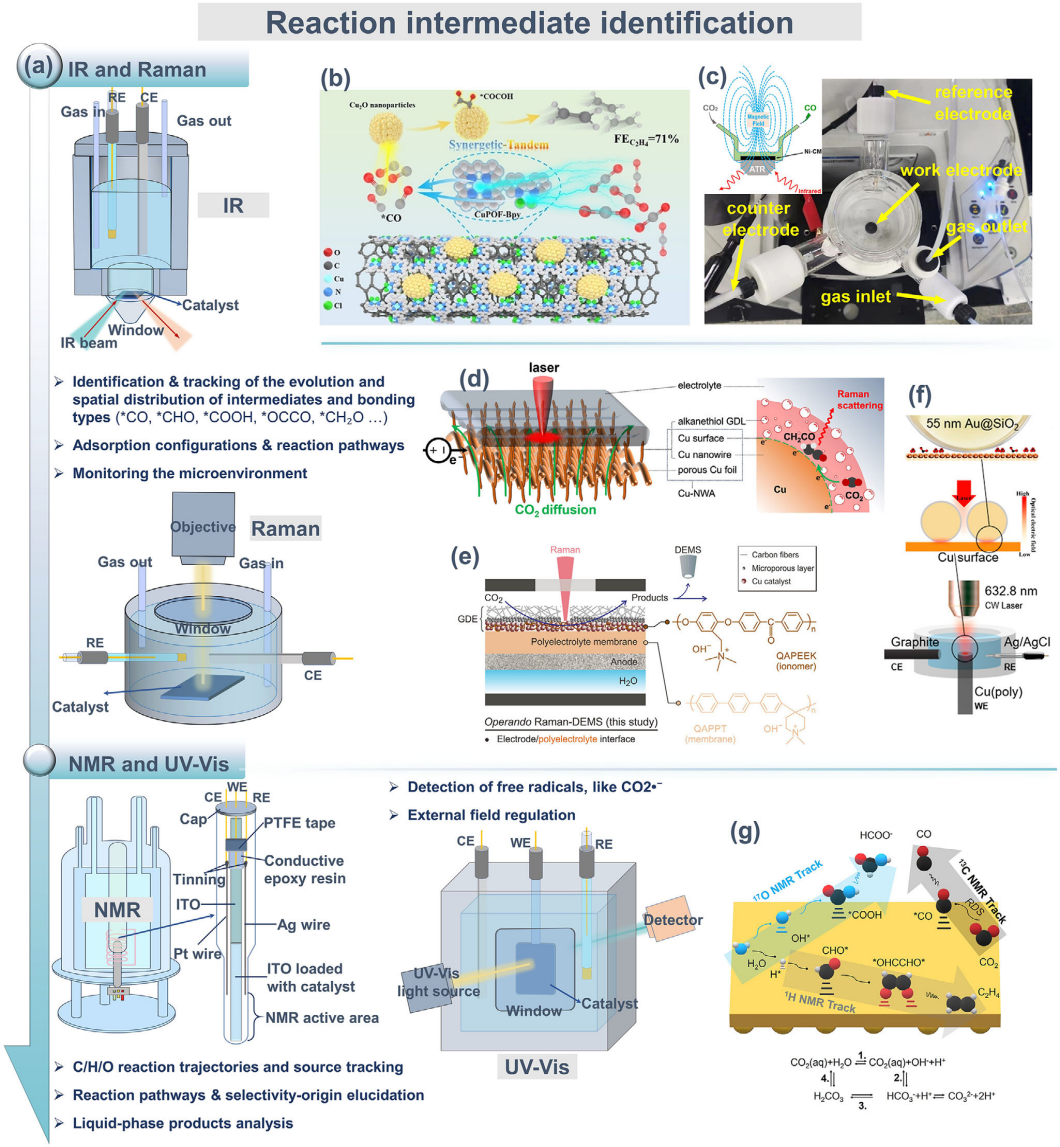
(a) IR, Raman, NMR, and UV–Vis spectroscopy for reaction intermediate identification analysis. (b) Schematic of the preparation of CuPOF‐Bpy/Cu_2_O@CNT and the pathway promoting C_2_H_4_ production. Reproduced with permission [160]. Copyright 2024, Wiley‐VCH GmbH. (c) In situ ATR‐SEIRAS device with magnetic field and corresponding schematic. Reproduced with permission [163]. Copyright 2024, The Author(s). Published by Elsevier B.V. (d) Schematic of Cu‐GDL‐electrolyte three‐phase interface of Cu‐NWA in in situ Raman reactor. Reproduced with permission [166]. Copyright 2025, The Author(s). (e) Schematic of operando cells for CO_2_RR. Reproduced with permission [169]. Copyright 2025, Wiley‐VCH GmbH. (f) Schematic of in situ electrochemical SHINERS. Reproduced with permission [171]. Copyright 2025, The Authors. (g) In situ electrochemical NMR for elemental tracking in CO_2_R reactions. Reproduced with permission [172]. Copyright 2024, Elsevier Inc.

In electrocatalytic reactions, structural identification and dynamic tracking of interface‐adsorbed intermediates are central to elucidating reaction pathways and mechanisms governing product selectivity. In situ spectroscopic techniques using molecular vibrations or electronic transitions as probes can capture characteristic signals of short‐lived species and reveal their association with the active surface structure of catalysts, thereby providing direct spectroscopic evidence for constructing reaction mechanisms. Current mainstream testing techniques include (Figure 7a): in situ Raman spectroscopy, in situ infrared spectroscopy, in situ nuclear magnetic resonance (NMR) spectroscopy, and in situ ultraviolet–visible spectroscopy (UV‐Vis).

##### 2.2.3.1 In Situ Infrared Spectroscopy

Infrared techniques represented by surface‐enhanced infrared absorption spectroscopy (SEIRAS) and Fourier transform infrared spectroscopy (FTIR) can identify key adsorption species such as *COOH, *CO, *CHO, and *OCCO at the molecular level and analyze their formation and transformation behavior under potential control.

For the identification of *CO and its coupled intermediates, Feng et al. [159] utilized an in situ attenuated total reflection surface‐enhanced infrared absorption spectroscopy (ATR‐SIRAS) system in their study of La‐doped copper hollow spheres (La‐Cu HS), clearly capturing the *CO adsorption peak at 2120 cm^−1^, which significantly enhanced with increasing negative potential (−1.1 to −1.3 V). On Cu HS, this peak was weak and only became significant at more negative potentials. Concurrently, the *OCCO corresponding to the C─C coupling intermediate at 1594 cm^−1^ showed an increased area ratio relative to the *CO peak with increasing negative potential. These spectroscopic results reveal that doping and porous structure regulation modulate the catalyst surface microenvironment, promoting the *CO generation and subsequent C─C coupling, thereby enhancing the selectivity and activity of CO_2_ reduction to polycarbonate products in an acidic system. Subsequently, on the Cu_3_N‐Ag nanocube catalyst, in situ ATR‐FTIR was employed to identify that it facilitates both the generation and stabilization of *CO [90]. This promotes the formation of *CHO and subsequent C−C coupling, significantly enhancing the selectivity of C_2_H_4_ and providing molecular‐level evidence for the C−C coupling mechanism in multi‐site catalysts. He et al. [160] utilized in situ ATR‐FTIR to systematically monitor key reaction intermediates on the surface of CuPOF‐Bpy/Cu_2_O@CNT tandem‐synergistic catalyst at −1.1 V_RHE_. Compared to control group, the *CO and *COCOH signals are stronger on this catalyst, indicating that the synergistic effect of dual active sites enhances CO production rates and C−C coupling ability, ultimately promoting efficient ethylene generation (Figure 7b). This study validates the effectiveness of the synergistic‐serial strategy in the synthesis of multi‐carbon products.

To investigate the regulation of intermediate formation by material structure, Wang et al. [161] employed in situ ATR‐SEIRAS to reveal the generation and accumulation of *CO (2063 cm^−1^) and C−C coupling intermediate *OCCOH (1543 cm^−1^) under different potentials in the Cu_2_OSO_4_@CuO catalyst. The results showed that sulfur doping stabilized the Cu_2_O structure, regulated the electronic properties of Cu, significantly promoted the stability of *CO and the asymmetric C−C coupling reaction, thereby effectively increasing the yield of C_2+_ products, and ultimately achieving 88% C_2+_ FE and a partial current density of 609 mA cm^−2^. In the field of molecular catalysis, in situ diffuse reflect infrared spectroscopy (DRIFTS) was used to monitor the CO_2_RR process of o‐Cu‐Por‐Sa (αβαβ) and o‐Cu‐Por‐Sa (αααα), capturing the adsorption and conversion of *COOH and *CO intermediates [162]. Symmetrically distributed hydroxyl ligands (αβαβ isomers) regulate the intermediates (*CO, *CH_2_O) via non‐covalent hydrogen bonds, lowering the reaction energy barrier and enhancing methane yield. While hydroxyl‐aggregated isomers (αααα) restrict CO_2_ adsorption and expose *CO, leading to CO as the primary product. This work deepens the understanding of how noncovalent interactions between molecular catalyst ligands influence selectivity.

In situ infrared spectroscopy not only captures static adsorption intermediates but also enables real‐time monitoring of the effects of external fields on dynamic interface structures and reactant species during catalytic reaction. Recently, Wei et al. [163] designed an in situ ATR‐SEIRAS device equipped with a magnetic field to investigate the effect of magnetic fields on eCO_2_RR (Figure 7c). They observed that nickel‐based catalysts (Ni‐CM) with both magnetic responsiveness and electrocatalytic CO_2_ reduction activity exhibited a red shift of *COOH peak under magnetic field, indicating that the magnetic field increased C−O bond length in *COOH, facilitating its cleavage to form CO. Combining DFT and Bader charge analysis, it was found that the magnetic field reduced Gibbs free energy barrier of the reaction, altered the charge distribution of key atoms in *COOH, thereby enhancing reaction activity. This perfectly demonstrates that the magnetic field, as an external regulatory tool, can effectively modulate the structure of key intermediates and reaction pathways, thereby enhancing catalytic efficiency. Coincidentally, Ye et al. [164] combined in situ ATR‐SEIRAS with online DEMS to study pulsed eCO_2_RR, and observed the generation process of formate intermediate (*OCHOM, 1674 cm^−1^) on an Ag electrode at the molecular level. Meanwhile, on Cu electrodes, pulsed potentials accelerated the consumption rate of *CO_L_ intermediates, promoted C−C coupling reactions, and drove the FE of C_2+_ products to 77.8%. In situ infrared spectroscopy simultaneously revealed the dynamic changes of key adsorbed species such as *CO, *CHO, and *OCHO at different potentials. Combined with mass spectrometry monitoring of products, it was clarified that pulsed potentials synergistically suppress HER by regulating the electrode surface structure and local CO_2_ concentration, effectively optimizing product distribution and selectivity. This work provides important mechanistic support for the application of pulsed regulation strategies in electrocatalysis. It is apparent that to comprehensively understand complex electrocatalytic processes, integrating in situ infrared spectroscopy with other characterization techniques has become a prevailing trend. This strategy can reveal multidimensional information on catalyst structure, surface adsorption, and reaction products, providing a scientific basis for the design of efficient industrial electrolytic systems.

In situ infrared spectroscopy, with its molecular‐level sensitivity, has emerged as a crucial tool for revealing key adsorbed intermediates and their dynamic changes in electrocatalytic processes. When combined with external field regulation (e.g., magnetic fields) and synergistic application of other techniques, it not only deepens our understanding of catalytic mechanisms but also provides precise guidance for the design of efficient catalysts, thereby advancing the development of electrocatalytic CO_2_ reduction and related energy conversion technologies.

##### 2.2.3.2 In Situ Raman Spectroscopy

In situ Raman spectroscopy effectively identifies the formation pathways of polycarbonate products by capturing the stretching vibration peaks of key chemical bonds such as C−C, C−O, and M−C, and analyzes the correlation between these pathways and the oxidation state or local structure of the catalyst surface [165]. With the development of technology, in situ Raman spectroscopy has played an increasingly important role in revealing the dynamic changes, spatial distribution, and reaction microenvironment regulation of intermediate species on the catalyst surface in eCO_2_RR. Real‐time changes in intermediates reflect critical stages of the reaction pathway. Sun et al. [166] constructed a hydrophobic gas diffusion electrode based on copper nanowire arrays (Cu‐NWA) and utilized in situ Raman spectroscopy enhanced by structural resonance and charge transfer resonance to achieve high‐sensitivity dynamic monitoring of intermediates at industrial‐scale current densities (Figure 7d). The experiment directly captured the systematic evolution of top‐adsorbed CO (CO_‐top_), bridge‐adsorbed CO (CO_‐bridge_), and physisorbed CO (CO_‐physisor_) signals with current density during the reaction process, revealing that CO signals weaken at low current densities, CO_‐top_ converts to CO_‐bridge_ and accumulates at moderate current densities, and stable CO_‐physisor_ rapidly disappears at high current densities. The appearance and transformation of important intermediates, such as CH_2_, CO/CH_2_ co‐adsorbed states, CH_2_CO, and CH_3,_ were also detected. Cu−CH_2_ vibrations (∼391 cm^−1^) were detected for the first time in the low wavenumber region, while Cu−CH_3_ peaks (∼408 cm^−1^) appeared at higher current densities. For the first time, the co‐adsorbed CH_2_ and CO species forming CH_2_CO (1102 cm^−1^) was identified as a key intermediate for C_2+_ product formation. This work not only provides the first direct in situ spectroscopic evidence of CH_2_CO intermediates in a CO_2_RR system but also clarifies the decisive role of interfacial microenvironment in the distribution of multi‐carbon products, laying an important foundation for the design of efficient CO_2_ electro‐reduction catalysts and interfacial structures.

Herzog et al. [106] applied sub‐second time‐resolved in situ surface‐enhanced Raman spectroscopy (SERS) to dynamically track the evolution of the oxidation state of pre‐reduced Cu_2_O nanocubes and key adsorbates (CO_ad_, OH_ad_) on the surface under pulsed potential conditions. They found that the co‐adsorption of CO with Cu−O_ad_ or CuO*_x_*/(OH)_y_ species generated by oxidation under anodic pulses optimizes CO adsorption kinetics and enhances ethanol selectivity. It was also pointed out that the intermittent generation and regeneration of OH sites are key to maintaining high ethanol selectivity, providing an important characterization basis for understanding the mechanism of pulsed CO_2_RR and catalyst design.

The spatial heterogeneity within the catalyst layer also significantly influences catalytic activity and selectivity. Fu et al. [167] utilized an innovative in situ cross‐sectional MEA Raman spectroscopy scanning technique (CS‐Raman MEA Cell) to achieve spatial resolution in situ characterization of the cross‐section of a copper‐based catalyst layer (CL) under real operating conditions (10–200 mA cm^−2^). This technique revealed the spatial heterogeneity of linear CO adsorption (CO_L_; enrichment zone as active region), bridge‐type CO adsorption (CO_B_), and alkaline CuO*_x_*/(OH)_y_ species within the catalyst layer, as well as their dynamic distribution characteristics in response to current density and feed humidity. The catalyst layer was divided into a flooded region (near the AEM), an active region (central region), and an arid region (near the GDL). As the current density increased, the CO_L_ zone was compressed to the middle, and CO_B_ and CuO*_x_*/(OH)_y_ species formed enrichment zones near the AEM and GDL ends, exhibiting an inverted volcanic distribution. Increasing CO_2_ feed humidity expanded the CO_L_ enrichment zone toward the GDL side, improving moisture distribution in the catalytic layer, alleviating flooding and arid zone issues, and enhancing CO_2_RR activity and product selectivity.

Su et al. [168] utilized in situ confocal Raman spectroscopy (CS‐Raman) technology to directly image and analyze reaction species in the CL cross‐section of a GDE in a flow battery with a spatial resolution of ∼4 µm. This method simultaneously identified the spatial distribution of solid copper species (Cu_2_O, CuO*_x_*/(OH)*_y_*), adsorbed intermediates (CO_ad_), and electrolyte ions (e.g., CO_3_^2−^, SO_4_^2−^), revealing chemical composition and local microenvironmental changes within the CL along the thickness direction. In an acidic environment, adjusting the catalyst layer loading or introducing an inert coating (e.g., SiC) can modify the electrolyte penetration depth and the distribution of the active zone, thereby optimizing selectivity and suppressing HER. Another novel approach involves combining in situ Raman spectroscopy (Figure 7e) with DEMS in a MEA to capture the unique key intermediate *CCO at the electrode/polyelectrolyte interface, confirming it as the critical reactant species for C_2_ product formation [169]. This contrasts with the linear adsorption *CO_L_ commonly observed in conventional liquid electrolytes. This phenomenon is attributed to unique structural characteristics of the electrode/polyelectrolyte interface, including higher local pH values, lower moisture content, and stronger hydrogen bonding networks. These factors cause the rate‐determining step of the reaction to shift from the traditional C−C coupling to the hydrogenation reaction of *CCO, favouring the Eley‐Rideal‐type hydrogenation mechanism, thereby tuning CO_2_RR pathway and intermediate transformations.

The local pH, humidity, moisture content, and electrolyte composition in the reaction microenvironment directly regulate the activity and selectivity of catalytic processes. To address this, a lateral surface‐enhanced Raman spectroscopy (L‐SERS) imaging system was successfully developed, enabling high spatial and temporal resolution monitoring of the local pH in the Nernst layer on the catalyst surface [170]. This system avoided substrate interference in SERS, demonstrating that local pH regulation is essential for optimizing CO_2_ reduction performance. Additionally, DFT‐assisted in situ electrochemical shell‐isolated nanoparticle‐enhanced Raman spectroscopy (SHINERS) (Figure 7f) was employed to investigate potential‐dependent evolution of surface adsorbate species during electrochemical reduction of CO_2_ on polycrystalline Cu electrodes [171]. As the potential decreases, surface species undergo a four‐stage evolution: Cu_2_O reduction → competitive adsorption with *COO^−^ → *CO_3_^2−^ generation accompanied by *CO formation → deep reduction of CO. At low overpotentials, carbonate anions hinder CO_2_ activation by competitively occupying active sites. Therefore, adjusting the electrolyte composition and microenvironment can improve catalytic performance.

In summary, in situ Raman spectroscopy, by dynamically monitoring key intermediates, imaging the spatial distribution of the catalyst layer, and systematically revealing the regulation of the reaction microenvironment, has significantly enriched our understanding of the mechanism of the CO_2_ electro‐reduction reaction, providing a scientific basis and technical support for the design of efficient catalysts and devices.

##### 2.2.3.3 In Situ Nuclear Magnetic Resonance (NMR) and Ultraviolet‐Visible Spectroscopy (UV‐Vis)

Liquid‐phase NMR, especially in situ liquid‐phase NMR, can analyze the concentration evolution of liquid‐phase products (e.g., HCOO^−^, CH_3_COO^−^, etc.) and capture characteristic signals of short‐lived intermediates in some systems. Combined with isotope labeling strategies, NMR can also distinguish the C sources of products and reaction pathways, thereby providing multidimensional information support for reaction selectivity analysis [173]. UV‐Vis focuses on monitoring charge transfer behavior and electronic structure changes in metal centers [174]. When used in conjunction with XAS, it can supplement dynamic information on metal valence state changes and coordination environment restructuring, providing corroborative evidence for the construction of electronic structure regulation mechanisms during reactions [174].

To address signal instability caused by magnetic field perturbations in gas–liquid–solid three‐phase reaction systems, Xu et al. [172] developed an in situ electrochemical NMR microreactor tailored for three‐phase reactions. This enabled real‐time monitoring and quantitative analysis of hydrogen, carbon, and oxygen trajectories in the CO_2_RR without the need for product separation. This method captured oxygen from water molecules directly participating in HCOO^−^ generation (*COOH regeneration pathway) through a water‐assisted mechanism in Cu and its bimetallic Cu‐based materials (Bi_2_CuO_4_ and In_2_Cu_2_O_5_) (Figure 7g), improving HCOOH selectivity (34.2% → 98%). They clearly confirmed via ^17^O NMR that oxygen from water molecules directly enters the molecular structure of formic acid, with the content of C═^17^O in the product increasing linearly over time. Additionally, they revealed the rapid reversible exchange of CO_2_, H_2_O, and HCO_3_^−^, as well as the formation of an interfacial hydrogen bonding network, providing support for multi‐element trajectory tracking and mechanism elucidation. However, the testing conditions for this method were primarily at lower current densities, still differing from industrial conditions. To address the lack of characterization under high current conditions, Zhu et al. [175] developed an in situ technique combining a gas diffusion flow cell with a benchtop NMR, enabling real‐time multi‐parameter online monitoring of eCO_2_RR at 100–200 mA cm^−2^. ^1^H NMR quantitative analysis showed that under high current conditions, the FE of formic acid and ethanol decreased from 12.13% to 6.01% and from 8.64% to 1.27%, respectively, accompanied by a pH decrease from 14 to 8 and a rise in bicarbonate concentration to 2.8 M. It was also found that water migration rates increased at high currents, dominated by electromigration mechanisms, leading to underestimation of product concentrations and battery failure. This method overcomes the low‐current limitation of traditional in situ techniques, providing a new approach for analyzing reaction environments under industrial conditions.

The aforementioned work primarily focuses on the overall product distribution and changes in the reaction environment. However, to directly reveal the synergistic effects of active sites and the specific role of water molecules in the reaction pathway, a more refined source tracing strategy is required [176]. For example, Zhou et al. [176] used ^17^O isotope labeling combined with in situ NMR to directly monitor the participation of oxygen from water molecules in the formation of formate in the SnCu‐WC*_x_* catalyst system. Using in situ ATR‐SEIRAS, a *COOH intermediate peak at 1240 cm^−1^ was detected, confirming that *OH generated from water splitting at Sn sites couples with *CO adsorbed at Cu sites, accelerating the conversion of *CO to *COOH, enabling the catalyst to achieve a FE_formate_ of 98.62% at 100 mA cm^−2^ and maintain >90% even at 500 mA cm^−2^.

Although NMR has significant advantages in source tracing, spectroscopic techniques are more suitable for identifying transient electronic structure changes and radical intermediates. In situ UV–Vis demonstrated unique value in this regard. In SnCu_1.5_O_3.5_@MFI system [177], the 220 nm absorption peak corresponded to the formation of CO_2_•^−^ intermediate, with intensity increasing as potential decreased, peaking at −1.55 V_RHE_. According to the Beer–Lambert law, the intermediate concentration is highest at this potential, providing more protonated raw materials for CO_2_RR and effectively enhancing the reduction efficiency of CO_2_ to methane. This ability to capture intermediate peaks also provides a possibility for studying the influence of external physical fields on the reaction pathway. Furthermore, Deng et al. [178] revealed the influence of magnetic fields on the enhanced selectivity of non‐ferromagnetic bismuth‐based catalysts (including bismuth metal–organic frameworks (Bi‐MOFs) and bismuth‐based single‐atom catalysts (Bi‐SACs)) toward surface radical intermediates by combining in situ UV–Vis with externally applied magnetic fields. Upon applying a 0.9 T magnetic field, the OCHO* peak of the Bi‐MOF electrode showed a remarkable enhancement, confirming that the magnetic field promotes accumulation and stabilization of OCHO* radicals. At this point, the FE_formate_ at −1.2 V increased to 98.3%, with a 63.2% increase in current density. On the Bi‐SACs catalyst, the *COOH peak showed no significant change under a magnetic field. These results reveal that the magnetic field selectively promotes the accumulation and stabilization of certain radicals through spin regulation, thereby steering reaction intermediate pathways and product distribution. This provides direct experimental evidence for using physical fields to optimize CO_2_RR selectivity.

##### 2.2.3.4 Multimodal In Situ Characterization

Multimodal in situ characterization enables the simultaneous capture of changes in structure, morphology, chemical state, and reaction environment, thereby reconstructing the true evolution process of catalysts within a unified spatiotemporal framework. This advantage is not only applicable to revealing activation mechanisms at short time scales but can also be extended to durability studies under long‐term operation and industrial conditions.

**FIGURE 8:**
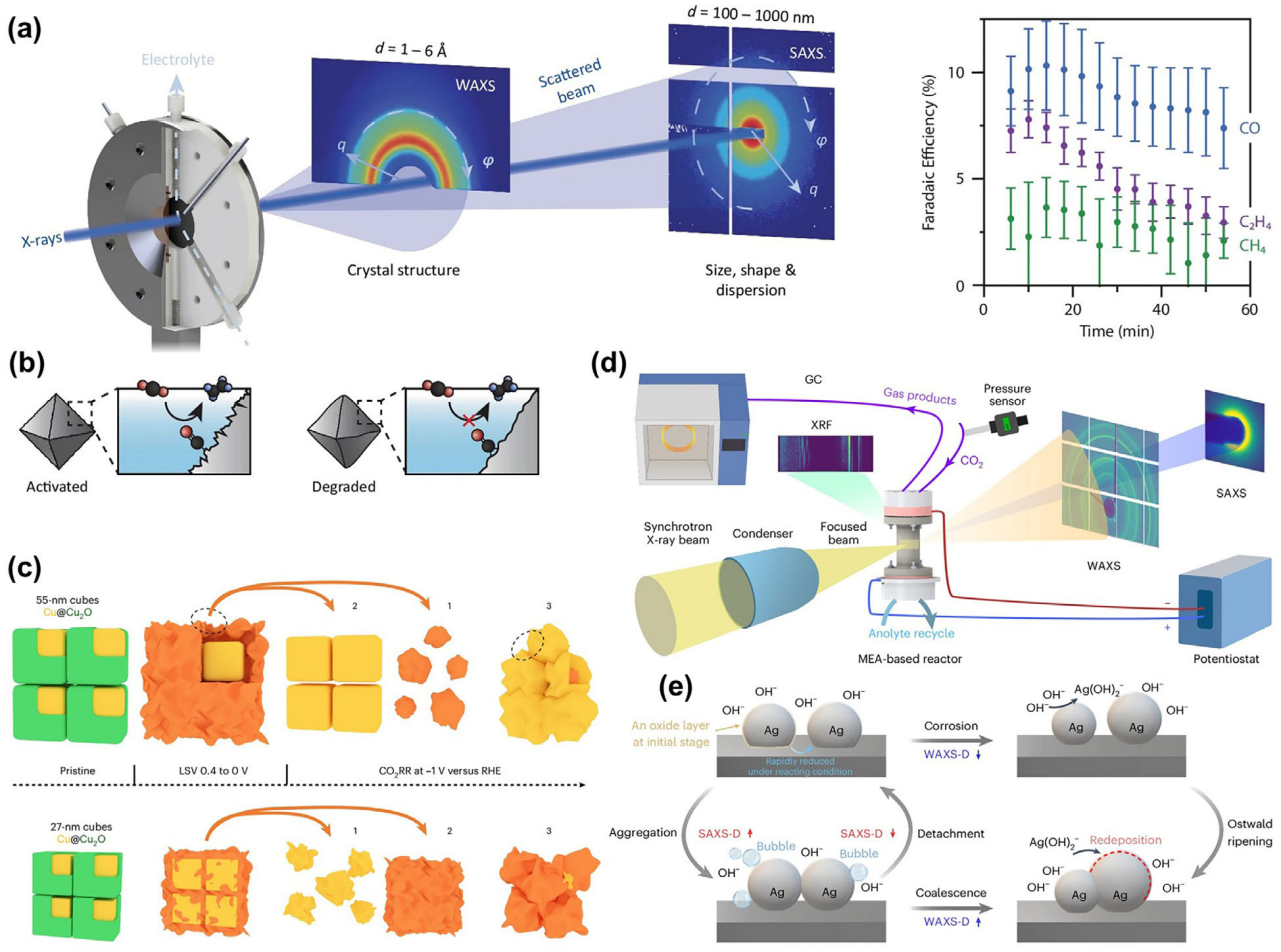
(a) Illustration of an electrochemical cell for in situ X‐ray scattering experiments and FE of the three main gas products within 1 h; (b) Schematic of structural changes. Reproduced with permission [179]. Copyright 2025, The Author(s). (c) Schematic of Cu carbonyl‐driven restructuring of 55 nm Cu@Cu_2_O nanocubes, and 27 nm nanocubes reconstruction. Reproduced with permission [180]. Copyright 2025, The Author(s), under exclusive licence to Springer Nature Limited. (d) Illustration of in situ X‐ray characterization device used for CO_2_ electrolysis measurements; (e) Graphical representation of the structural evolution mechanism of Ag nanoparticles in stability testing. Reproduced with permission [181]. Copyright 2025, The Author(s), under exclusive licence to Springer Nature Limited.

In the analysis of short‐term dynamic processes, Stam et al. [179] combined millisecond–second resolution multiscale in situ SAXS/WAXS with in situ Raman spectroscopy to systematically depict the entire process from activation to deactivation of Cu_2_O octahedra and cubes in CO_2_RR. WAXS revealed that Cu_2_O completed the transformation from oxide to metallic Cu within ∼40 s, accompanied by lattice contraction and grain size reduction; SAXS further showed a decrease in particle size, increased polydispersity, and retention of the original octahedral morphology. Raman spectroscopy revealed that step sites generate single *CO bonds during the initial activation phase, which facilitates the formation of C─C coupling products. However, after prolonged operation, surface roughening and the accumulation of bridged *CO lead to a decrease in C_2_ product selectivity and an increase in H_2_ ratio (Figure 8a,b). These results provide a kinetic reference for subsequent higher‐dimensional multi‐scale structure–performance correlation studies. Based on this insight into time‐resolved dynamics, Yang et al. [180] further extended their study to a comprehensive analysis across scales and with multi‐signal cross‐validation, integrating in situ electrochemical liquid‐phase STEM (EC‐STEM), four‐dimensional STEM diffraction imaging (4D‐STEM), high‐energy‐resolution fluorescence‐detected X‐ray absorption spectroscopy (HERFD‐XAS), in situ Raman spectroscopy, and DFT calculations, enabling a comprehensive tracking of the morphological, crystallinity, and valence state evolution of Cu@Cu_2_O nanocubes under CO_2_RR conditions (Figure 8c). EC‐STEM captured the potential‐dependent evolution pathways of particles with different sizes and the formation of single‐atom Cu−CO clusters; 4D‐STEM distinguished the proportional differences between crystalline and amorphous regions; HERFD‐XAS quantitatively analyzed the valence state changes during the reduction‐reoxidation process; Raman and DFT revealed the mechanism of CO‐driven single‐atom Cu migration. This multidimensional closed‐loop analysis significantly improved the resolution of the structure–performance relationship and provided a methodological foundation for extending the findings to industrial conditions. When multimodal techniques are introduced into operating systems under conditions close to industrialization, their value becomes even more evident. Xu et al. [181] used an in situ synchrotron X‐ray multimodal platform (WAXS/SAXS/XRF), combined with a high‐energy focused X‐ray beam and multiple detectors, to achieve simultaneous monitoring of ion migration, water movement, crystal structure changes, and catalyst morphology evolution within MEAs (Figure 8d). In zero‐gap MEAs, gold catalysts maintain structural stability during accelerated stress testing (AST) due to their phase stability and strong adhesion, while silver catalysts exhibit particle agglomeration, dissolution‐recrystallization, and desorption (Figure 8e). Comparing constant current and pulsed AST, the platform effectively distinguished different degradation pathways and validated the role of pulsed strategies in suppressing salt crystallization and accelerating durability evaluation, advancing the establishment of a degradation mechanism characterization system under industrial conditions.

From short‐time‐scale nanostructure dynamics to cross‐scale multimodal cross‐validation, and finally to durability evaluation under industrial conditions, these three studies collectively outline the technical evolution and application expansion path of multimodal in situ characterization in the study of electrocatalyst dynamic evolution. It is worth emphasizing that a single technique often fails to provide a complete three‐dimensional mapping of the structure‐electron‐reaction pathway, making multi‐modal coupled characterization the consensus trend in current top‐tier research. For example, combining in situ XAS with SEIRAS can simultaneously resolve electronic state reconstruction and intermediate evolution, while further integrating XRD or TEM enables concurrent tracking of crystal structure changes. By constructing a “structure‐electronic‐reaction pathway” co‐evolutionary map, this approach not only helps identify the true active state rather than the initial structure (precatalyst) but also provides a robust empirical foundation for subsequent theoretical modelling and reverse design.

Of course, the design of in situ characterization cells also deserves consideration. Under engineering‐relevant conditions (i.e., high current densities > 200 mA cm^−2^, GDE/MEA configurations, strong local alkalization of electrolyzers, and thick porous electrode structures), the actual reaction environment at the catalytic interface is different from traditional laboratory settings. As a result, the design of the in situ cell not only affects the quality of the spectroscopic signals, but also determines whether the obtained data can truly reflect the working‐state structure and interfacial chemistry of the catalyst.

The major technical challenges of performing operando/quasi‐operando characterization under these operational conditions are as follows: (i) Limited signal accessibility. The hundreds of micrometers‐thick carbon paper, porous layers, and ion‐exchange membranes in GDE/MEA cause attenuation of X‐ray, electron beam, and optical signals, hindering direct detection of interfacial reaction fronts (typically only 10−200 nm). (ii) Highly non‐uniform local environment. High‐flux CO_2_ mass transfer, strong local pH gradients, and dynamic three‐phase interface reconstruction shorten the lifetime of reaction intermediates to milliseconds or even sub‐millisecond level, which cannot be trapped by conventional time‐resolved characterization techniques. (iii) Compatibility limitations of window materials with cells. At ampere‐level current densities, window materials may suffer mechanical instability owing to localized heating or bubble disturbance, while high‐resistance regions within the MEA structure can trigger severe Joule heating, limiting the continuous operating time of operando devices. (iv) Enhanced interference signals. Components such as the carbon support, binder, PTFE, and electrolyte additives could produce strong background signals or peak overlap in Raman/IR/XAS measurements, complicating the identification of interfacial intermediates.

For addressing the challenges mentioned above, several types of specialized operando cells design tailored for engineered electrolytic environments have been developed in recent years. It provides important supplementary insights into the structural evolution, electronic states/coordination structures, and interfacial intermediate dynamics: (i) Grazing‐incidence X‐ray absorption spectroscopy/diffraction (GI‐XAS/GI‐XRD) [182, 183, 184]. By controlling the incident angle, these techniques achieve depth resolution from the nanometer to micrometer scale. They enable selective probing of only the 10−200 nm reaction‐front region inside thick GDE/MEA, making them suitable for operando analysis of structural evolution. (ii) Ultrafast transient Raman/SERS‐flow cells [185, 186]. These systems can capture short‐lived intermediates with time resolutions of 10^−6^−10^−3^ s, overcoming the temporal limitations of conventional spectroscopy and providing direct evidence for interfacial intermediate tracking. (iii) Membrane‐compatible operando XAS (three‐compartment MEA‐XAS cells) [182, 187]. These allow stable acquisition of edge features and coordination‐number changes under 0.5−1.0 A cm^−2^, enabling investigation of electronic and coordination structures at industrial current densities. (iv) X‐ray transparent GDEs (CNT‐membrane/graphene windows) [188, 189]. Such designs enhance signal penetration and reduce background, allowing recording of nanoscale reconstruction, phase transitions, and oxidation‐state changes under industrial conditions. (v) AI‐assisted spectral deconvolution. It is used to accurately extract transient intermediates (e.g., *OCCO, *CHOCO, *OC−CHO) under conditions of strong background and multi‐peak overlap, improving the interpretability of operando data in engineering systems. Beyond this, general principles like optimizing optical paths, adjusting thin‐layer structures, ensuring electrode mechanical stability, managing mass transfer, controlling bubble release, and refining temperature and sealing designs remain equally crucial for all in situ techniques.

Summary, these emerging *operando* tools not only fill the observational gaps in structure, electronic state, and interfacial chemistry at engineered conditions but also correspond directly to three characterization objectives of Sections 2.2.1, 2.2.2, and 2.2.3. They enable mechanistic insights developed under laboratory conditions to be extended to real application scenarios.

## 3 Microenvironment Regulation

**FIGURE 9:**
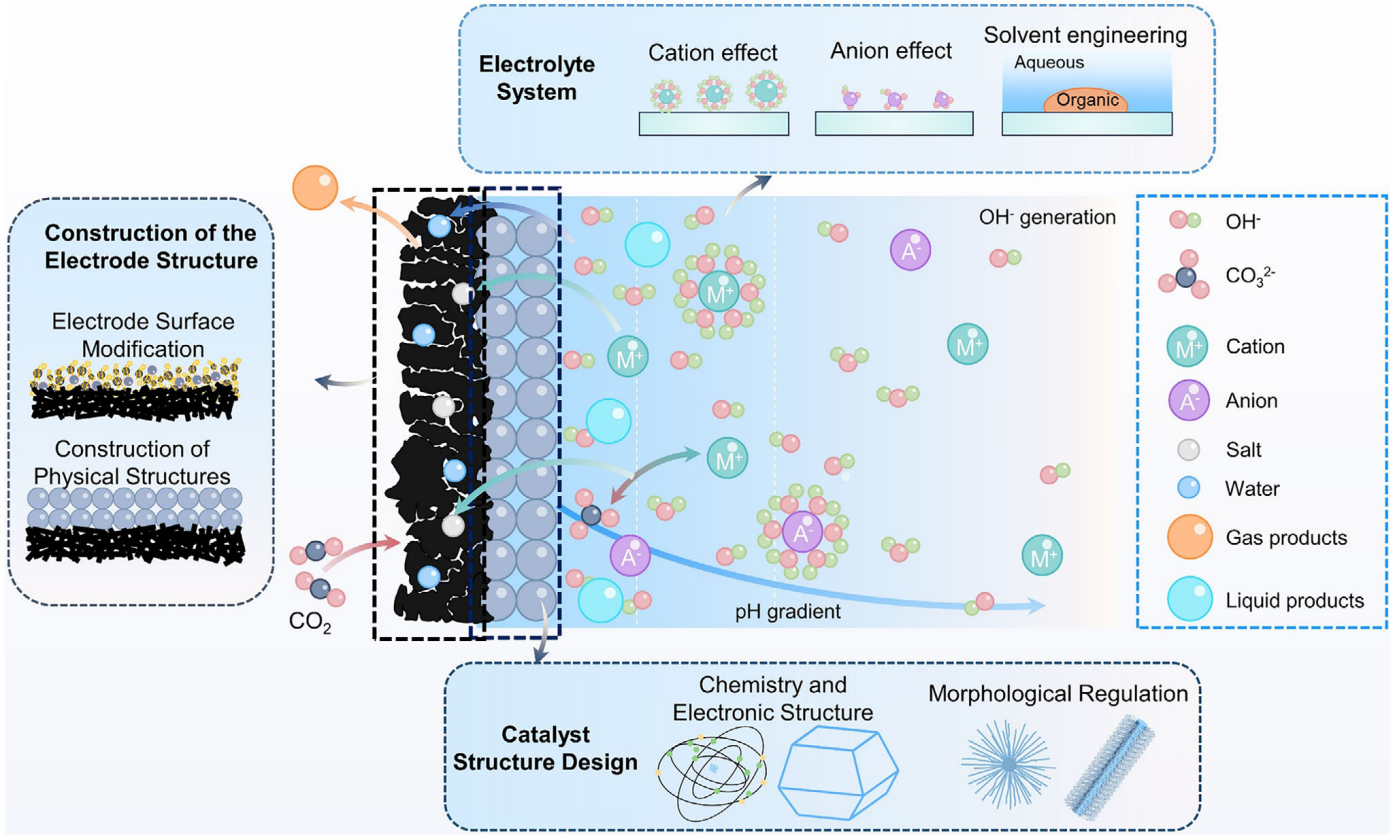
Schematic diagram of the microenvironment, including catalyst design, construction of electrode structure, and electrolyte regulation.

With the continuous deepening of understanding of the intrinsic properties of base catalysts in the eCO_2_RR mechanism, researchers have reached a clear consensus: although the structural characteristics and electronic states of catalysts determine their potential reaction capacity, another key factor that dominates their reaction activity, product selectivity, and operational stability lies in the dynamic microenvironment (dynamic interfacial microenvironment) constructed during the reaction process. The so‐called microenvironment refers to the reaction space formed by various physicochemical parameters within the sub‐nanometer to micrometer scale range at the catalyst surface or interface, including but not limited to the local concentration of reactants, electric field strength and distribution, proton and electron migration pathways, hydration structure arrangement, and electrolyte composition and ion behavior (Figure 9). Microenvironment regulation not only acts on the catalyst‐reactant interface itself but also extends to multiple processes such as electrolyte ion distribution, electric field gradients, local pH, and intermediate transport and conversion, thereby dynamically constraining or activating reaction pathways through multi‐faceted interactions. Unlike traditional catalysis, which focuses solely on the “intrinsic optimization” of active sites, the high selectivity and industrial feasibility required for eCO_2_RR necessitate the integration of “extrinsic regulation” with “structure–interface–environment” synergistic strategies. Recent investigations have demonstrated that, even without altering the catalyst material itself, it is possible to significantly improve the selectivity of C_2+_ products, reduce the overpotential required for the reaction, and extend the stable lifetime of the catalyst under high current density conditions by exploiting microenvironment engineering [190, 191]. Therefore, microenvironment regulation is rapidly gaining strategic importance and is now considered to be as important as the structure–property design of the catalyst itself. This section focuses on catalysts and systematically discusses three core aspects: catalyst structure construction, electrode structure, and electrolyte system design.

### 3.1 Catalyst‐Driven Microenvironment Self‐Regulation

**FIGURE 10:**
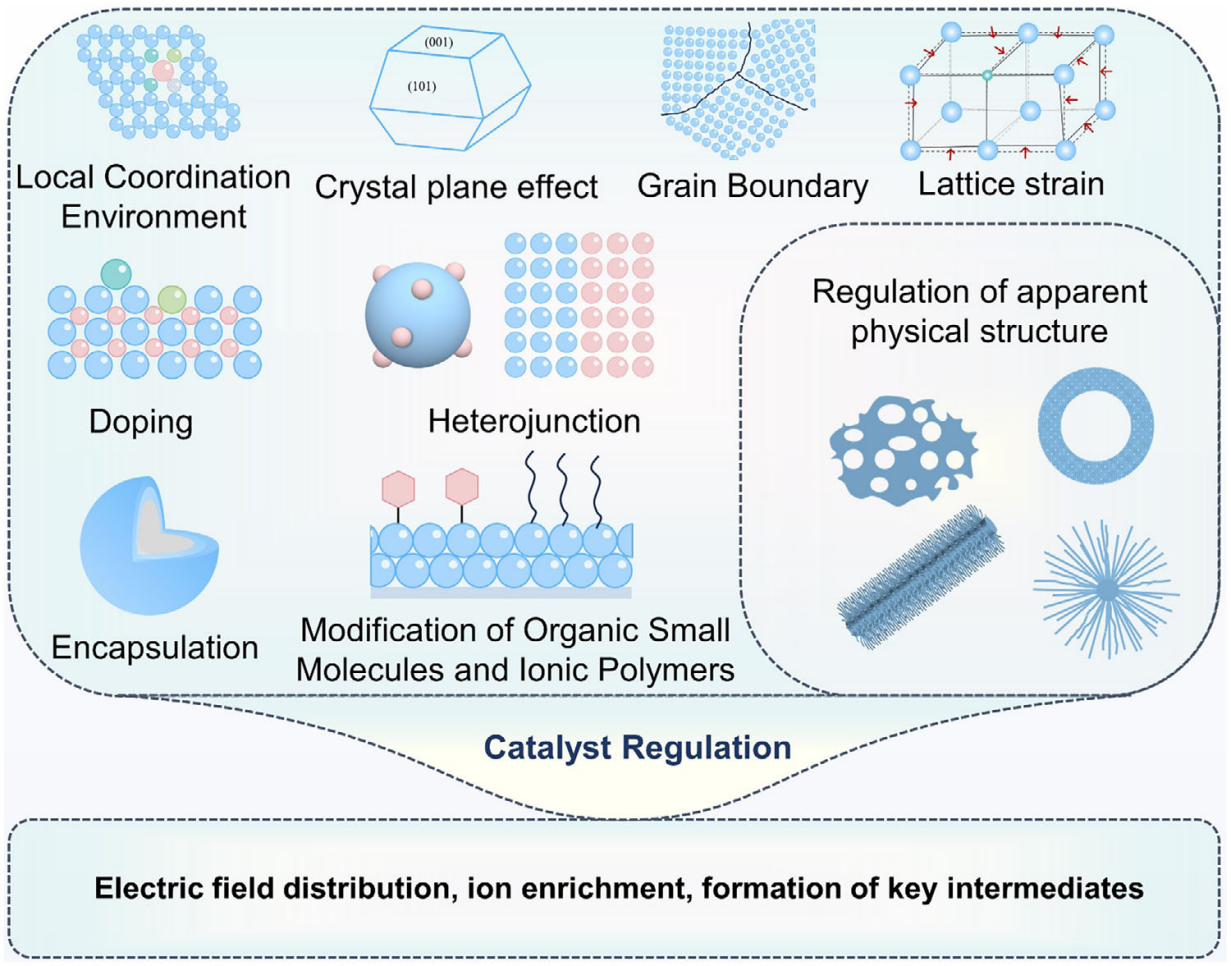
Schematic diagram of catalyst‐driven microenvironment self‐regulation via regulating catalyst structure.

In eCO_2_RR research, catalyst structure regulation has become one of the most mature and widely adopted research directions. The structure of a catalyst not only determines its intrinsic electronic state and adsorption behavior, but also dynamically shapes the local interface microenvironment under electrolytic conditions, such as the distribution of the interfacial electric field, ion enrichment behavior, and the generation and transformation trajectory of key intermediates. As such, regulatory strategies can be developed at the atomic scale by optimizing electronic structures to reconfigure adsorption energy barriers and reaction pathways, or extended to higher‐dimensional nano/mesoscale levels by employing morphology engineering to regulate mass transport and interfacial spatial effects (Figure 10). These approaches are not mutually exclusive but rather interact synergistically at the interfacial microenvironment to jointly determine reaction efficiency and selectivity. Therefore, multi‐scale regulation from electronic structure to morphology is not only an “active center engineering” for adjusting reaction energy barriers but also a “microenvironment engineering” targeting interface conditions, aiming to achieve precise regulation of CO_2_RR through layer‐by‐layer optimization of the interface environment. Next, we will discuss the understanding and regulation of the microenvironment, starting from the electronic structure and morphology of catalysts.

#### 3.1.1 Regulation of Chemical and Electronic Structures

**FIGURE 11:**
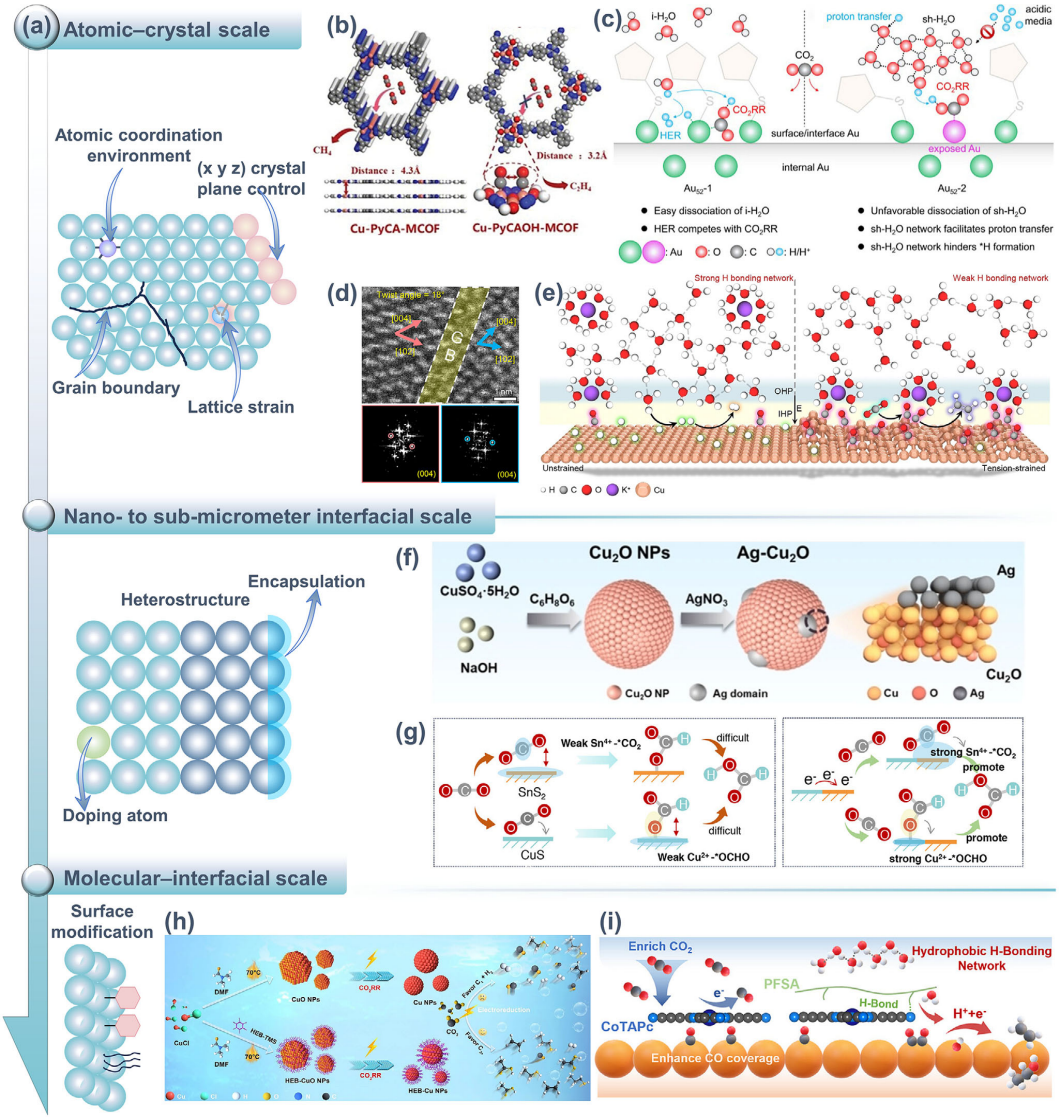
(a) Regulation of catalyst chemistry and electronic structure. (b) Structural models of Cu‐PyCA‐MCOF and Cu‐PyCAOH‐MCOF. Reproduced with permission [204]. Copyright 2024, Wiley‐VCH GmbH. (c) Electrocatalytic pathways of Au_52_‐1 and Au_52_‐2 under different conditions. Reproduced with permission [205]. Copyright 2025, Wiley‐VCH GmbH. (d) AC‐STEM image of GB‐SnS nanosheets at grain boundaries with FFT pattern. Reproduced with permission [206]. Copyright 2025, Wiley‐VCH GmbH. (e) Schematic of K^+^ accumulation by tension‐strained Cu. Reproduced with permission [207]. Copyright 2025, Wiley‐VCH GmbH. (f) Schematic prepared for Ag‐Cu_2_O‐X. Reproduced with permission [211]. Copyright 2024, Wiley‐VCH GmbH. (g) Illustration of CuS, SnS_2_ (left), and CuS/SnS_2_ (right) to produce formic acid. Reproduced with permission [213]. Copyright 2025, Wiley‐VCH GmbH. (h) Schematic of HEB‐CuO and CuO NPs synthesis. Reproduced with permission [214]. Copyright 2024, The Author(s). (i) Schematic of the design and interface regulation strategy of CoTAPc/Cu. Reproduced with permission [215]. Copyright 2025, Wiley‐VCH GmbH.

Electronic structure is the “internal factor” that determines the active sites and reaction pathways of catalysts. By modifying atomic‐local properties (such as coordination) [192, 193], defect engineering (regulating crystal lattice/interfaces, doping, etc.) [194, 195, 196], and composite and surface modifications [197, 198, 199, 200, 201, 202], it is possible to adjust adsorption energy barriers and reaction pathways at the atomic or molecular scale (Figure 11a).

In terms of atomic coordination environment regulation, Zhao et al. [203] prepared a series of single‐atom catalysts (Ni_1_−, Mn_1_−, Fe_1_−, Co_1_−, Cu_1_−NC) with M−N_3_ coordination microenvironments using the N/O mixed pre‐coordination pyrolysis (NOPP) strategy. Among these, Ni_1_−NC maintained high FE_CO_ at a current density of 300 mA cm^−2^ in a flow cell. This was attributed to the low‐coordination Ni−N_3_ environment, which significantly promotes the formation of *COOH intermediates and accelerates CO_2_ activation. Furthermore, the generated CO was directly utilized in various carbonylation reactions, successfully synthesizing multiple high‐value‐added organic chemicals. In a further strategy to fine‐tune the coordination structure, by regulating the coordination microenvironment of the trinuclear copper cluster in the metal‐covalent organic framework (MCOF), the Cu sites were successfully transformed from an uncoordinated state facing the channel (Cu‐PyCA‐MCOF) to a nanosheet structure coordinated with OH^−^/H_2_O (Cu‐PyCAOH‐MCOF) (Figure 11b) [204]. This modification of the coordination environment not only reduced the *COOH formation energy but also optimized the Cu−Cu spacing (3.2 Å), stabilizing the key intermediate and promoting C−C coupling, thereby achieving the controlled selective conversion of CO_2_ electro‐reduction products from CH_4_ to C_2_H_4_. A 50.5% FE for C_2_H_4_ and a current density of 200.2 mA cm^−2^ were achieved at −1.2 V. This work indicates that the precise design of the micro‐coordination environment can directly regulate the reaction pathway, providing strong support for the selectivity of C_2+_ products.

In addition to the regulation of the coordination environment, crystal facet engineering can also significantly influence the electronic structure and reaction selectivity of catalysts. For example, a ring‐shaped thiol ligand etching strategy was employed to expose Au atoms on the Au(111) crystal plane of Au_52_ nanoclusters, forming a unique surface/interface microenvironment (Figure 11c) [205]. This surface modification not only reduces the activation energy barrier for *COOH formation but also promotes the formation of a dense, strong hydrogen bond network between interfacial H_2_O molecules, optimizing proton transfer and accelerating the protonation of the *COO^−^. This significantly suppresses HER. Au_52_‐2 exhibited high activity and selectivity in alkaline, acidic, and neutral electrolytes, with a high FE_CO_ of 96.9% and a CO partial current density of up to 71.45 mA cm^−2^, while maintaining structural stability under prolonged electrolysis. This work has demonstrated that exposing atomic‐level active sites on crystal faces enables synergistic optimization of the eCO_2_RR through coordinated improvements in electronic structure and local microenvironment, allowing controlled guidance to the desired product.

In the field of grain boundary engineering, SnS nanorings rich in grain boundaries were constructed using solution‐phase cation exchange (CE) technology (Figure 11d) [206]. They exhibited excellent CO_2_RR performance in 1 M KOH: the FE_formate_ reached 98.9% (∼1.7 times higher than SC‐SnS) at −1.0 V_RHE_, and the local current density reached 204.6 mA cm^−2^ at −1.2 V_RHE_, increasing by 3.5 times. In a MEA, it maintained >80% FE for 150 h of continuous stable operation at 200 mA cm^−2^. Analysis revealed that the distribution of twist angles at grain boundaries significantly influences the stabilization of intermediates. Large‐angle grain boundaries reduce the stabilization energy barrier of *OCHO and suppress *H adsorption, thereby enhancing formate selectivity and inhibiting HER, while presenting lower charge transfer impedance. Small‐angle grain boundaries exhibited the opposite trend. This work overturns the traditional view that “all grain boundaries are beneficial” and provides scalable design principles for defect‐ and grain‐boundary‐engineered CO_2_RR catalysts. Moreover, lattice strain has also been used to regulate the microenvironment of the catalyst surface, thereby influencing CO_2_RR pathways. A Cu‐based catalyst derived from the thermal oxidation of a metal‐organic framework (HKUST−1) was reported by introducing different degrees of tensile lattice strain (0%∼4%) by controlling the heating rate [207]. It was found that tensile strain on Cu generates an electron‐rich surface, leading to the accumulation of the electrolyte K^+^ near the catalyst, thereby regulating the electronic state of the catalyst surface and the distribution of water/cations at the interface, which helps activate CO_2_ and suppress the HER (Figure 11e). Ultimately, a high C_2_H_4_ FE of 50.3% (C_2+_ FE of 79.0%) was achieved in an electrolyte with a pH of 1 and low K^+^ concentration. And, in a proton‐exchange membrane (PEM) electrolyzer, CO_2_ was selectively converted to C_2_H_4_ at a current density of 400 mA cm^−2^, with a FE of 44.3%, and the system operated continuously for over 100 h.

In addition to lattice modulation, element doping is also a commonly used approach. To address the issues of reactant concentration and mass transfer, as well as insufficient local *CO intermediate concentrations during the catalytic process, the problem was successfully resolved by doping Y into a porous copper catalyst (Y@CuO*_x_*) [208]. It not only improves the hydrophobicity of the catalyst and effectively suppresses HER, but also enhances the local enrichment of *CO intermediates through the porous structure, optimizes the local basic microenvironment, and promotes C−C coupling reactions. The catalyst achieved a C_2+_ FE of up to 69.19% in a flow cell, fully demonstrating the synergistic effect of doping and pore size regulation. The introduction of single‐atom dispersed tin (Sn, 0.1 wt%) on silver nanoparticles (Ag NP) to construct negatively charged silver active sites (nc‐Ag) could also solve this problem [209]. The negatively charged active sites not only activate CO_2_ through short‐range chemical bonding but also enhance the electric field strength of the electrochemical double layer (EDL), repel interfacial H_2_O, disrupt hydrogen bond networks, enrich local CO_2_, suppress the HER, and ultimately achieve the goal of increasing the CO_2_RR reaction rate. It is worth noting another unique doping method. Ni single atoms and atom clusters were anchored onto fluorocarbon compounds (FC) (Ni/FC), spontaneously introducing highly electronegative F^−^ anions [210]. The F^−^‐induced formation of a positive C^δ+^ center leads to significant inhibition of HER and reduces the CO_2_RR energy barrier. The catalyst attained FE_CO_ of 92.0%, 99.1%, and 98.8% in acidic, neutral, and alkaline electrolytes, respectively, and operated stably for over 3000 h at a high current density of 200 mA cm^−2^, whereas Ni/C without F^−^ primarily produced H_2_. This work provides a clear mechanism and design guidance for the role of anions in regulating electrocatalytic interfaces.

Heterostructure construction further expands the means of controlling the electronic structure and microenvironment of catalyst surfaces. Through electronic coupling between different phases or different metals, novel active centers can be formed at the interface. For example, Ag–Cu_2_O heterointerface catalysts were constructed by in situ growing snowflake‐like Ag nanoregions on the Cu_2_O surface (Figure 11f) [211]. Under neutral flow electrolysis conditions, this heterointerface exhibits notable synergistic effects: The multiphase interface can stabilize part of the Cu^+^ and enrich *CO intermediates at the working potential, while reducing the proton coverage and creating a relatively alkaline interface environment, effectively inhibiting hydrogen evolution and promoting the accumulation of C−C coupling‐related intermediates. On the other hand, the electronic structure regulation at the interface favors the formation of *COOH at Ag sites and accelerates the generation and migration of *CO, while the strong adsorption of *CO at Cu sites reduces the energy barrier for *OCCO formation, synergistically promoting ethylene generation. At 650 mA cm^−2^, a FE of 66% for C_2_H_4_ was achieved with a partial current density of C_2_H_4_ reaching 429.1 mA cm^−2^, and good activity and stability were maintained under both alkaline and acidic conditions, significantly outperforming single‐metal and physical mixture systems. This work achieved high selectivity and high current density output for C_2_ products under neutral conditions by regulating the electronic structure of the bimetallic interface and constructing a local alkaline microenvironment, providing a reference strategy for neutral CO_2_RR interface microenvironment engineering and bimetallic synergistic catalyst design. Similarly, an Ag/Ag_2_S heterointerface was constructed on silver nanowires via a controlled sulfidation strategy and used for the selective production of ethanol. This interface regulates the microenvironment through sulfidation‐induced electronic structure optimization, rearrangement of the interfacial water network, and synergistic regulation of local K^+^ (K•H_2_O) enrichment [212]. Recently, Mott‐Schottky‐type heterointerfaces have also been designed by Zou et al. [213], who constructed a Mott–Schottky‐type CuS/SnS_2_ heterointerface catalyst and proposed a charge redistribution regulation strategy to preferentially electro‐reduce CO_2_ to formic acid under acidic conditions. Through interface structure design (Figure 11g), electrons transfer from CuS to SnS_2_, increasing electron density at Sn sites and enhancing acidity, thereby strengthening CO_2_ adsorption and activation. Concurrently, the d‐band center at Cu sites shifts upward, enhancing stability toward the *OCHO intermediate, achieving dual‐site synergistic activation. In acidic electrolytes, it preserves over 80% formic acid selectivity even at an industrial‐scale current density of 11 A cm^−2^, with partial formic acid current densities as high as −817 mA cm^−2^. The material also demonstrated excellent selectivity and stability under different pH conditions.

Coating strategies primarily regulate the catalytic interface microenvironment by constructing core‐shell structures or surface coatings. Under acidic conditions, an Ag@PPy catalyst with a core‐shell structure was synthesized [216]. The hydrophobicity of polypyrrole (PPy) reduced the contact between water molecules and silver nanoparticles (Ag NPs), thereby inhibiting the HER; simultaneously, the shell protected the catalyst from corrosion, enhancing system stability. COMSOL simulations showed that the locally high pH microenvironment formed on the catalyst surface further facilitates CO_2_ reduction. Ag@PPy‐2 exhibited an FE_CO_ of up to 91.7% and long‐term stability at 300 mA cm^−2^ in acidic electrolytes, as well as superior electrochemical surface area and electron transfer ability compared to bare Ag NPs. Recently, PPy has also been frequently used to encapsulate other catalysts [217]. A conductive polypyrrole‐coated Cu/Cu_2+1_O nanofibers (Cu/Cu_2+1_O@SHNC) prepared using a polyvinylpyrrolidone (PVP) template‐assisted PPy coating achieved selective regulation of CO_2_RR products. By forming a superhydrophobic shell layer using conductive PPy, the hydrogen bonding between interfacial water molecules was reduced, leading to an increased local CO_2_/H_2_O ratio, enhanced *CO coverage, and facilitated C−C coupling between *CO and *CHO intermediates. This reduced the energy barrier for the formation of *CHCHOH (the ethanol pathway), shifting the reaction pathway from ethylene to ethanol. In contrast, the control catalyst with only pyrrole groups, Cu/Cu_2+1_O/NC, exhibited stronger hydrophobicity but stronger hydrogen bonding with water molecules, favoring ethylene formation. These investigations provide important references for the application of interface microenvironment engineering in the regulation of electrocatalytic products.

At the molecular level, organic small‐molecule modification has also become an important method for enhancing product selectivity. For example, HEB‐Cu NPs catalysts (Figure 11h) were obtained by modifying the surface of CuO nanoparticles with the organic molecule hexaethylbenzene (HEB) and followed by in situ electro‐reduction [214]. In a MEA electrolyzer, when the local current density reached 387.6 mA cm^−2^, the C_2+_ FE reached 86.14%, and the system operated continuously and stably for over 50 h at 200 mA cm^−2^. This is attributed to HEB modification, which reduces the coverage of K^+^ coordination water (K•H_2_O), enhances the adsorption and surface coverage of *CO intermediates, and suppresses HER. Consequently, it promotes C−C coupling and significantly optimizes the selectivity and stability of CO_2_RR. Undoubtedly, polymer surface modification has also garnered significant attention, further expanding the scale and functionality of organic molecular strategies. For example, Cu hybrid catalysts modified with polyionic liquids (PIL) (Cu@PIL@Cu and Cu@BPIL@Cu) have achieved the maintenance of a localized alkaline microenvironment and K^+^ enrichment through the semi‐rigid, porous PIL structure and dense cation‐anion network, thereby promoting the C−C coupling reaction at the Cu‐PIL interface [218]. The Cu^0^‐Cu^I^ tandem structure reduces the coupling energy barrier. By adjusting the PIL monomer structure, such as replacing imidazole‐pyridine‐imidazole with imidazole‐bipyridine‐imidazole, the local confinement effect and hydrogenation capacity can be enhanced, thereby altering the distribution and formation pattern of C_2+_ products. At 700 mA cm^−2^ and pH 2, Cu@PIL@Cu achieved a C_2+_ FE of 83.1% and an energy efficiency of 37.6%. Additionally, the synergistic effects of organic small molecules and polymers on the microenvironment have been investigated. Specifically, Huang et al. [215] constructed a hydrogen‐bond‐rich bifunctional catalytic interface (Figure 11i) by co‐assembling cobalt tetramine phthalate (CoTAPc) and perfluorosulfonic acid (PFSA) on a Cu surface, achieving high‐selective CO_2_‐to‐C_2+_ conversion under industrial‐scale current densities. This interface reorganizes interfacial water into spatially constrained clusters through hydrogen bonding interactions between CoTAPc and PFSA, thereby promoting the directed transport of protons toward the *CO intermediate while inhibiting non‐selective proton diffusion. The hydrophobic perfluoroalkyl chains of PFSA enhance CO_2_ enrichment at the catalyst‐electrolyte interface, while CoTAPc accelerates *CO formation through metal‐ligand coordination, synergistically increasing *CO coverage and reducing the C−C coupling energy barrier. Ultimately, the catalyst retained 81% C_2+_ selectivity in a 100 cm^2^ MEA electrolyzer at a current of 30 A. It could operate continuously for more than 550 h at a current density of 200 mA cm^−2^. These results reveal the unique role of surface modification in regulating interfacial water and intermediates.

In summary, starting from a single‐atom coordination environment, researchers have continuously expanded the means of fine‐tuning electronic structures and interfacial microenvironments through lattice/face doping, heterointerfaces, and organic/polymer modifications, thereby achieving systematic optimization of reaction pathways and product selectivity. These strategies not only reveal the intrinsic connection between electronic structure regulation and microenvironment engineering but also provide feasible pathways for the design of next‐generation efficient, stable, and scalable eCO_2_RR catalysts.

#### 3.1.2 Regulation of Apparent Physical Structure of Catalysts

In addition to the regulation of the electronic structure of catalysts, the regulation of morphology at the nanoscale/mesoscale is equally critical for influencing the microenvironment. By rationally designing the pore structure, cavity distribution, and surface geometry of catalysts [219, 220], it is possible to effectively improve mass transfer of reactants, local concentration distribution, and stability of intermediates, thereby enhancing the selectivity and efficiency of eCO_2_RR.

**FIGURE 12:**
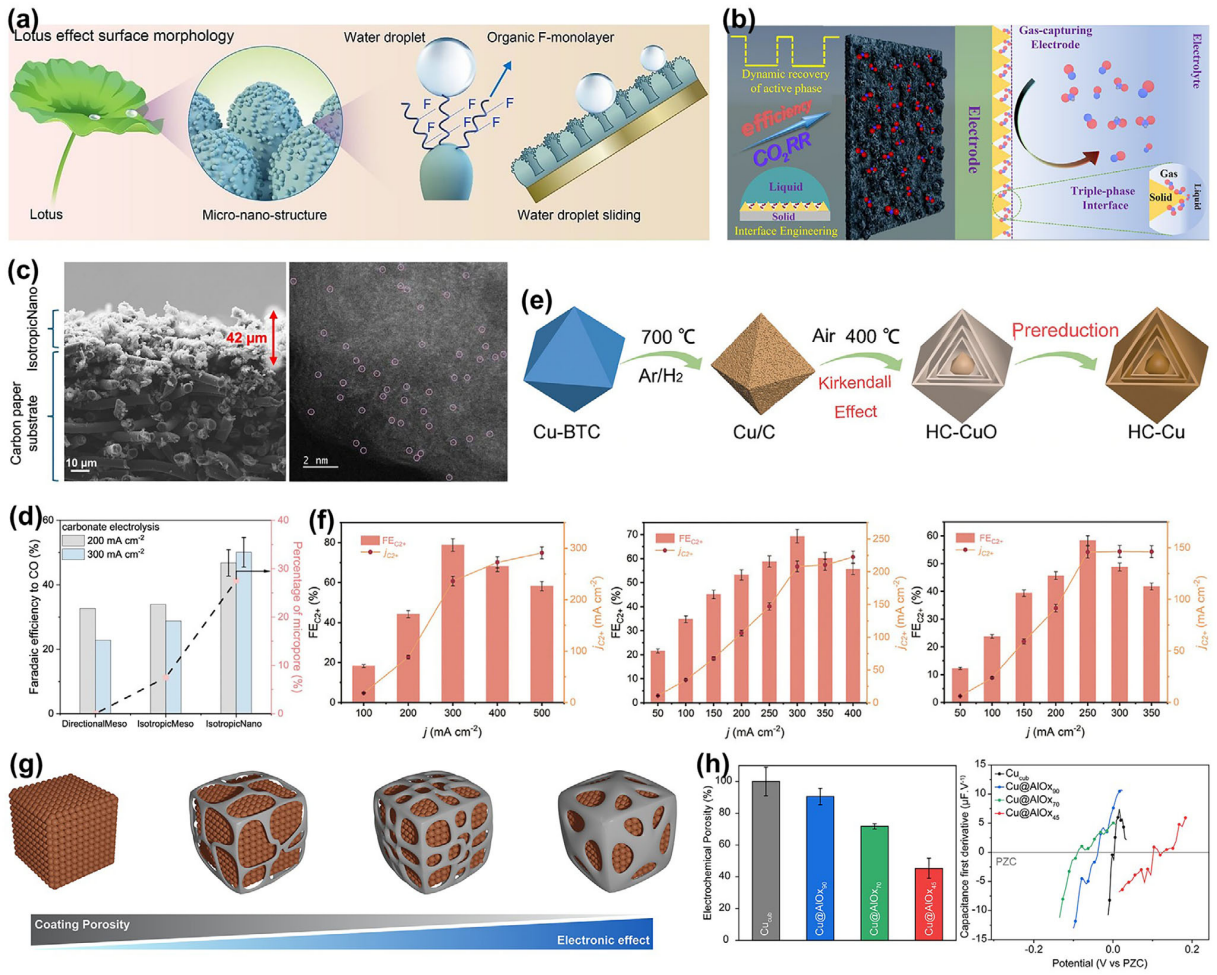
(a) Schematic of “lotus” micronanostructure on SnO_x_ nanoporous film with F‐monolayer and superhydrophobic behavior; (b) Schematic of CO_2_RR mechanism. Reproduced with permission [221]. Copyright 2024, American Chemical Society. (c) Cross‐sectional SEM and HRTEM images of IsotropicNano; (d) Relationship between FE_CO_ and nanopore percentage and electrode. Reproduced with permission [222]. Copyright 2025, The Author(s). (e) Schematic preparation of hierarchical cavity Cu (HC─Cu); (f) FE on Cu‐400 in alkaline, neutral, and acidic electrolytes. Reproduced with permission [223]. Copyright 2025, Wiley‐VCH GmbH. (g) Coating Al_2_O_3_ shells with different porosities on Cu cubes by ALD; (h) Electrochemical porosity and PZC tests. Reproduced with permission [225]. Copyright 2025, The Authors. Published by American Chemical Society.

Basic pore and surface structure regulation can directly improve reactant contact and three‐phase interface performance. A biomimetic “lotus structure” hierarchical nanoporous SnO*_x_* (amorphous SnO + crystalline SnO_2_) catalyst with organofluorine‐containing single‐molecule layers (SAFM) assembled on its surface (Figure 12a,b) was studied by Zhang and colleagues [221]. SAFM forms a superhydrophobic/gas‐loving three‐phase interface and a “gas shield” protective layer, effectively suppressing competitive HER (<5%). The mixture of amorphous and crystalline phases enhances the adsorption capacity of *OCHO intermediates. The synergistic interaction between the nanoporous structure and the hydrophobic fluorine layer increases the local CO_2_ concentration, overcoming the limitation of CO_2_ solubility in aqueous electrolytes, providing favorable conditions for formate and CO generation. Additionally, periodic activation of the catalyst active phase via pulsed square‐wave potential maintained the amorphous/crystalline mixed phase, preventing surface reconstruction and the formation of metallic tin. Ultimately, the catalyst achieved ∼85% stable FE under pulsed conditions and can sustain operation for 10 h, significantly outperforming the approximately 70% efficiency under constant potential conditions. Furthermore, by further optimizing the microenvironment through spatial confinement, the reactant enrichment efficiency can be significantly enhanced. A nickel single‐atom catalyst with a three‐dimensional interconnected nanopore network (IsotropicNano, pore size < 2 nm) was constructed using a template method (Figure 12c), creating a “nano‐confined” space within the catalyst [222]. This effectively creates a high‐local CO_2_ concentration reaction microenvironment in a liquid phase and a low‐reactant environment. This space concentrates in situ generated CO_2_ (i‐CO_2_) through short‐range non‐electrostatic interactions and extends its residence time, thereby increasing the contact probability and activation efficiency of CO_2_ with catalytic active sites. This microenvironment not only optimizes mass transfer processes but also reduces the energy barrier for the formation of key intermediates, accelerating the entire catalytic cycle. At a high current density of 300 mA cm^−2^ (Figure 12d), the catalyst achieved a CO FE of 50%, carbon utilization exceeding 99%, and a total energy efficiency of 46%, while CO_2_ content in the exhaust gas remained below 1%, fully demonstrating the critical role of microenvironment regulation in enhancing macro‐scale performance.

For multi‐electron coupling products, such as C_2+_, morphology regulation can synergistically interact with the optimization of the coordination environment. Therefore, a Cu‐based catalyst with a layered cavity structure and a tunable copper coordination environment was designed using the Kirkendall effect (Figure 12e) [223]. The three‐layer hollow Cu‐400 catalyst significantly enhanced the *CO intermediate coverage (from 0.55 to 5.42 mM) through the spatial confinement effect, and maintained a local OH^−^‐enriched alkaline microenvironment via diffusion hindrance and electrostatic potential gradients, effectively suppressing competitive HER. Simultaneously optimizing the copper coordination number (CN = 9) resulted in moderate *CO adsorption strength, enhancing C−C coupling kinetics while avoiding the adverse effects of overly strong or weak adsorption. Cu‐400 exhibited C_2+_ FE of 78.74, 69.33, and 58.32 in alkaline, neutral, and acidic electrolytes, (Figure 12f) respectively, and maintained over 90% activity for 40 h under acidic conditions, representing an ∼1.87‐fold improvement over traditional solid catalysts. However, the challenge of HER competition persists in acidic systems. The “structure + electronics” dual‐path strategy is undoubtedly an effective solution. For example, by combining one‐step reduction with vacuum freeze‐drying, a porous CuIn bimetallic aerogel (Cu_24_−In−AG) was prepared [224]. Its 3D porous network effectively suppressed the outward diffusion of OH^−^ from the cathode, creating a stable local alkaline microenvironment on the catalyst surface to hinder HER. The introduction of In optimized the electronic structure of the Cu surface, reducing the activation energy barrier for the key intermediate *CO and favoring CO production. At pH 2 acidic MEA conditions, Cu_24_−In−AG achieved 98.3% CO selectivity at 150 mA cm^−2^, and obtained 86% single‐pass carbon efficiency at a gas flow rate of 1 sccm, all significantly superior to the unmodified catalysts. This demonstrates that the synergistic effects of morphology and electronic effects can significantly enhance microenvironment control capabilities.

The highly synergistic interaction between geometric configuration and electronic effects, coupled with an indispensable design strategy. Using a Cu cube as the model system, an Al_2_O_3_ shell layer was introduced via atomic layer deposition (ALD) to synthesize a series of Cu@Al_2_O_3_ catalysts with tunable porosity (Figure 12g), achieving synergistic regulation of geometric and electronic effects [225]. The (100) crystal plane of the Cu cube is a typical selective crystal plane for ethylene products, while the inert Al_2_O_3_ shell induces electronic state restructuring on the Cu surface at the interface. As the shell porosity decreases (90%, 70%, and 45%), the proportion of the Cu‐Al_2_O_3_ interface increases, and the surface potential of zero charge (PZC) shifts significantly, indicating that the local electron density has been regulated and that the electronic effect gradually strengthens (Figure 12h). This system exhibited a systematic transformation in product selectivity: ethylene was the main product in samples with high porosity, while the ratio of methane to H_2_ significantly increased in samples with low porosity. This study demonstrated that interfacial electronic effects not only alter adsorption behavior but also amplify the influence of external microenvironment on intermediate stability, achieving a closed‐loop process from geometric design to electronic regulation to reaction pathway selection.

The above latest research demonstrates a progressive logic from fundamental pore regulation, spatial confinement microenvironment optimization, to coordination and electronic synergy strategies. Morphology design in CO_2_RR not only enhances surface area but also precisely regulates local concentration, reaction pathways, and product selectivity. Single structural or electronic regulation often fails to simultaneously meet the requirements of high selectivity and industrial current density. Multi‐scale, multi‐factor synergistic design has become the key path to enhancing CO_2_RR performance. Future work could further explore the correlation between interface microenvironments and catalyst electronic structures, achieve directed control of multi‐electron reaction intermediates, and advance experimental studies toward industrial feasibility verification, providing systematic design guidelines for efficient and sustainable CO_2_ electrochemical conversion.

### 3.2 Electrode‐Driven Microenvironment Regulation

**FIGURE 13:**
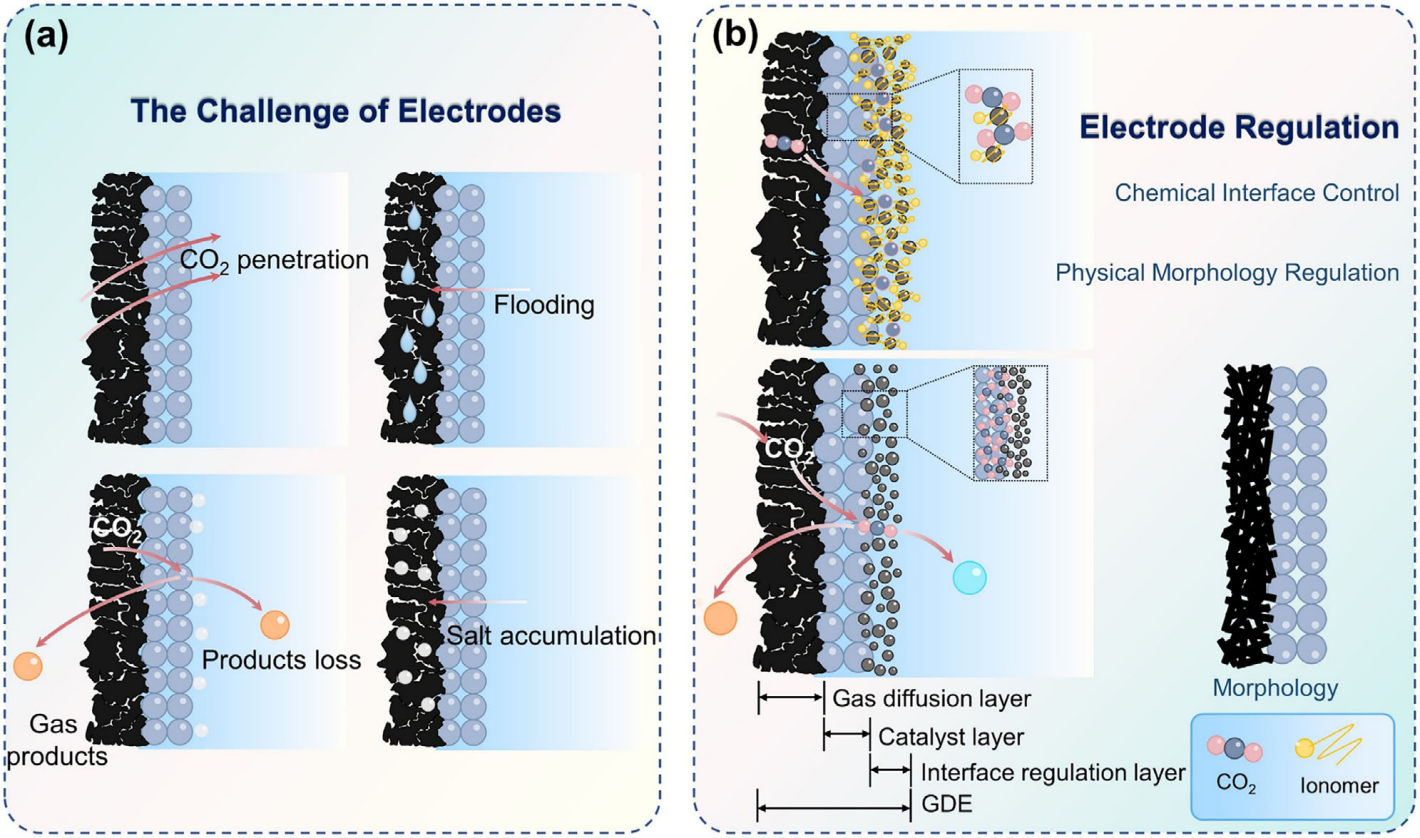
Schematic diagram of (a) the electrode‐level challenge, and (b) electrode‐driven microenvironment regulation via chemical interface and physical structure regulation.

In typical eCO_2_RR processes, reactions primarily occur at the gas–liquid–solid triple‐phase boundary (TPB). The construction of a stable, continuous, and controllable TPB significantly influences reaction rate, selectivity, and product distribution (Figure 13). Triple‐phase boundary regulation not only involves the design of gas diffusion channels but also requires precise adjustment of factors such as hydrophobic/hydrophilic gradient construction, electrolyte wetting behavior, and bubble desorption kinetics. An efficient interface structure must simultaneously meet the following requirements: (i) rapid CO_2_ transport and enrichment capability; (ii) electronic/proton co‐transport pathways; (iii) smooth desorption of intermediates and product discharge, thereby reducing reaction resistance and enhancing Faraday efficiency. To systematically understand electrode interface regulation strategies, they can be categorized into chemical interface regulation and apparent physical structure regulation.

#### 3.2.1 Chemical Interface Regulation

Chemical interface regulation primarily involves modifying the electrode surface properties at the molecular or atomic level to precisely control the adsorption energy of intermediates, reaction selectivity, and local mass supply. In typical gas diffusion electrodes (GDEs), although CO_2_ mass transfer is enhanced, issues such as electrolyte flooding, gas–liquid interface collapse, carbon support degradation, and catalyst deactivation still arise under high current density conditions. To address these challenges, researchers have proposed various chemical interface regulation strategies tailored to different mechanisms: hydrophobic and electronic regulation at the molecular/atomic level [226, 227]; ion‐polymer microenvironment regulation [228, 229]; and pore/three‐phase interface microenvironment regulation [230, 231]. These strategies aim to stabilize the three‐phase interface, optimize intermediate adsorption, and enhance electron/proton transport through the design of the microenvironment.

**FIGURE 14:**
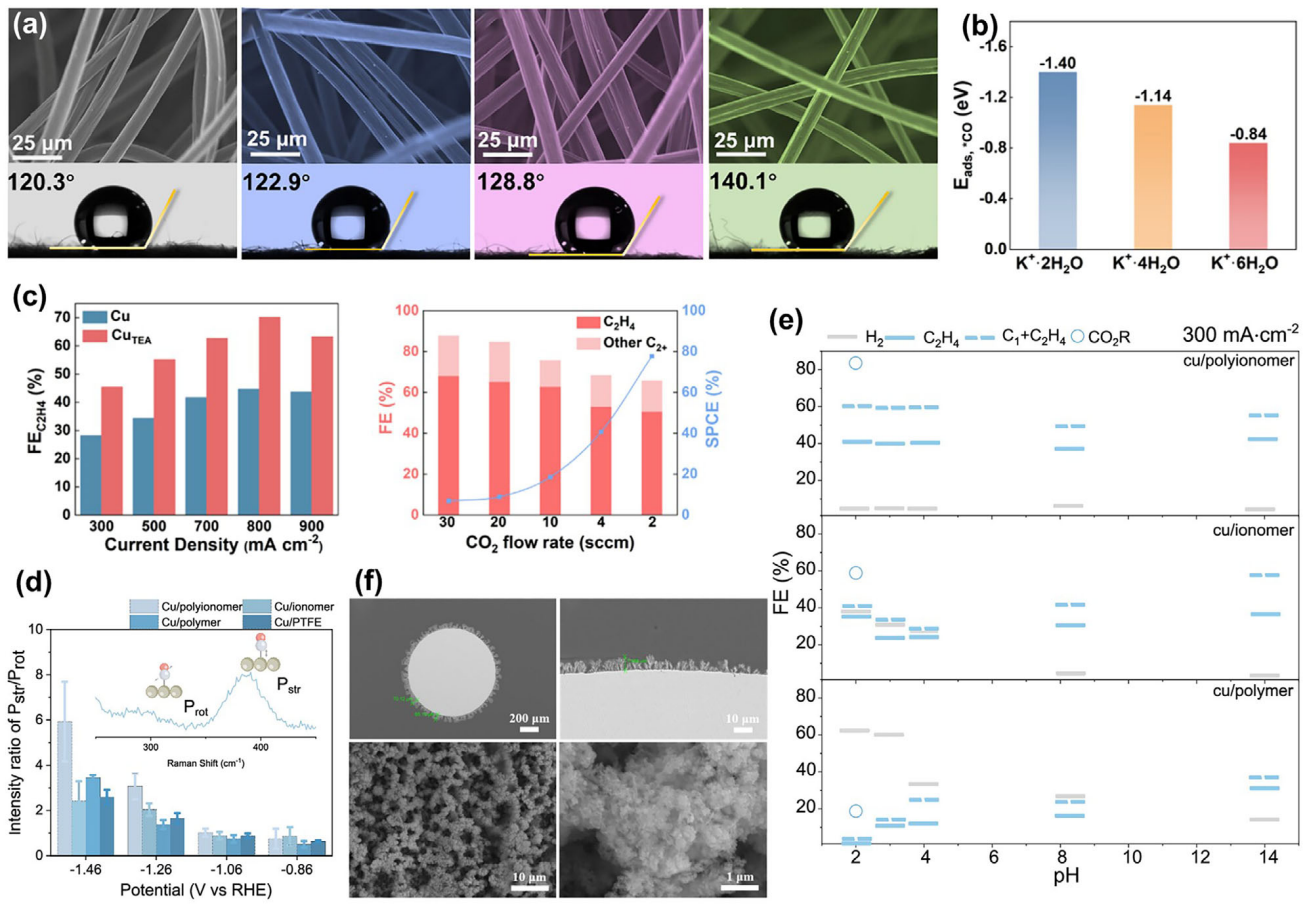
(a) SEM images (top) and contact angles (bottom) of pristine GF, Meo‐CoPc@GF, 7F‐CoPc@GF, and 13F‐CoPc@GF. Reproduced with permission [232]. Copyright 2025, Wiley‐VCH GmbH. (b) The effect of K^+^ hydration on *CO adsorption; (c) Relationship between $FE_{C_{2}H_{4}}$ and current density, and the relationship between $FE_{C_{2}H_{4}}$, $FE_{C_{2+}}$, and SPCE and CO_2_ (right). Reproduced with permission [233]. Copyright 2025, American Chemical Society. (d) Potential correlation between *CO coverage and P_str_/P_rot_ intensity ratio of Cu/PTFE, Cu/ionomer, Cu/polymer, and Cu/polyionomer; (e) Product distribution across a broader pH range. Reproduced with permission [234]. Copyright 2025, The Authors. (f) SEM images of the catalyst layer thickness. Reproduced with permission [235]. Copyright 2024, Elsevier B.V.

Introducing hydrophobic groups or π‐conjugated systems around catalytic sites can regulate local water distribution, optimize electron transfer pathways, and suppress HER. Li et al. pioneered an economical, efficient, and scalable method for preparing single‐molecule integrated catalytic electrodes with built‐in microenvironments [232]. They utilized the strong interfacial interactions between large π‐conjugated macrocyclic molecules and graphite‐derived carbon (GF) surfaces to readily integrate cobalt phthalocyanines modified with methoxy or perfluoroalkyl chains onto commercial GF, thereby obtaining micron‐scale single‐molecule integrated catalytic electrodes with an embedded microenvironment, free of conductive additives and binders, denoted as Meo‐CoPc@GF, 7F‐CoPc@GF, and 13F‐CoPc@GF (Figure 14a). The moderately hydrophobic 7F‐CoPc@GF (moderately fluorinated) constructs an intrinsic hydrophobic microenvironment that optimizes the three‐phase interface and electron transfer at active sites, stabilizes the *COOH intermediate, and promotes *CO desorption, thereby effectively suppressing the HER and enabling universally efficient CO_2_RR across acidic, neutral, and alkaline media. It displayed near 100% FE_CO_ at industrial‐scale current densities and maintained over 90% FE_CO_ even after prolonged operation at 100 mA cm^−2^ in acidic media. Additionally, this electrode could be applied to paired electrolysis systems (e.g., sulfur oxidation reaction coupling), significantly improving overall battery efficiency and electron utilization. This provides a new paradigm for the design of large‐scale, stable, and efficient single‐molecule integrated CO_2_ reduction electrodes and demonstrates broad potential in industrial exhaust gas treatment and various electrocatalytic processes. Another strategy enhances the stability of intermediates and C−C coupling by regulating the local ion concentration or proton transport channels on the electrode surface. Under acidic conditions, weak *CO adsorption and strong HER are the core issues limiting the formation of C_2+_ products. To address this issue, Yin et al. [233] proposed a strategy of ion‐polymer synergistic construction of local reaction zones. By adding tetramethylammonium hydroxide (TEAOH) to Cu catalyst ink, the Nafion polymer was induced to undergo microphase separation, reconfiguring the K^+^ distribution on the catalyst surface, reducing the content of K^+^‐bound water, and increasing the surface K^+^ concentration. This enhanced *CO adsorption and lowered the C−C coupling energy barrier, thereby achieving efficient C_2_H_4_ production. (Figure 14b) Under acidic electrolyte conditions (pH 1; Figure 14c), the Cu_TEA_ electrode achieved a C_2_H_4_ Faradaic efficiency of 70.2%, a total C_2+_ FE of 87.7%, and a single‐pass carbon efficiency of 77.6% at 800 mA cm^−2^, while maintaining stability for over 20 h. This work provides a scalable strategy for highly selective C_2_H_4_ generation in acidic CO_2_RR and offers mechanistic insights for industrial electrode design. Another ionomer polymer coating (PFSA‐PEI composite) was also investigated [234]. The coating simultaneously regulates local proton transport and *CO intermediate stability, enabling high selectivity for C_2+_ generation in acidic media. Specifically, the coating captured protons via the amine groups (–NH_4_^+^) of branched polyethyleneimine (PEI), while the sulfonic acid groups (−SO_3_^−^) and amphiphilic functional groups of PFSA regulated CO coverage (Figure 14d), local [CO_2_]/[H_2_O] ratio, and K^+^ environment. Hydrogen bonds between functional groups formed stable stereochemical assemblies, enabling the coating to maintain its structure and function over a wide pH range (2−14) (Figure 14e). In a flowing cell (0.5 M K_2_/H_2_SO_4_, pH 2) test, the optimized coating achieved a $FE_{C_{2+}}$ of 61%, ethylene FE of 40.1%, and single‐pass CO_2_ utilization of 84% at 0.3 A cm^−2^, C_2+_ conversion efficiency of 64%, representing approximately 30% and 35% improvements over the monofunctional coating, respectively, while HER inhibition was suppressed to below 15%. These results demonstrate that by regulating the local ionic environment or proton pathways, reaction selectivity under acidic conditions can be effectively optimized, providing a feasible strategy for the industrial production of high‐carbon products.

Additionally, by adjusting the pore structure and hydrophobic/hydrophilic gradients, the gas–liquid–solid three‐phase contact can be improved, reaction mass transport accelerated, and bubble retention reduced. Yan et al. [235] used a PTFE‐assisted hydrogen bubble dynamic template method to prepare three‐dimensional porous Zn@Ag electrodes with hydrophobic interiors and hydrophilic surfaces. (Figure 14f) The electrode not only maintains open channels but also significantly enhances the transport of gaseous CO_2_ to the active sites, achieving an FE_CO_ of over 92.1% in a KHCO_3_ microchannel reactor (−45 mA cm^−2^) and retaining more than 91% selectivity in a 1 M KOH flow cell (−250 mA cm^−2^), far surpassing the unmodified control. PTFE regulation resulted in smaller pore sizes and increased hydrophobicity, which together increased the local pH, enhanced the adsorption of *COOH, and suppressed HER. In situ ATR‐FTIR and Raman results indicated that this design promoted local CO_2_ enrichment and the *COOH pathway, almost completely suppressing formate generation. This strategy effectively solved the problems of low CO_2_ solubility and pore blockage, providing a feasible solution for the design of efficient non‐precious metal CO_2_RR catalysts.

These investigations demonstrate that diversified strategies for regulating the microenvironment through chemical interface control, including molecular/atomic‐level modification, hydrophobic treatment, ion‐polymer synergistic regulation, and ionomer coating construction, can significantly improve electrode stability, selectivity, and multi‐carbon product generation efficiency under acidic and neutral conditions, providing feasible solutions and mechanism‐based evidence for high‐current‐density industrial CO_2_RR.

#### 3.2.2 Regulation of Apparent Physical Structure of Electrodes

**FIGURE 15:**
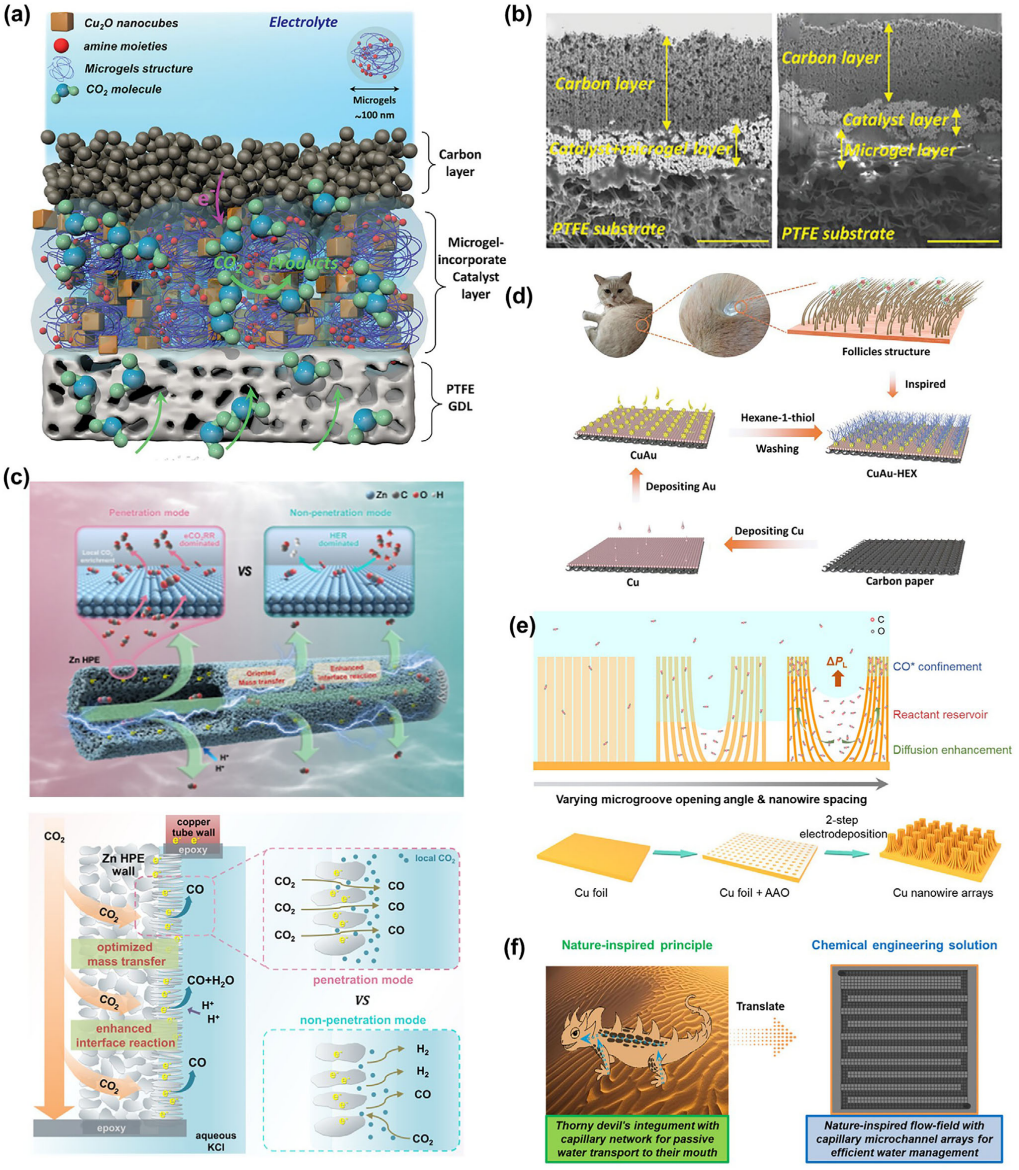
(a) Schematic of GDE with microgel‐modified catalyst layer and carbon black layer on PTFE; (b) Cross‐sectional FIB‐SEM of GDE. Reproduced with permission [236]. Copyright 2024, The Author(s). (c) Schematic structure of Zn HPE, and enhanced mass transfer at the gas–liquid–solid three‐phase interface due to the penetration effect. Reproduced with permission [237]. Copyright 2025, American Chemical Society. (d) Schematic design and synthesis of biomimetic hair follicle structure. Reproduced with permission [238]. Copyright 2025, Wiley‐VCH GmbH. (e) Schematic of liquid–solid–gas three‐phase and hierarchical structure NAMs. Reproduced with permission [239]. Copyright 2025, The Authors. Published by the American Chemical Society. (f) Schematic of bionic electrodes. Reproduced with permission [240]. Copyright 2025, The Authors. Published by the American Chemical Society.

Regulation of apparent physical structure focuses on the geometric morphology design at the macro and nanoscale, aiming to optimize the gas–liquid–solid three‐phase interface, improve reaction mass transport, and reduce bubble retention, thereby enhancing the reactivity and selectivity of CO_2_RR. Compared to chemical interface regulation, this strategy places greater emphasis on the macro‐scale layout of the electrode's pore network, roughness, catalyst particle arrangement, and gas diffusion channels. Based on their mechanisms of action, surface physical structure regulation can be categorized into the following types:

Microporosity and fine‐tuning of the catalytic layer. By constructing specific 3D structures within the catalyst layer, local CO_2_ enrichment and intermediate transport optimization can be achieved. Accordingly, Rabiee et al. [236] introduced pyridine‐containing microgels into a Cu_2_O‐based GDE to achieve precise regulation of the electrode microenvironment (Figure 15a,b). Owing to the affinity of their amine groups for CO_2_, the microgels could act as miniature CO_2_ reservoirs within the catalyst layer, thereby increasing the local CO_2_ concentration and promoting the formation of a three‐phase interface. This effectively overcame the issues of low CO_2_ solubility and mass transfer limitations commonly observed in conventional GDEs. Its three‐dimensional porous network structure also decreased diffusion impedance. At 700 mA cm^−2^, the FE for ethylene increased to 56%, and reached up to 58% in zero‐gap MEA, resulting in more than a twofold enhancement in ethylene yield. On this basis, rational design of the electrode framework and pore channel structure has become an important pathway to further enhance mass transfer efficiency. A zinc hollow fiber penetration electrode (Zn HPE) prepared through a combination of phase transformation, sintering, and electrochemical reduction has been developed [237]. Its unique hollow porous structure could force CO_2_ to penetrate the electrode wall and directly reach the catalytic interface, significantly improving the gas mass transfer limitation in traditional electrodes and maintaining an efficient three‐phase reaction environment even at ampere‐level current densities (Figure 15c). During CO_2_RR, the structure underwent in situ transformation into stable metallic Zn^0^ nanosheet active sites, which maintained a high active surface area while suppressing oxidative deactivation, thereby forming a persistent CO_2_‐enriched environment. This reduced the energy barrier for *COOH formation, increased the energy barrier for the HER, and consequently enhanced the selectivity toward CO generation. The electrode sustained ∼90% FE_CO_ at 800 mA cm^−2^ for 110 h, with a partial current density of 749.6 mA cm^−2^. Techno‐economic analysis further demonstrated that its low material cost and scalability significantly reduced CO production cost (approximately 385 USD per ton, with total cost far below reported systems), highlighting its potential for industrial application. Similarly, another strategy for mitigating bubble impact and optimizing local reactant distribution through pore and framework design was proposed by Xu and co‐workers [107]. Activated silver nanoparticles (Ag NPs) were grown in situ on the gas diffusion layer (GDL) framework to construct a binder‐free electrode, thereby achieving precise regulation of the interfacial microenvironment. While preventing direct exposure of Ag active sites to the electrolyte, this architecture effectively buffers bubble‐induced impacts through a continuous carbon nanoframework, reduces local gas flow velocity, and suppresses gas accumulation and electrode flooding. As a result, a high local CO_2_/H_2_O ratio and a K^+^‐enriched environment are maintained, thereby facilitating the formation and transport of reaction intermediates. In terms of performance, this electrode exhibited over 94% FE_CO_ at 400 mA cm^−2^ in acidic, neutral, and alkaline electrolytes while maintaining continuous operation at 100 mA cm^−2^ for 8 h in acidic medium. In addition, a high CO production rate (23.3 mol g^−1^ h^−1^) and single‐pass conversion efficiency (58.6%) were obtained.

In addition to conventional pore engineering, the introduction of biomimetic structures provides a novel regulatory approach for mass transport in CO_2_RR. A follicle‐like CuAu‐HEX electrode (Figure 15d) was constructed by anchoring hexanethiol (HEX) molecules onto Au sites via Au−S bonds, thereby forming a stable yet non‐dense hydrophobic layer and microchannels [238]. This architecture increases the local CO_2_/H_2_O ratio, suppresses the HER, and stabilizes the three‐phase interface without hindering reactant transport. The Au/Cu bimetallic sites decreased the *OC−COH coupling energy barrier and accelerated the formation of C_2+_ products. MD simulations and finite element analysis further showed that the hair‐like structure could efficiently introduce CO_2_ and form hydrophobic microchannels, alleviating the accumulation of liquid at the interface and maintaining the exposure of active sites. Even at a high current density of 1 A·cm^−2^, this electrode maintained 70% FE for C_2+_ products, highlighting the critical role of micro‐scale structural regulation in local microenvironment optimization. At the mesoscopic scale, microenvironmental optimization through multi‐dimensional structural design is equally remarkable. Cheng et al. [239] reported a superhydrophobic copper nanowire array (NAMs) with tunable micro‐groove structures (micro‐groove opening angles: 0°, 10°, 30°), which enabled multi‐dimensional optimization of interfacial wetting states, reactant transport, and intermediate kinetics (Figure 15e). Among them, NAM‐30 can stably establish a Cassie–Baxter wetting state with a gas layer thickness of approximately 8 µm, achieving a local CO_2_ concentration as high as 33.0 mol m^−3^. Meanwhile, the dense top‐layer structure prolongs the residence time of CO* intermediates, thereby significantly enhancing C─C coupling capability. In addition, the local pH near NAM‐30 was maintained in the range of 9.6∼10.4, suppressing HER (H_2_ FE reduced to 13.1%) and favoring a pathway where H^+^ participates in CO_2_RR rather than HER. Benefiting from these synergistic effects, the C_2+_ product selectivity of NAM‐30 improved by 72%, while the Faraday efficiency rose by 36%. This level of study demonstrates that through biomimetic and hierarchical geometric structural design, the local microenvironment can be intrinsically optimized, thereby overcoming the bottleneck of product selectivity toward higher‐order C_2+_ species. At a higher level, researchers have extended their regulatory approach to the flow field design of the entire electrolytic cell. Inspired by the capillary channels in the skin of desert lizards, a lizard‐inspired serpentine flow field was designed for a zero‐gap CO_2_ electrolyzer (Figure 15f), achieving passive water transport and efficient mass transfer [240]. This design solved the issues of CO_2_RR performance and stability limitations caused by salt deposition and water flooding in alkaline environments. In a 5.06 cm^2^ electrolyzer, the CO selectivity at 350 and 400 mA cm^−2^ was 46% and 97% higher than that of the traditional serpentine flow‐field cell, respectively. Micro‐CT imaging and electrochemical impedance spectroscopy analysis demonstrated that the flow field significantly reduced salt deposition and improved charge transfer, validating its mechanism of regulating water distribution through capillary microchannels, enhancing CO_2_ transport efficiency, and mitigating catalyst site blocking. This study highlights the combination of macro‐structural optimization and biomimetic inspiration, providing a holistic regulatory approach for industrial‐scale scaling‐up.

The regulation of apparent physical structure demonstrates a multi‐level progressive pattern from microporosity refinement, framework and pore channel construction, biomimetic hierarchical structure, to macro‐scale flow field design. Through cross‐scale synergistic optimization, the gas–liquid–solid three‐phase interface characteristics in CO_2_RR can be systematically improved, enhancing reactant transport and intermediate conversion efficiency, thereby achieving high activity, high selectivity, and long‐term stability in electrolytic performance. These studies provide an important design blueprint for developing scalable, high‐efficiency CO_2_ electrolytic devices in the future.

Beyond the microscopic chemical interface and apparent physical structure, the overall design of the electrolyzer and device plays an equally critical role in the industrial prospects of CO_2_RR. The device not only impacts the stability of the gas–liquid–solid TPB but also regulates local reactant concentrations, product removal, fluid dynamics, and current density distribution, determining product selectivity, energy efficiency, and long‐term stability under high current densities. Optimizing flow fields (i.e., serpentine, lizard‐like capillary channels, or hierarchical capillary networks) can enhance electrolyte circulation and CO_2_ transport efficiency, prevent local liquid stagnation and salt deposition, and maintain an efficient TPB. Zero‐gap or flow‐through cell designs permit rapid reactant supply and immediate product removal, maintaining a high local CO_2_/H_2_O ratio while suppressing the HER and stabilizing *CO and C─C coupling intermediates. Modular device integration allows for cross‐scale synergy by combining microscopic pore structures and hydrophilic/hydrophobic gradients with macroscopic flow‐field management, ensuring continuous reactant transport, reducing bubble blockage, and minimizing local overpotentials, thereby achieving high Faradaic efficiency, carbon utilization, and long‐term stability under industrial conditions. Therefore, optimization of the microenvironment in high‐performance electrodes must be considered in coordination with overall device design to realize CO_2_ electroreduction processes that are high in current density, selective, stable, and economically feasible, while also providing a systematic design template for industrial scale‐up and multiscale modeling.

### 3.3 Design of Electrolyte Systems and Regulation of Reaction Intermediates

**FIGURE 16:**
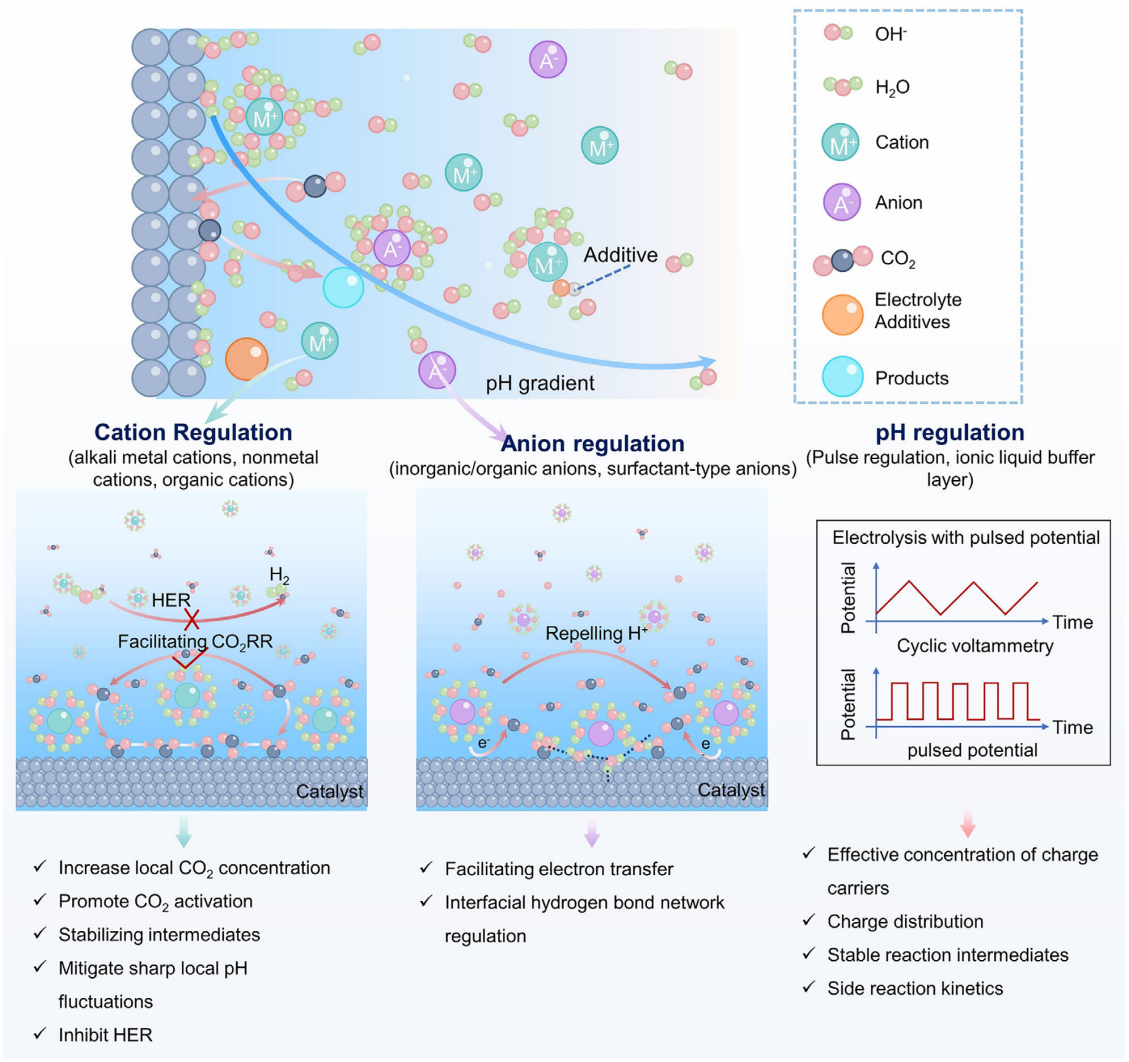
Schematic diagram of the microenvironment via electrolyte regulation, including cation regulation, anion regulation, pH, and buffering capacity regulation.

Electrolytes, as key participants in the interfacial microenvironment, play multifaceted roles in regulating electric field strength, interfacial ion concentration, solvent structure, and pH gradients. Their composition, concentration, and physicochemical properties collectively shape the so‐called “solution microenvironment”, which directly determines the steady‐state distribution of reaction intermediates and the energy barrier crossing pathways, serving as a critical lever for achieving efficient and selective CO_2_ electro‐reduction. To facilitate systematic discussion, this section categorizes electrolyte regulation strategies into the following four types: cation regulation; anion regulation; pH and buffering capacity regulation; and other electrolyte systems and additives (Figure 16).

#### 3.3.1 Cation Regulation

Due to differences in radius, polarizability, and hydration ability, different cations have a significant impact on the interfacial electric field and reaction pathway selection. Under normal circumstances, the larger the radius of an alkali metal cation, the weaker its hydration ability, making it easier to approach the electrode surface, thereby compressing the electric double layer and forming a stronger oriented interfacial electric field (OIEF), which promotes CO_2_ activation and C−C coupling. In fact, as an essential component of electrolytes, cations play a role far beyond the compression of the electric double layer. They profoundly influence the selectivity and kinetics of CO_2_ reduction reactions through their size, structure, and local interaction modes. At the electrode‐electrolyte interface, they exhibit a “dual role”: both as builders of electric fields and as indirect regulators of reaction selectivity. However, the role of cations is not a simple enhancing effect but exhibits complex concentration dependence and configuration sensitivity.

**FIGURE 17:**
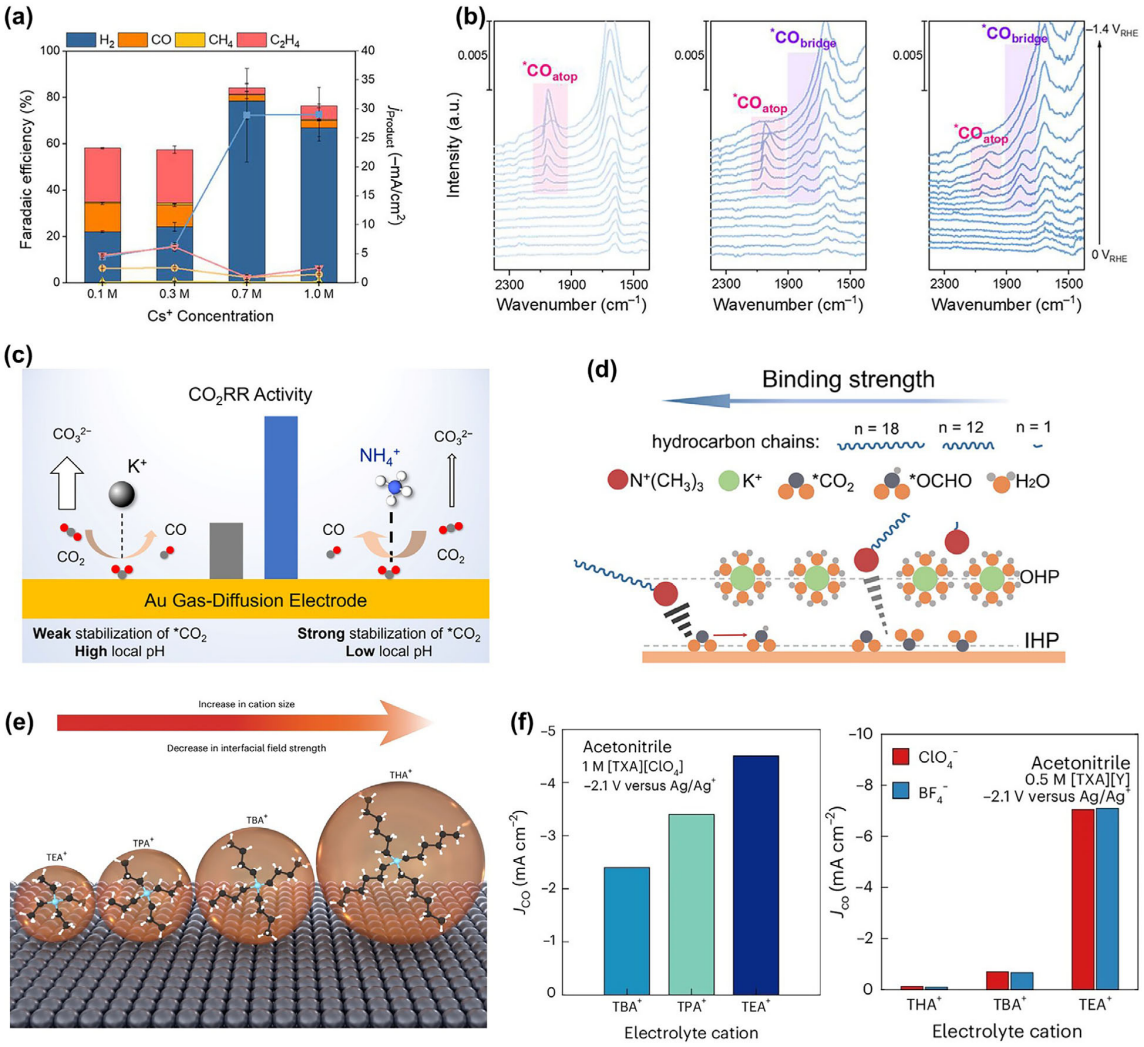
(a) Cs^+^ concentration–dependent product distribution and partial current density at −1.2 V_RHE_; (b) ATR‐SEIRAS of sputtered Cu at different potentials. (From left to right: 0.05, 0.15, and 0.35 M Cs_2_SO_4_). Reproduced with permission [241]. Copyright 2025, The Authors. Published by American Chemical Society. (c) Schematic of the role of NH_4_^+^. Reproduced with permission [242]. Copyright 2025, American Chemical Society. (d) Spatial effect of CnTA^+^ cations with different main chain lengths on binding strength of intermediate. Reproduced with permission [244]. Copyright 2025, Wiley‐VCH GmbH. (e) Schematic of the effect of different organic cation radii on CO_2_RR; (f) The effect of ionic strength and counterion of the electrolyte on the CO_2_R rate. Reproduced with permission [245]. Copyright 2025, The Author(s), under exclusive licence to Springer Nature Limited.

Previous studies have primarily focused on alkali metal ions. Typically, in acidic systems catalyzed by Cu, K^+^ promotes C_2_H_4_ generation, while high concentrations of Cs^+^ (0.7–1.0 M) significantly enhance HER and suppress CO_2_RR activity (Figure 17a) [241]. Using spectral characterization combined with electrochemical product analysis (Figure 17a,b), it was found that as Cs^+^ concentration increased from low (0.1–0.3 M) to high (0.7–1.0 M), the adsorption of *CO intermediates on the Cu surface gradually shifted from the top position (CO_atop_), which favors C─C coupling, to the bridge position (CO_bridge_). This leads to a decrease in C_2_H_4_ selectivity, a significant enhancement of HER, with a H_2_ FE of 78%. This mechanism is also applicable to Ag catalysts. However, due to the faster desorption rate of *CO on Ag, the performance decline was less pronounced compared to Cu. Additionally, introducing polymer binders (e.g., Nafion and PTFE) on the Cu surface to block direct cation interaction could suppress this process. With further research, scientists have gradually discovered the unique role of non‐metallic cations [242]. Compared to Na^+^ and K^+^, NH_4_^+^ not only significantly enhanced the CO generation rate on Au electrodes but also stabilized the *CO_2_ intermediate through hydrogen bonding between N─H and adsorbed CO_2_, while mitigating local pH fluctuations (Figure 17c). As NH_4_^+^ increased from 0.1 M to 2.0 M, j_CO_ reached 300 mA cm^−2^, approximately three times that of the Na^+^ system, with a reaction order of 0.365, higher than K^+^ (0.271) and Na^+^ (0.197), indicating that NH_4_^+^ promotes CO_2_RR by influencing the rate‐limiting step rather than directly participating in the reaction. At 200 mA cm^−2^, the local pH at the interface of the NH_4_^+^ system was only 8.9–9.0, while that of the Na^+^/K^+^ system was approximately 13, effectively suppressing the side reaction between CO_2_ and OH^−^. This promoting effect was observed across Au nanoparticles of different sizes (3.5, 6.7, and 8.5 nm), indicating that the underlying mechanism is general. More detailed mechanistic studies revealed that specific organic cations may also exert differential regulation on different coordination sites on the catalyst surface. For example, the cation cetyltrimethylammonium (CTA^+^) almost exclusively enhanced the activity of under‐coordination (UC) sites on the Ag electrode [243]. These sites occupied only about 2% of the total surface area of the polycrystalline silver electrode, but in the presence of CTA^+^, the TOF of CO generation at these sites increased by more than three orders of magnitude compared to high coordination (HC) sites, thereby enabling low coordination sites to dominate the overall reaction activity and increasing the conversion rate of CO_2_ to CO by nearly 40‐fold. Further investigations revealed that these critical sites are highly sensitive to trace impurities in the solution, with even small amounts of impurities completely negating the enhancing effect of CTA^+^. This phenomenon was observed for both CO and HCOOH generation on silver and tin electrodes. This finding not only highlights the high site‐dependence of cation‐interface interactions but also emphasizes the necessity of synergistic optimization between microenvironment regulation and catalyst structure. When the molecular structure was extended to long‐chain quaternary ammonium cations (C_n_TA^+^), the spatial effect was further highlighted (Figure 17d) [244]. In the CO_2_RR system on copper electrodes, CnTA^+^ were introduced as electrolyte additives, enabling directed regulation of the adsorption of reaction intermediates. As the alkyl chain length increases, the longer‐chain cations carry higher positive charges, thereby enhancing the electrostatic interactions with the negatively charged catalyst surface. This stabilizes the *OCHO intermediate via hydrogen bonding while partially replacing the hydrated K^+^ on the electrode surface, promoting the reaction pathway toward formic acid formation and suppressing hydrogen evolution side reactions. When *n* = 18, the organic cation system achieves up to 90% HCOOH selectivity while maintaining good stability. This phenomenon demonstrates the potential of organic cations in regulating product selectivity.

In nonaqueous systems, organic small‐molecule cations exhibited another mechanism. For example, Tetrakisammonium (TXA^+^) ions exhibited a mechanism different from that of metal ions in low‐polarity solvents such as acetonitrile [245]. The cations determine their physical adsorption distance to the electrode through their size and geometry, thus modulating the interfacial electric field intensity. The strengthened interfacial field significantly lowers the activation barrier for CO_2_ → CO_2_˙^−^ (i.e., the rate‐limiting step of CO_2_ activation), and this effect is independent of electrolyte concentration or anion type (Figure 17e,f). Therefore, in nonaqueous systems, cations primarily accelerate CO_2_RR via a “distance−field” effect rather than through direct chemical interactions.

From the radius effect of alkali metal ions to the hydrogen bonding stability of non‐metal ions, and further to the electric field regulation and spatial effects of organic cations, the cation action mechanism has expanded from a “simple electric double layer compression” to a multidimensional framework. This hierarchical understanding not only deepens our understanding of the interfacial microenvironment but also provides rich ideas for achieving precise control of product pathways through ion engineering.

#### 3.3.2 Anion Regulation

Unlike cations, anions mainly influence CO_2_RR kinetics through competitive adsorption, local pH regulation, and interfacial structural rearrangement. As an important component of electrolytes, anions not only determine the acidity and ion strength of solutions but also profoundly influence the selectivity and kinetics of CO_2_RR through physical adsorption, hydrogen bond network reconstruction, and interfacial electronic effects. Compared with cation effects, anion effects exhibit greater diversity in their modes of action, with their patterns evolving from the basic effects of single inorganic ions to the interfacial regulation by organic anions, and further to specific adsorption and multi‐ion synergistic effects, gradually forming a systematic understanding of their mechanisms.

**FIGURE 18:**
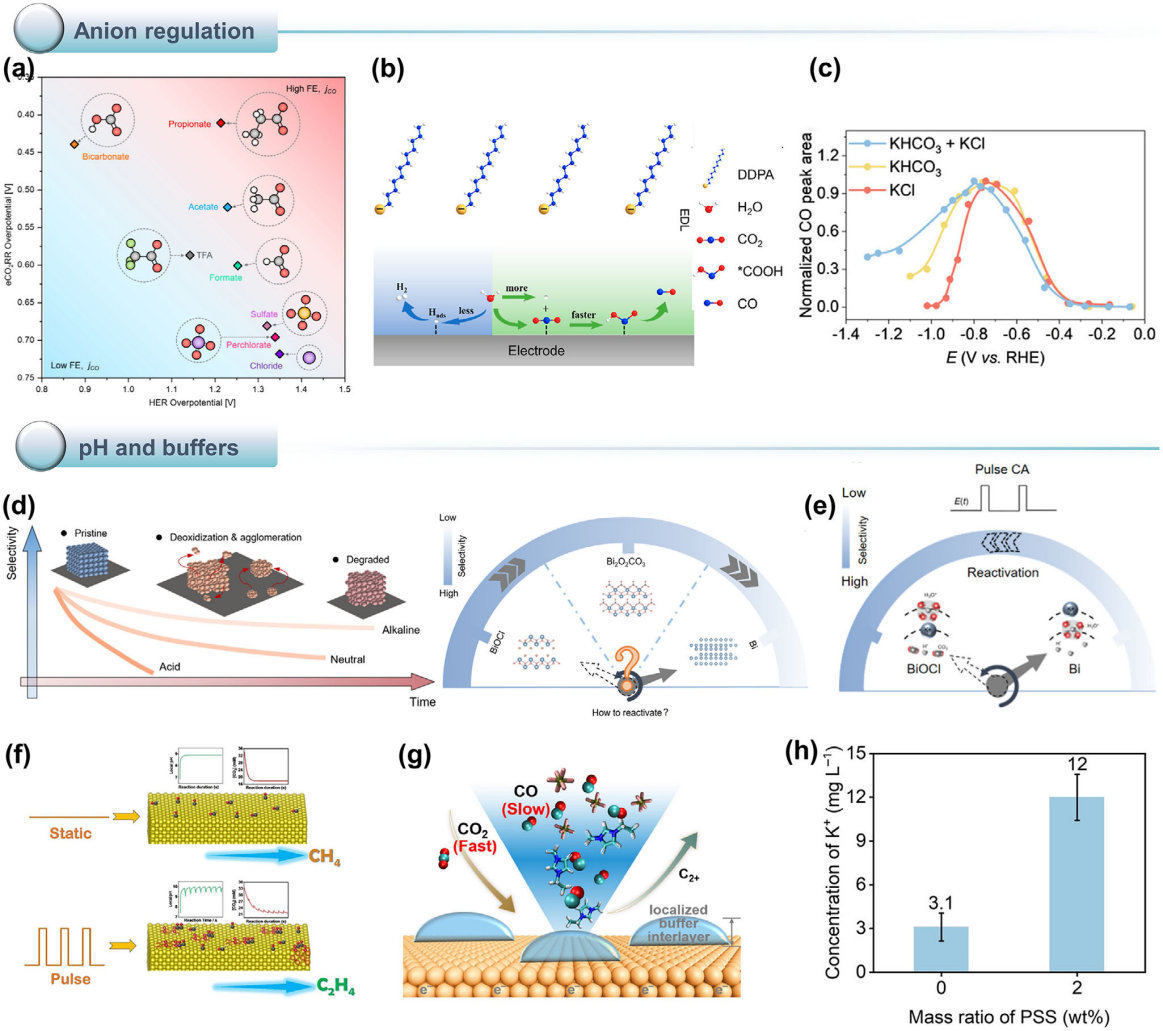
(a) Overpotential of HER and eCO_2_RR for electrolytes with different anions. Reproduced with permission [246]. Copyright 2024, The Authors. (b) Schematic of DDPA effects. Reproduced with permission [247]. Copyright 2024, American Chemical Society. (c) Normalized *CO peak area. Reproduced with permission [249]. Copyright 2025, Wiley‐VCH GmbH. (d) Schematic of catalyst degradation in acidic, neutral, and alkaline electrolytes and dynamic deactivation mechanism of Bi‐based catalysts; (e) Catalyst reactivation by pulse CA technology. Reproduced with permission [250]. Copyright 2024, American Chemical Society. (f) C_2_H_4_ generation under pulse electrolysis. Reproduced with permission [251]. Copyright 2025, American Chemical Society. (g) Localized buffer interlayer for CO buffer around the catalytic surface by IL interlayer. Reproduced with permission [252]. Copyright 2025, American Chemical Society. (h) Comparison of K^+^ adsorption concentrations with/without PSS. Reproduced with permission [253]. Copyright 2024, Wiley‐VCH GmbH.

Using an Au electrode as a model, a systematic comparison was conducted on the influence of inorganic anions such as HCO_3_^−^, ClO_4_^−^, SO_4_^2−^, and Cl^−^, as well as structurally similar carboxylate anions (e.g., CH_3_COO^−^, C_2_H_5_COO^−^, HCOO^−^, CF_3_COO^−^), on the competition between eCO_2_RR and HER (Figure 18a) [246]. This study revealed that the type of anion significantly affects the double‐layer structure and the environment of reaction intermediates. Inorganic anions, in the absence of bicarbonate‐assisted proton transfer, can suppress HER but simultaneously lead to reduced CO yield and higher reduction overpotentials. In contrast, carboxylate anions, through moderate physical adsorption and modulation of the interfacial hydrogen‐bond network, achieve a balance between HER suppression and maintaining CO production rates. Among them, propionate exhibited the best performance, with an FE_CO_ of approaching 100%, a CO generation rate comparable to that of bicarbonate, but with superior selectivity. This work proposes anion adsorption energy as a new structural descriptor, providing theoretical guidance for electrolyte optimization. In recent years, research has further expanded to anionic surfactant systems. Unlike conventional small‐molecule anions, surfactant anions are capable of self‐assembly at the interface, dynamically reconstructing the local hydrogen‐bond network of water molecules, which was demonstrated by Han and co‐workers [247]. Taking dodecylphosphonic acid (DDPA) as a representative, the anionic surfactant undergoes interfacial self‐assembly rather than irreversible adsorption onto the catalyst surface. By restructuring the interfacial hydrogen‐bonding environment, it increases the proportion of weakly bonded water molecules, thereby optimizing proton transfer and facilitating the hydrogenation of CO_2_ to the *COOH intermediate. This enables efficient CO production while significantly suppressing HER (Figure 18b). In flow cells, FE_CO_ remained above 90% within the current density range of 50∼400 mA cm^−2^; even in acidic electrolytes (pH 2.5), FE_CO_ was maintained at 81% at 300 mA cm^−2^. These findings indicate that anions are no longer merely static “background ions”, but can impart new functional characteristics to the interface through molecular self‐assembly.

While carboxylate anions and surfactant anions primarily exert their effects by regulating interfacial hydrogen bonding and protonation processes, halide anions exhibit more direct electronic effects. Zhu et al. [248] systematically investigated the roles of different anions (HCO_3_^−^, Cl^−^, Br^−^, I^−^) in the catalytic process on In_2_O_3_ nanofiber catalysts rich in oxygen vacancies. The results showed that Cl^−^ adsorbed on the indium surface altered the electronic structure, significantly reducing the Gibbs free energy (ΔG) for the conversion of the key intermediate *OCHO to *HCOOH, thereby enhancing HCOOH selectivity and suppressing HER. In contrast, Br^−^ and I^−^ enhanced *H formation, promoted HER, and reduced HCOOH selectivity. On In_2_O_3_ electrocatalysts, the order of electrocatalytic activity and selectivity for CO_2_ conversion to HCOOH was KHCO_3_ < KBr < KI < KCl. Utilizing this anion regulation strategy, the authors also constructed a dual‐electrode system, replacing the oxygen evolution reaction (OER) at the anode with glycerol oxidation, achieving simultaneous formic acid production at both electrodes. The overall FE_HCOOH_ reached 188.5%, significantly improving energy conversion efficiency. This indicates that the specific adsorption behavior of different anions can directly shape the electronic environment at the reaction interface, thereby enabling precise control of product selectivity.

The recently proposed mixed anion strategy has displayed significant potential at industrial‐scale current densities. By designing a mixed anionic electrolyte (2 M KOH + 1 M KCl) and combining it with a high‐surface‐area Cu_2_(OH)_3_F precursor catalyst, the system investigated the regulatory role of anions in ethanol selectivity during eCO_2_RR [249]. At an industrial‐scale current density of 700 mA cm^−2^, it attained a FE_EtOH_ of 50%, a C_2+_ product efficiency of 93%, and a cathode energy efficiency of ∼30%. This is attributed to the synergistic action of OH^−^ and Cl^−^ in the local microenvironment. Both can increase surface CO coverage (Figure 18c), contributing to C─C coupling to form C_2_ intermediates. In this way, OH^−^ stabilizes the key branch intermediate *CHCOH by forming hydrogen bonds with hydroxyl groups adsorbed on the catalyst surface and suppresses its dehydration side reaction, while Cl^−^ enhances the reactivity of *CHCOH by accelerating water decomposition, enabling it to preferentially convert into intermediate compounds leading to ethanol (such as *HCCHOH and *OCH_2_CH_3_). This “division of labor” model breaks through the limitations of single anion regulation and opens up the possibility of efficient C_2+_ generation under high current conditions.

Through analysis of the above literature, it is clear that the anion effect has gradually expanded from the basic laws of single ions to complex interfaces and synergistic regulation. By revealing the role of anions in double‐layer structures, hydrogen bond networks, and electronic effects, we have not only deepened our understanding of electrolyte‐interface interactions but also provided new strategies for achieving efficient CO_2_ electrolysis through electrolyte design.

#### 3.3.3 pH and Buffers

The pH of the electrolyte is a key variable in the regulation of the interfacial microenvironment. It not only determines the effective concentration of electron donors but also fundamentally shapes the selectivity and stability of CO_2_RR by influencing local charge distribution, reaction intermediate stability, and side reaction kinetics. In recent years, researchers have systematically explored the mechanisms of pH and buffer agents under acidic, neutral, and high current density conditions, and have gradually proposed a series of solutions based on microenvironment regulation.

In acidic systems, catalyst stability is closely related to local pH. Liu et al. [250] found that in low‐concentration K^+^ acidic electrolytes, traditional BiOCl nanosheets undergo a phase transformation from BiOCl to Bi_2_O_2_CO_3_ and then to metallic Bi during the reaction process, accompanied by a rapid decline in FE and enhanced HER, indicating that catalyst degradation is the primary cause of low stability in acidic CO_2_RR (Figure 18d). The pH of the catalyst surface microenvironment shifts from neutral to acidic as the catalyst degrades, while the surface charge governs the competitive adsorption of K^+^ and protons, thereby modulating the interfacial water network and COOH* adsorption, which ultimately affects reaction selectivity. To address this issue, they proposed a pulsed potential (AC) strategy, which maintains the original phase state of BiOCl through periodic reactivation, preventing its complete reduction to elemental Bi (Figure 18e). This approach also stabilizes the local pH near neutrality, significantly enhancing the long‐term stability of acidic CO_2_RR. These results indicate that dynamic pH regulation and phase stabilization are critical for maintaining selectivity and longevity in acidic environments. In contrast, under neutral conditions, research has focused more on the correlation between local pH dynamic regulation and product selectivity. Using single‐crystal Cu(100) as a model, Gong et al. [251] revealed that the selectivity of C_2_H_4_ and CH_4_ is highly dependent on dynamic fluctuations in interfacial pH. By designing alternating pulse potentials between the cathode and anode and adjusting the anode pulse width, researchers can precisely control the local pH near the Cu surface, allowing the anode pulse to provide time for OH^−^ ion diffusion, thereby buffering the adverse effects of local pH increases during the cathode pulse on CH_4_ production. Simultaneously, during the anode period, an appropriate amount of Cu^+^ sites are formed (Figure 18f), which synergistically accelerate *CO dimerization, thereby enhancing the activity of C_2_H_4_ production. This study uncovers the mechanism by which pulsed potentials achieve product‐oriented control through the simultaneous regulation of local pH and valence state distribution, providing a useful strategy for selectivity optimization in other electrocatalytic systems.

Under high current density conditions in industrial applications, relying solely on potential strategies is insufficient to balance mass transfer and stability, necessitating the introduction of engineered buffer layer designs to ensure both stability and high productivity. A study on “local buffer intermediate layers” proposed introducing a 1 µm‐thick ionic liquid (IL, Emim‐PF_6_) interlayer on the surface of a copper catalyst (Figure 18g), achieving simultaneous regulation of mass transfer between CO_2_ and the intermediate *CO [252]. This interlayer extended the local residence time of *CO by threefold (from 200 to 600 s) through dipole−hydrogen bonding interactions, while promoting the HCO_3_^−^/CO_2_ buffering reaction. Additionally, it maintained the dynamic equilibrium between Cu^+^ and Cu^0^ through electrostatic interactions, regulating the dynamic reconstruction of Cu_2_O, thereby inhibiting the deactivation of the copper‐based catalyst. In a 100 cm^2^ flow‐through cell, the C_2+_ yield reached 196 mmol h^−1^, the single‐pass carbon conversion rate was 35%, and carbon loss was <6%. Under an industrial current of 70 A, the catalyst maintained a decay rate of <5% after continuous operation for 60 h, demonstrating exceptional stability. The IL interlayer forms a “molecular sponge” effect, successfully achieving precise mass transfer control of “retaining CO_2_ and limiting CO”, providing a feasible solution for selective C_2+_ production and industrial‐scale production under high current density conditions.

The mechanism of action of pH and buffers evolves from phase stability in acidic systems, to product selection under neutral conditions, and finally to high‐current buffer design at an industrial scale, gradually revealing the evolution from theoretical understanding to practical application. Related research not only reveals the core regulatory role of the pH microenvironment in CO_2_RR but also provides direction for developing targeted electrolyte design strategies under different operating conditions in the future.

In addition to conventional regulation of cations, anions, and pH buffers, the introduction of functionalized electrolyte additives represents a more flexible and targeted strategy for engineering the interfacial microenvironment. By incorporating ionic liquids, organic small molecules, or polyelectrolytes into the electrolyte, it is possible to precisely regulate local electric fields, hydrogen bond networks, and ion distribution without altering the main electrolyte, thereby enhancing the selectivity and stability of CO_2_RR. By introducing the polyelectrolyte polystyrene sulfonate (PSS) as an additive into the electrolyte, Wang et al. [253] found that its anions can enrich K^+^ at the interface through electrostatic interactions and form hydrogen bond networks with H_3_O^+^, thereby weakening proton migration kinetics and effectively suppressing the HER (Figure 18h). Concurrently, the steric hindrance of PSS reconfigures the interfacial water hydrogen bond network, accelerating the formation of CO_2_RR intermediates and charge transfer processes. This strategy enabled commercial Ag catalysts to achieve a CO Faradaic efficiency of 93.9% and a single‐pass carbon efficiency of 72.2% under acidic conditions, maintaining >90% stability for 12 h, demonstrating the potential of polyelectrolytes to enhance selectivity and stability in acidic systems. Electrolyte additive strategies can overcome the limitations of traditional cationic, anionic, and pH‐regulated approaches by regulating different reaction environments through mechanisms such as interfacial charge rearrangement, hydrogen bond network restructuring, and local ion enrichment. Whether under high current density or acidic electrolyte conditions, appropriately designed functionalized additives offer new pathways for achieving high selectivity and stability in catalysts.

The regulation of the interfacial microenvironment in CO_2_ electrolysis involves multiple factors, including catalyst active site design (3.1), electrode interface/structure optimization (3.2), and electrolyte system engineering (3.3). The electronic structure of the catalyst active sites determines the initial adsorption and reaction pathways of intermediates; while electrode interface structure influences mass transfer, proton transfer, and bubble desorption through gas–liquid–solid three‐phase regulation and hydrophilic/hydrophobic design. Additionally, electrolyte composition and local pH evolution further shape electric field strength and ion distribution, thereby determining reaction selectivity and stability. These elements highly coordinate at both temporal and spatial scales, forming a complex reaction microenvironment, whose synergistic regulation has become a key driver for advancing CO_2_RR toward efficient, highly selective, and scalable directions.

However, current research still faces several challenges. First, the multi‐variable cross‐effects of interfacial behavior are difficult to separate, such as cations, pH, and local electric fields often being mutually coupled, leading to unclear mechanistic insights. Second, many strategies perform excellently under laboratory conditions but still face issues of deactivation and mass transfer limitations under industrially relevant high current densities and prolonged operation. Finally, while in situ/operational characterization and multiphysics simulations have revealed some dynamic processes, a unified understanding of interfacial evolution under real reaction conditions remains elusive. Therefore, future efforts urgently require the development of cross‐scale in situ characterization and simulation methods to enable dynamic monitoring of the electrode–electrolyte–gas three‐phase interface; simultaneously, integrating microenvironment design with device engineering is needed to transition from single‐factor regulation to multidimensional synergistic optimization. By establishing a comprehensive strategy spanning from molecular regulation to reactor integration, it is anticipated that the fundamental breakthroughs in the selectivity bottleneck and stability challenges of CO_2_RR can be achieved, laying a solid foundation for the carbon resource utilization driven by renewable energy.

## 4 External Field‐Assisted Regulation of eCO_2_RR

In traditional electrocatalytic CO_2_ reduction systems, reaction performance has long been dependent on static control of the intrinsic structure of the catalyst and the reaction medium. In the previous chapter, we focused on intrinsic regulatory measures at the microscopic level, such as catalyst design, ion concentration adjustment, interface pH buffering, electrolyte additives, and electrode geometric configurations. These strategies have demonstrated significant effects in improving CO_2_ adsorption, stabilizing key intermediates, and suppressing the HER. Such approaches primarily rely on static design of catalyst structure or electrolyte composition, inherently belonging to “pre‐set” regulation, providing the necessary static foundation for improving eCO_2_RR. However, static design struggles to respond promptly to transient interface fluctuations. Certain optimizations may fail under high current or complex feed conditions (containing O_2_ or impurities); moreover, structural optimizations often involve unavoidable trade‐offs (e.g., enhancing adsorption vs. promoting desorption, increasing activity vs. amplifying side reaction pathways).

**FIGURE 19:**
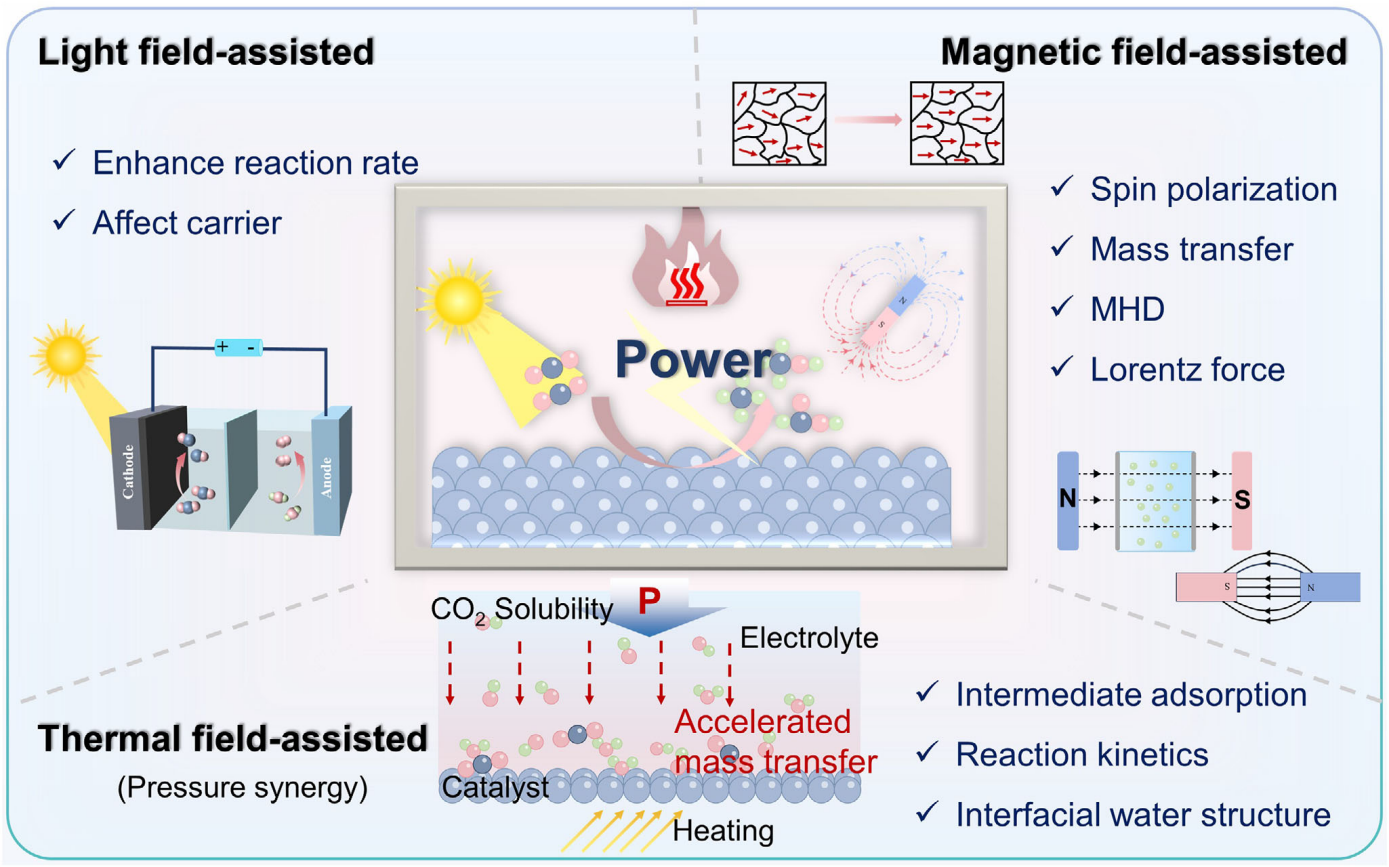
Schematic diagram of field‐assisted microenvironment regulation.

Therefore, introducing an external field as an “exogenous” intervention can compensate for the shortcomings of these static strategies. The external field can serve as a dynamic controller without altering the catalytic sites, regulating the microenvironment on demand across temporal and spatial scales, thereby unlocking new avenues for controlling catalytic processes. This chapter will focus on three typical types of external fields (Figure 19): (1) thermal field assistance; (2) light field assistance; and (3) magnetic field assistance. Each section will discuss how these fields act on interfacial charge distribution, intermediate stability, interfacial solvent structure, and synergistic reaction pathway regulation, and will explore their scientific mechanisms and engineering prospects using in situ/real‐time characterization methods.

### 4.1 Thermal Field‐Assisted eCO_2_RR

In the eCO_2_RR system, the thermal field serves as an external regulatory mechanism that can precisely control the reaction microenvironment, adsorbed intermediates, product distribution, and reaction kinetics without altering the intrinsic structure of the catalyst. This is achieved by adjusting the temperature and temperature gradient of the reaction system. Temperature not only affects reaction rates but can also selectively regulate the formation of C_1_/C_2_ and long‐chain products by altering adsorption free energy, interfacial water structure, and ion migration behavior, thereby suppressing side reactions (e.g., HER) and enhancing the selectivity of multi‐carbon products.

**FIGURE 20:**
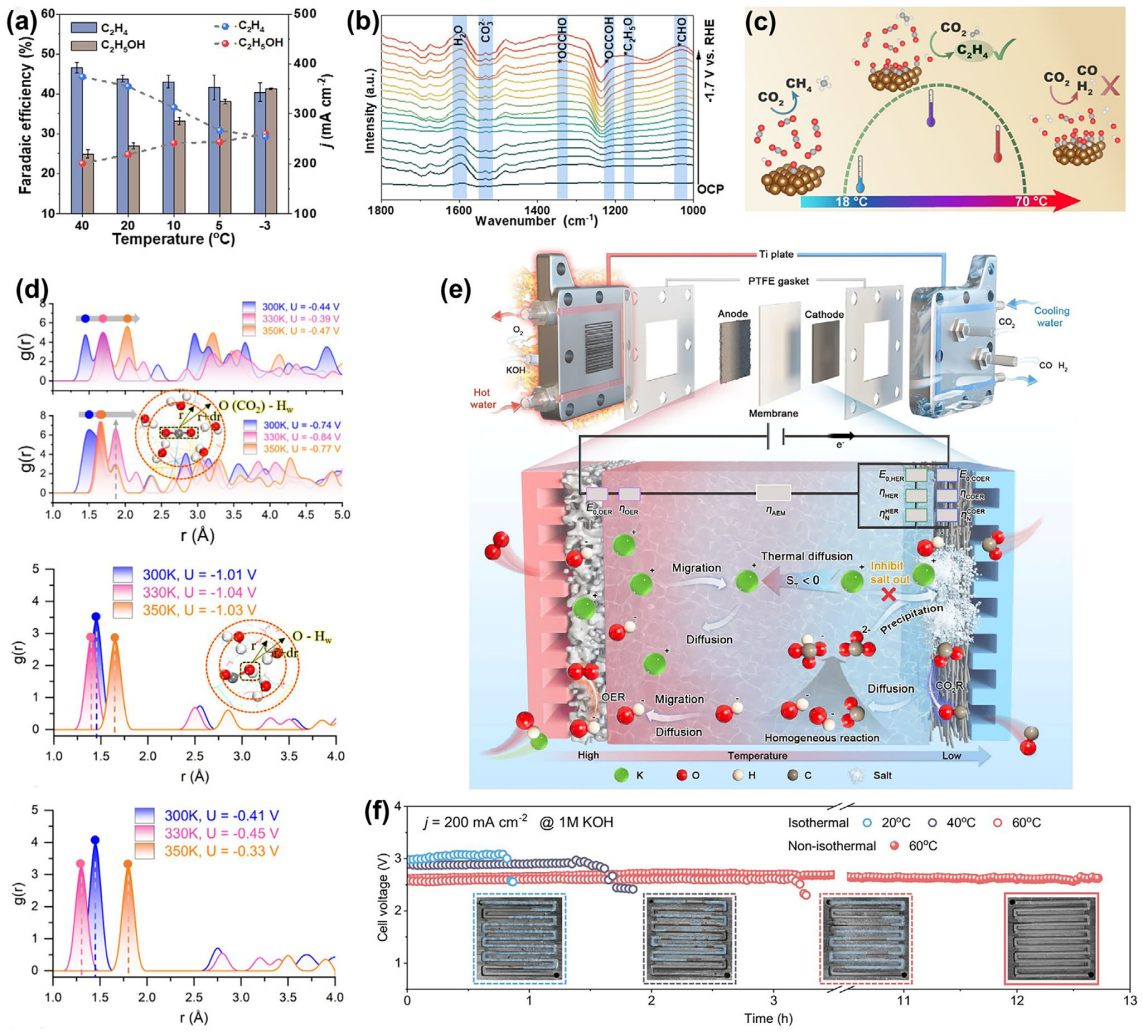
(a) $FE_{C_{2}H_{4}}$/$FE_{C_{2}H_{5}\mathrm{OH}}$ at −1.3 V_RHE_; (b) In situ ATR‐SEIRAS on the Cu‐NR at electrolyte temperatures of −3°C. Reproduced with permission [254]. Copyright 2025, Science China Press. Published by Elsevier B.V. and Science China Press. (c) Schematic display of catalysis at different temperatures. Reproduced with permission [255]. Copyright 2023, The Authors. Published by American Chemical Society. (d) Radial distribution functions of the O atoms in adsorbed *CO_2_ and the H atoms in interfacial water molecules, as well as the O atoms on the linked −OH side of *COOH intermediate and the H atoms in interfacial water at different temperatures and potentials. (Color codes: exposed surface Au, blue; exposed staple Au, turquoise; C, gray; O, red; and H, white). Reproduced with permission [257]. Copyright 2025, American Chemical Society. (e) Schematic of MEA‐based electrolyzer and the reaction and ion transport mechanisms; (f) Anti‐precipitation performance under isothermal and non‐isothermal conditions. Reproduced with permission [258]. Copyright 2025, The Author(s).

In Cu‐based catalyst systems, low temperatures have been shown to significantly alter product selectivity [254]. Combining theoretical calculations with experiments reveals that reduced temperatures weaken interfacial water activity and alter the adsorption free energy of *H/*CO, thereby inhibiting the HER, enhancing *CO coverage, and facilitating C─C coupling, which drives the product shift from ethylene to ethanol. Specifically, (Figure 20a) experiments on Cu nanorod electrodes at temperatures ranging from −3 to 40 °C showed that as the temperature decreases, $FE_{H_{2}}$ and FE_CO_ gradually decreases, while $FE_{C_{2+}}$ and $FE_{C_{2}H_{5}\mathrm{OH}}$ significantly increase. When the electrolyte temperature was lowered from 40°C to −3°C, the $FE_{C_{2}H_{4}}$/$FE_{C_{2}H_{5}\mathrm{OH}}$ ratio decreased from 1.86 to 0.98, achieving a shift in the main product from ethylene to ethanol. Notably, at −3°C, the FE of the C_2+_ product reached as high as 90.1%. In situ spectroscopy (Figure 20b) and electrochemical characterization further confirmed that low temperatures enhance *CO adsorption, increase K^+^ hydration leading to reduced water activity, and prioritize the formation of ethanol via the *CH‐CHOH pathway from the *CH‐COH intermediate, while relatively inhibiting ethylene production. Thus, temperature provides a clear mechanistic guidance for optimizing CO_2_RR selectivity by synergistically regulating adsorption thermodynamics, interfacial microenvironment, and reaction kinetics. Further systematic studies on Cu catalysts reveal a two‐stage effect of temperature on Cu‐based CO_2_RR (Figure 20c) [255]. In the 18−48°C range, as temperature increases, *CO coverage increases and gradually migrates from smooth sites to defect sites, thereby strengthening C−C coupling, resulting in the Faradaic efficiency of C_2+_ products reaching a maximum at ∼48°C, while CH_4_ and HCOOH selectivity decreases and HER remains largely constant; In the 48∼70°C range, due to Cu surface reconstruction and deactivation of defect sites, CO_2_RR activity decreases, and HER becomes dominant. C_2+_ product formation sharply declines, while C_1_ product increases. By controlling CO_2_ partial pressure, the primary influence of CO_2_ solubility changes on the trend was eliminated, indicating that temperature directly affects the catalytic mechanism. This suggests that thermal field regulation not only enables product reversal by altering temperature but also identifies an “optimal window” (∼45°C) within the mid‐temperature range, which is of significant importance for thermal management in industrial electrolytic cells.

Unlike Cu, the temperature effect in Ni‐based systems is more concentrated in the unique chain growth behavior [256]. Phosphate‐derived Ni catalysts can produce hydrocarbons up to C_4_, and the product distribution follows a chain growth mechanism similar to that of thermal catalytic Fischer–Tropsch (consistent with the Anderson–Flory distribution). While increasing temperature enhanced CO_2_RR activity, it also accelerated the formation of surface carbon (graphite‐like coke), blocking active sites and impairing carbon chain extension. Increasing potential enhanced reduction rates, but also reduced chain growth probability. Additionally, electrolyte cations (e.g., K^+^) significantly promoted the formation of long‐chain products, while anions regulated the rate through a proton supply pathway. When CO was used as a reactant, the probability of chain growth was higher, suggesting that hydrogenation may be the rate‐determining step. Thus, in the Ni system, temperature control and coking effects mutually constrain each other, and optimization of other aspects is necessary to achieve efficient synthesis of long‐chain products.

In the atomic‐level catalytic system, atomically precise Au cluster catalysts provide another perspective, namely the regulatory effect of temperature‐induced interfacial interactions on atomic‐level catalytic performance [257]. The Au_25_(SR)_18_ cluster, due to the hydrophobic ligands, formed a unique solid–liquid–gas three‐phase interface, allowing CO_2_ to rapidly diffuse in the interfacial gaps and generate a local gas‐phase layer, thereby effectively overcoming the limitation caused by the decrease in CO_2_ solubility at elevated temperatures. Theoretical simulations exhibited (Figure 20d) that temperature not only enhances CO_2_ activation, but also regulates proton transfer of *COOH and *CO intermediates by perturbing the hydrogen‐bonding network of the interfacial water layer, thus promoting CO generation. Under the condition of 330 K, the FE_CO_ of Au_25_ clusters could reach about 93.45%, with a partial current density of 54.5 mA cm^−2^ at −0.97 V, approximately twice that at room temperature, while the TOF reached its peak value. This demonstrated that the reaction rate was significantly accelerated with increasing temperature. Further in situ ATR‐SEIRAS experiments confirmed the potential‐dependent adsorption behavior of *COOH and *CO intermediates. However, at 350 K, the excessively disturbed water layer structure disrupted the hydrogen‐bonding network, leading to performance degradation and even catalyst deactivation. This result highlights the synergistic effect of the thermal field and interfacial structure, indicating that temperature not only accelerates reaction kinetics but may also induce deactivation by destroying the interfacial water structure.

At a scale closer to industrial application, non‐isothermal operation strategies further expand the means of thermal field regulation. By constructing a temperature gradient in the MEA with a low‐temperature cathode (20°C) and a high‐temperature anode (60−80°C), Li et al. [258] utilized the Soret effect to drive K^+^ thermodiffusion from the cathode to the anode. Under highly alkaline conditions (1 M KOH), the K^+^ combined with CO_3_^2^^−^/HCO_3_^−^ to form salt precipitation was suppressed, while simultaneously maintaining stability and energy efficiency at high current densities. (Figure 20e,f) Multi‐physics simulations predicted that non‐isothermal operation could increase the critical current density for salt precipitation from 171 mA cm^−2^ to 330 mA cm^−2^ and improve energy efficiency. Experimental verification showed that under non‐isothermal 60°C conditions, the partial current density of CO reached 243.6 mA cm^−2^ with an energy efficiency of 50.6%, which is higher than 47.5% under isothermal 20°C and 17.9% under isothermal 80°C. The MEA could operate stably for more than 200 h without salt precipitation. Techno‐economic analysis indicated that this strategy could reduce the cost of CO production from 0.47 to 0.41 USD kg^−1^, and further down to 0.20 USD kg^−1^ after membrane optimization, demonstrating the high performance and industrialization potential of the non‐isothermal strategy in highly alkaline MEA CO_2_ electrolysis.

Thermal field‐assisted strategies act on the electrocatalytic interface through multi‐layer mechanisms, including regulating intermediate adsorption, reaction kinetics, interfacial water structure, and active site distribution. Fundamental mechanism studies reveal the regulatory patterns of temperature on single‐site catalysts, quantitative analysis identifies the optimal temperature window, and different catalysts exhibit temperature‐sensitive differences in product distribution. Ultimately, non‐isothermal engineering strategies are employed to achieve high‐performance industrial applications. Overall, temperature, as an external control parameter, provides a systematic mechanistic basis and practical guidance for optimizing product selectivity in CO_2_RR. In the future, if thermal fields can be synergistically coupled with electric fields, mass transport, and interface design, precise control of CO_2_RR across all scales and dimensions may be achieved, laying a solid foundation for the transition of carbon conversion from the laboratory to sustainable industrialization.

Under ambient conditions, the solubility of CO_2_ in aqueous media is inherently limited, and increasing the temperature further reduces its solubility, which is detrimental to eCO_2_RR. Although elevated temperatures can accelerate reaction kinetics, they may also intensify the HER, compromising target product selectivity. As an externally adjustable parameter, pressure can act synergistically with temperature and exert a pronounced influence on the reaction microenvironment [259]. According to Henry's law, increasing system pressure markedly enhances CO_2_ solubility [260]. For instance, at ambient pressure (∼1 bar), the solubility of CO_2_ in water is about 0.03 M, whereas raising the pressure to 50 bar increases the solubility to about 1.16 M [261, 262]. This higher dissolved CO_2_ concentration increases *CO coverage on the catalyst surface, facilitating C−C coupling and the formation of multi‐carbon products. Meanwhile, high pressure helps maintain sufficient CO_2_ activity and liquid‐phase stability at elevated temperatures, making temperature−pressure co‐regulation a practical strategy for improving selectivity and energy efficiency under industrially relevant conditions.

Several studies have directly demonstrated the significant contribution of pressure to CO_2_RR performance. For example, a submicron‐thick films rich in (111)‐oriented Cu_2_O nanoparticles anchored on Cu (SW‐Cu_2_O/Cu) prepared by square‐wave electrochemical redox cycling on high‐purity Cu foil, (111)‐oriented Cu_2_O nanoparticle film, achieved nearly 98% FE_formate_ in KHCO_3_ under ≥45 atm CO_2_, while maintaining stable selectivity during ∼20 h of high‐pressure electrolysis [263]. When this cathode was combined with a newly developed NiFe hydroxide carbonate anode in KOH/borate anolyte, the full cell reached a FE_formate_ of 92.7% and an energy efficiency of 55.8% at 60 atm and 6 mA cm^−2^. After 20 h of working, the full‐cell voltage remained stable at ∼2.37 V. The system produced practical quantities of formate (∼0.4 g cm^−2^) without additional product separation. Another study employing a high‐pressure MEA configuration directly electrolyzed CO_2_ captured in the gas phase under high pressure [264]. At room temperature and under 20 bar, an 85% Faradaic efficiency for C_2_H_4_ and a partial current density of 750 mA cm^−2^ were achieved on the In/Cu catalyst. Operando Raman spectroscopy and theoretical calculations indicated that high pressure enriches local CO_2_ coverage and alters the adsorption configuration of *CO, thus promoting C−C coupling. High pressure also shifted bicarbonate formation from inside the GDL to the catalyst−membrane interface, significantly alleviating salt precipitation. This improvement allowed the system to operate stably for more than 1500 h at 600 mA cm^−2^ and 20 bar, while maintaining a C_2_H_4_ FE above 80%. At ambient pressure, by contrast, the C_2_H_4_ FE fell below 11.8% after just 100 h. Direct utilization of high‐pressure captured CO_2_ without depressurization and repressurization, greatly reduces energy consumption and enhances the overall economic viability of CO_2_ utilization. In addition, Koper and co‐workers investigated the effects of temperature and pressure on CO_2_RR at gold cathodes using a custom rotating disk electrolyzer [265]. By increasing system pressure, they effectively compensated for the loss in CO_2_ solubility caused by elevated temperature, thereby maintaining sufficient CO_2_ activity and improving the FE for CO production. At room temperature, raising the pressure to 6 bar increased the FE_CO_ from 42% to 86%. When the temperature was increased to 65°C, the current density increased by an order of magnitude, and the FE_CO_ (∼90%) was preserved.

Collectively, these results demonstrate that pressure not only offsets the reduced CO_2_ solubility at high temperatures but also plays a crucial role in establishing high CO_2_ activity interfaces, promoting C−C coupling, enhancing device stability, and enabling industrial operation. These insights provide clear design guidelines for future high‐temperature, high‐current‐density CO_2_ electroreduction systems.

### 4.2 Light Field‐Assisted eCO_2_RR

Light field modulation exhibits multi‐level mechanisms of action in CO_2_RR: in different systems, it not only provides high‐energy electrons to directly accelerate reactions through the localized surface plasmon resonance (LSPR) effect, but also extends the lifetime of charge carriers in organic frameworks through photosensitive groups, achieves selective switching of product pathways through defect and conformation engineering, and even couples with chiral spin effects to endow the system with new degrees of freedom. Existing research has revealed a gradual evolution from kinetic enhancement to selective programmability to multi‐field synergy with the assistance of light fields.

**FIGURE 21:**
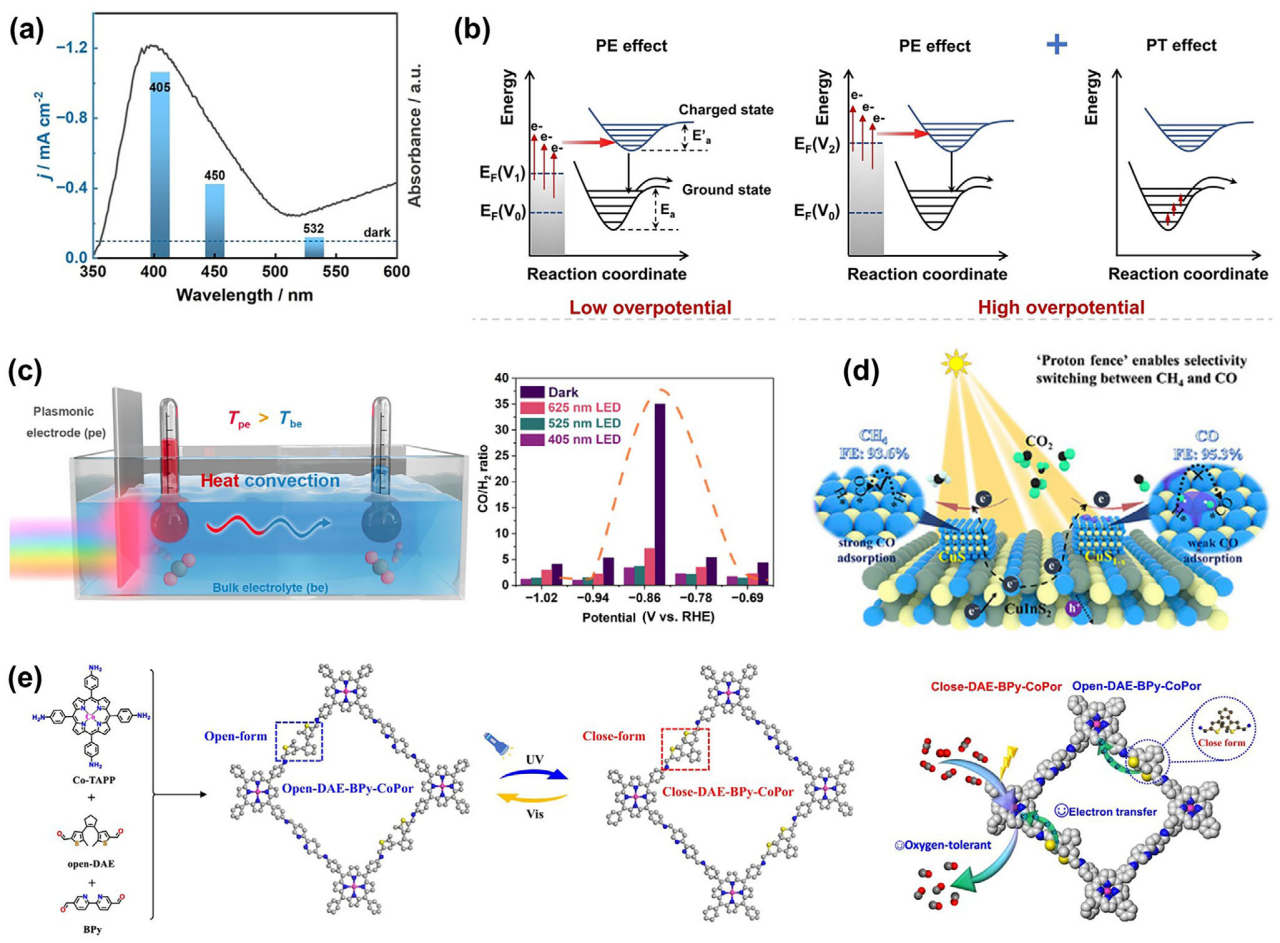
(a) Current density dependence of CO_2_RR on wavelength at −0.6 V. (black: Ag NPs UV/Vis absorption, blue dashed: dark conditions); (b) Plasmonic effects on plasmon‐enhanced CO_2_RR at different potentials. Reproduced with permission [266]. Copyright 2024, Wiley‐VCH GmbH. (c) Schematic of plasma‐enhanced CO_2_ electrolysis and CO/H_2_ ratio on Ag. Reproduced with permission [267]. Copyright 2025, The Author(s). Published by Tsinghua University Press. (d) Schematic of one‐bond switching proton fence effect. Reproduced with permission [270]. Copyright 2025, Wiley‐VCH GmbH. (e) Synthesis routes for open‐DAE‐BPy‐CoPor and close‐DAE‐BPy‐CoPor, along with a schematic diagram illustrating the eCO_2_RR mechanism on close‐DAE‐BPy‐CoPor under aerobic conditions. Reproduced with permission [271]. Copyright 2024, The Author(s).

The most fundamental light field effect was reflected in the direct contribution of localized surface plasmon resonance (LSPR) to electron dynamics [266]. Uniform silver nanoparticles (diameter approximately 50 nm) with localized LSPR effects exhibited significantly enhanced CO_2_RR activity under 405 nm illumination (Figure 21a). Within the potential range of −0.6 to −1.2 V, the current density and FE for CO generation significantly increased, with the onset potential shifting positively by approximately 0.3 V. Mechanistic studies (time‐scale analysis of the photocurrent response) (Figure 21b) indicated that the high‐energy carriers generated by LSPR attenuation (photoelectronic (PE) effect) and the localized photothermal effect (photothermal (PT) effect) contribute differently to the reaction. The PE effect dominated CO_2_RR, promoting CO generation and lowering the reaction energy barrier, while the PT effect promoted HER. In situ ATR‐SEIRAS further revealed that the signal intensity of the *CO intermediate (CO_B_ band) increased under illumination and was positively correlated with CO yield, confirming that the carbonyl intermediate participated in the photoelectron‐driven CO_2_ reduction process. Building on this, studies on silver triangle nanodisks (Ag‐TN) further revealed the regulatory role of LSPR on product ratios [267]. Under different wavelengths (625, 525, 405 nm), the CO/H_2_ ratio can be continuously adjusted from 35 to 1, and increased light intensity leads to a linear increase in photocurrent (Figure 21c). DFT and in situ SEIRAS consistently revealed that blue‐shifting the excitation wavelength gradually reduces the H_2_ formation energy barrier and increases the *COOH formation energy barrier, thereby altering the competitive relationship between CO_2_RR and HER and achieving selective regulation of the CO/H_2_ ratio. Transient absorption spectroscopy correlated higher photocurrent with longer electron–phonon coupling times, demonstrating that the generation and utilization of longer‐lived hot electrons are key to enhancing dynamics. After decomposing the total photocurrent into fast‐response (hot electrons) and slow‐response (thermal effects) components, it was found that the hot electron contribution accounted for over 80% of the total photocurrent at all potentials, indicating that the enhancement of selectivity and activity in this process primarily originates from hot electrons generated by LSPR rather than thermal effects. This suggests that the light field not only enhances activity but also serves as a precise regulator to fine‐tune the competitive relationship between CO_2_RR and HER.

Beyond metallic systems, photonic strategies are increasingly being extended to organic framework materials. Yang and colleagues [268] designed a 3D “saddle‐shaped” covalent organic framework (TP‐TDA‐COF) incorporating electron‐deficient boronate esters and photosensitive thieno*3,2*‐bthiophene groups, achieving highly efficient photochemical coupling for CO_2_RR. At −0.8 V_RHE_, FE_CO_ reached 95.9%, and within the potential range of −0.6 to −1.0 V_RHE_, $FE_{C_{1}}$ exceeded 90%. Under illumination, $j_{C_{1}}$ reached 26.47 mA cm^−2^. TOF_CO_ reached 1842 h^−1^ at −1.2 V, significantly higher than the control system. This was attributed to the photosensitive group enhancing the efficiency of photoinduced electron transfer under light irradiation, while the electron‐deficient site of the borate ester assisted in lowering the activation energy barrier for *COOH formation (from 1.78 eV to 0.82 eV), thereby accelerating the generation of the key intermediates *COOH and *CO. In situ spectroscopy revealed the formation of *HCO_3_^−^, *B‐COOH complexes, and the *CO intermediate within 15 min of illumination. By anchoring the photosensitizer Ru(bpy)_3_Cl_2_ onto a cobalt porphyrin‐based covalent organic framework (Co‐porphyrin‐based COF; Co‐Bpy‐COF) functionalized with *2,2′*‐bipyridine, the constructed Co‐Bpy‐COF‐Ru*_x_* (*x* = 1/2; 2/3) achieved similar results [269]. Ru(bpy)_3_Cl_2_ serves as a strong donor with a long‐lived excited state and a significant built‐in electric field, efficiently transferring excited electrons to the cobalt porphyrin active sites, thereby prolonging their excited state lifetime. This reduced the formation energy barrier for *COOH (the rate‐determining step, RDS) from 1.16 to 0.25 eV, accelerating the CO generation process. The optimal catalyst Co‐Bpy‐COF‐Ru_1/2_ achieved a CO FE of 96.7% at −0.7 V_RHE_ and a CO current density of 16.27 mA cm^−2^ at −1.1 V_RHE_, and a TOF of 933 h^−1^ under illumination, all of which significantly exceeded its performance under dark conditions. This demonstrates that the light field can achieve highly selective CO production by extending carrier lifetime and lowering the energy barrier of key intermediates within the molecular framework.

When light fields are combined with structural engineering, their role expands from enhancing dynamics to dynamically controlling product distribution and stability. By introducing sulfur vacancies (S_v_) into CuS and applying light‐assisted stimulation, efficient “one‐click switching” between CO and CH_4_ products could be achieved [270]. In the complete CuInS_2_/CuS system, a CH_4_ FE of 93.6% was obtained at −0.8 V_RHE_. In the CuInS_2_/CuS_1−_ *_x_* system containing sulfur vacancies, the main product could be converted to CO at just −0.6 V, with a FE as high as 95.3%. Mechanistic studies suggested the “proton fence effect” in the catalytic process (Figure 21d). Sulfur vacancies could adsorb *OH generated by water dissociation in situ, obstructing local proton migration, inhibiting the further protonation of *CO to *CHO, forcing the reaction to remain at *CO and desorb as CO; they also weakened the adsorption energy of Cu sites for *CO (from −0.36 eV to +0.15 eV), thereby significantly promoting CO production. Meanwhile, the photonic field effect enhanced electron–hole separation through the built‐in electric field within the heterojunction and suppressed the reduction of Cu^2+^ to Cu^+^/Cu^0^ by photogenerated holes, thereby maintaining the structural stability of the catalyst, reducing the reduction overpotential by 0.21 V, and improving the overall energy efficiency by 1.8 times. Combining the light field with spin‐polarized catalysts is another approach worth exploring. Lee et al. [272] introduced chiral structures into copper‐based catalysts, developing a highly spin‐polarized L‐DEA Cu_2_O/Cu catalyst with a unique flower‐like spiral surface morphology (spin‐polarization efficiency: 91.7%). In eCO_2_RR, it achieved a current density of −140 mA cm^−2^, with FE_CO_ exceeding 80% and H_2_ FE only 1.6%. In situ Raman spectroscopy and DFT indicated that the chiral structure promotes *COOH adsorption and suppresses H_2_/HCOOH generation through spin control effects. When integrated into a bias‐free photo‐driven CO_2_ conversion system, the solar‐to‐chemical energy conversion efficiency reached 6.37% (with CO accounting for 4.49%), and the system operated continuously and stably for 14 h. This demonstrates that the chiral catalyst not only efficiently and selectively produces CO under electrochemical conditions but can also be applied to sustainable photo‐driven CO_2_ conversion.

Light fields not only enhance CO_2_RR but also address challenges in complex environments. For instance, to address the issues of low selectivity and energy efficiency in eCO_2_RR under real‐world oxygen‐containing CO_2_ source conditions, Huang's team incorporated the light‐switchable molecule diphenyl ethylene (DAE) into a cobalt porphyrin‐based covalent organic framework (CoPor‐COF), constructing open‐type DAE‐BPy‐CoPor and closed‐type DAE‐BPy‐CoPor [271]. Through light‐induced conformation regulation of opening and closing, oxygen passivation and CO_2_ activation were achieved, thereby suppressing oxygen reduction reaction competition (Figure 21e). Unlike processes where light directly participates in the catalytic process, this process was realized by UV light irradiation to induce the catalyst's configuration (i.e., open and closed forms), enabling dynamic regulation under oxygen‐containing conditions. After UV irradiation, the closed‐type DAE‐BPy‐CoPor acquired enhanced oxygen passivation capability and electronic conductivity. Under gas conditions of CO_2_ supply and 5% O_2_, FE_CO_ reached 90.5% at −1.0 V_RHE_, which was nearly 3.5 times and 1.2 times higher than BPy‐CoPor (25.9%) and the open‐type (74.9%), respectively. Additionally, j_CO_ reached 20.1 mA cm^−2^, surpassing BPy‐CoPor (−7.36 mA cm^−2^) and open DAE‐BPy‐CoPor (−15.4 mA cm^−2^). This work not only provides a new strategy for efficient CO_2_RR in oxygen‐containing environments but also offers a feasible approach for designing oxygen‐resistant, high‐electron‐transfer COF catalysts.

Light‐field‐assisted strategies have evolved from their initial focus on simply enhancing reaction rates to more profound control over reaction dynamics. Light fields not only alter carrier dynamics but also introduce new degrees of selectivity by modulating orbital symmetry and spin states. As a result, catalysts are no longer static collections of active sites but are increasingly evolving into reaction platforms that can be dynamically edited by external fields in real time. This transformation signifies that the mission of this strategy is not merely to enhance efficiency but to drive catalytic science from the “structure–performance” paradigm toward a new framework harmonizing “external field‐quantum state‐reaction pathway”. In the future, if light fields can be synergistically regulated with multiple factors such as defects, spin, and electric fields, light‐field‐assisted eCO_2_RR is expected to transition from laboratory exploration to systematic practical applications, thereby opening up new reaction rules for the entire field of catalytic science.

### 4.3 Magnetic Field‐Assisted eCO_2_RR

Although various external field strategies, such as electric fields, light fields, and thermal fields, have been widely used to regulate CO_2_RR, how to simultaneously enhance activity and selectivity without increasing energy consumption remains an unsolved problem. Especially under acidic conditions, competitive HER, concentration polarization, and high free energy barriers at rate‐determining steps often limit the energy efficiency and product distribution of the system. Against this backdrop, magnetic fields have increasingly drawn researchers’ attention due to their unique properties of being non‐contact, controllable, and capable of cross‐scale effects. Unlike other external fields, magnetic fields can drive electrolyte convection through the Lorentz force to alleviate local concentration gradients, reshape the electronic spin states of catalytic centers at the atomic scale, and even simultaneously inject thermal energy and electrical free energy under alternating conditions. These characteristics make magnetic fields not merely supplementary but a new pathway for CO_2_RR that balances macro‐scale mass transfer and micro‐scale electronic structure regulation.

**FIGURE 22:**
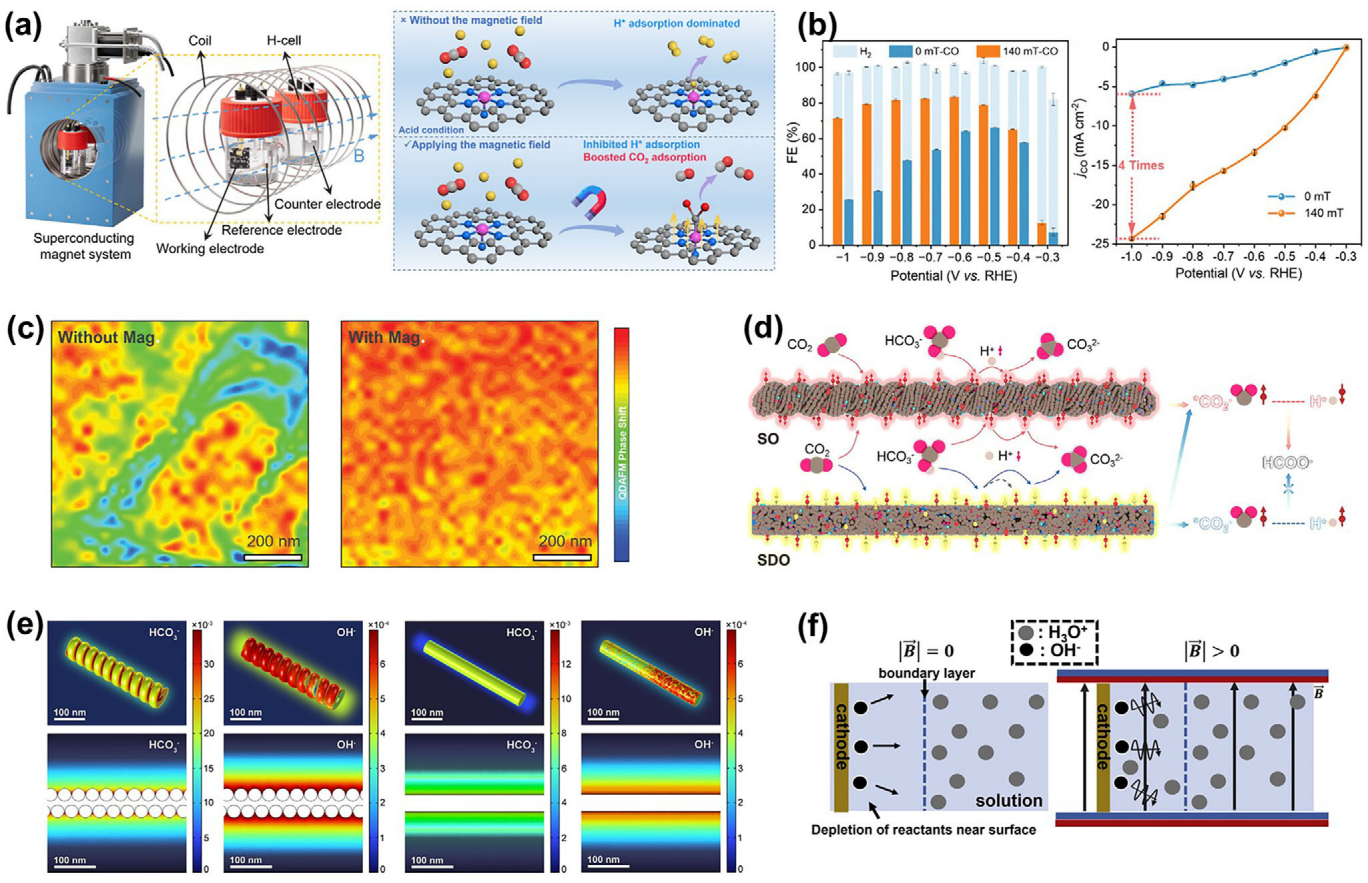
(a) ECO_2_RR device under magnetic field (left) and schematic of magnetic field‐enhanced process (right) (Ni: rose red, N: blue, C: gray, O: red, H: yellow). Reproduced with permission [273]. Copyright 2025, American Chemical Society. (b) FE and j_CO_ of Ni@NC under different external potentials; (c) QDAFM images of Ni@NC in the absence (left) and presence (right) of a magnetic field. (Red denotes magnetic domains; blue denotes magnetic walls.) Reproduced with permission [274]. Copyright 2024, Wiley‐VCH GmbH. (d) Schematic of magnetic field effect on formate production; (e) FEM simulation of surface diffused HCO_3_^−^ and OH^−^ distribution on helical structure. Reproduced with permission [275]. Copyright 2025, Published by Elsevier B.V. and Science Press. (f) Schematic of magnetic field effect on CO_2_ electrocatalytic reduction (CDR). Reproduced with permission [276]. Copyright 2022, Published by Elsevier Inc.

At the microscopic level, magnetic fields can directly modulate the electronic structure and spin states of catalytic sites, thereby enhancing the adsorption and activation of CO_2_. Song et al. [273] demonstrated the enhanced effect of Ni−N_5_−C single‐atom catalysts under high magnetic fields (2 T) (Figure 22a). Applying a 2T magnetic field boosted the FE of CO from 18% to 63.2%. In MEA testing, the FE jumped from 44.0% to 93.7% at a current density of 100 mA, and magnetic field switching experiments showed that this effect is reversible and maintains high selectivity even after multiple cycles. In situ XAS, EPR, and DFT studies elucidated that the external magnetic field enhances the atomic magnetic moment (AMM) of Ni atoms, shortens the Ni−N bond length, and elevates the spin state of Ni 3d orbital electrons, thereby promoting CO_2_ molecule adsorption, bending, and activation, reducing the free energy of the rate‐limiting step, and suppressing the HER, significantly improving kinetics and selectivity. Electrochemical impedance spectroscopy shows that the magnetic field also reduces charge transfer resistance, further accelerating the reaction rate. Thanks to the high FE and reduced cell voltage, the cost of CO production under magnetic field assistance remains lower than under conventional conditions, even when considering the cost of magnetic field facilities. Similar ferromagnetic systems have also been investigated. A novel ferromagnetic Ni@NC electrocatalyst with a sea urchin‐like structure achieved a current density four times higher than that under no magnetic field conditions when a 140 mT external magnetic field was applied in an H‐type electrolyzer (Figure 22b) [274]. In a flow cell, this catalyst achieved nearly 100% FE_CO_ within the potential range of −0.3 to −1.1 V, with minimal overpotential. Quantum diamond atomic force microscopy (QDAFM) revealed (Figure 22c) that the external magnetic field induces the magnetic moments of Ni@NC to transition from disordered multi‐domain to ordered single‐domain structures, eliminating magnetic domain walls and significantly enhancing electronic transport efficiency. DFT analysis then demonstrated that the magnetic field promotes the formation of Ni−O−Ni structures, enabling the oxygen atom of the COOH* intermediate to form stable chemical bonds with two Ni atoms, thereby lowering the energy barrier of the intermediate and accelerating the kinetic process.

Unlike the catalytic systems mentioned earlier, Gu's team chose to analyze the unique role of magnetic fields in non‐ferromagnetic single‐atom systems [275]. They constructed a catalytic system with spin‐polarized properties by anchoring Cu‐N_2_O_2_ single‐atom sites (Cu‐N_2_O_2_/HCNT) on helical carbon nanotubes (HCNT). The electrostatic potential polarization effect of HCNT selectively enriched HCO_3_^−^/OH^−^ near the site while repelling H^+^, suppressing HER and creating a microenvironment rich in anions for the conversion of *CO_2_^−^ to *HCOO^−^; simultaneously, the twisted structure of HCNT induces the Cu site to adopt a high‐spin state, which is further enhanced under an external magnetic field (200 mT), thereby driving the spin ordering of intermediates such as *CO_2_^−^ and *HCOO^−^, significantly improving the efficiency of electron antiparallel pairing (Figure 22d,e). As a result, Cu‐N_2_O_2_/HCNT achieved 93.6% formate selectivity at −0.8 V_RHE_ and maintained >80% selectivity and stable operation for 100 h at a high current density of −175 mA cm^−2^, far exceeding the non‐helical control sample (FE approximately 76.7% and almost no response to the magnetic field). This work expands the magnetic field‐assisted enhancement strategy for single‐atom catalysts, revealing a dual mechanism involving spin polarization and optimization of the local ionic environment, and provides new insights into magnetic field‐assisted enhancement for non‐ferromagnetic catalysts.

In addition to regulating microelectronics, magnetic fields can also improve mass transfer and reactant distribution in electrolytes through magnetohydrodynamics (MHD) effects. A strategy proposed and validated by Whitesides’ team was to enhance mass transfer and selectivity in eCO_2_RR by applying a magnetic field orthogonal to the ionic current to generate Lorentz force‐driven MHD convection (Figure 22f) [276]. In the experiment, an NdFeB permanent magnet was placed vertically on a copper foil electrode to generate a magnetic field. Helical currents within bubbles were observed, and particle‐imaging velocimetry (PIV) was used to quantitatively confirm a significant increase in turbulence near the electrode. Confocal Raman microscopy revealed an increase in pH near the electrode at a 0.3 T magnetic field, indicating that convection weakened the surface pH gradient. In amperometric measurements, changes in current density caused by the magnetic field (determined by the duty cycle) were observed by rapidly placing/removing the magnet (switching time scale ≈ 1 s). Compared to the no‐field/no‐stirring system, the current density increased by approximately 1.3 times under magnetic field conditions, and the selectivity of CO_2_RR relative to HER improved by approximately 2.5 times. Mechanical stirring was more effective for smooth copper foil, while applying a magnetic field was more beneficial for enhancing current density in high‐roughness/high‐ECSA electrodes such as CuO nanowires and foam copper. Combining GDE testing in a flow cell showed that applying a 0.2 T magnetic field at a flow rate of 7 mL·min^−1^ increased the bias current density by approximately 50%, indicating that MHD can be compatible with GDE or mechanical stirring to mitigate concentration polarization. This macro‐level strategy complements micro‐level regulation, with the former optimizing the electronic structure and spin of individual sites, and the latter enhancing overall mass transfer and reaction efficiency through fluid dynamics, achieving synergistic enhancement across multiple scales.

Magnetic field‐assisted strategies demonstrate multi‐dimensional synergistic potential in CO_2_RR and related catalytic systems. From the spin regulation and electronic structure optimization of single atomic sites at the microscopic level, to the magnetic domain rearrangement and intermediate stabilization of nanomagnetic particles, to the enhanced magnetohydrodynamics and mass transfer at the macroscopic scale, magnetic fields can simultaneously intervene in the reaction process at different levels, achieving synergistic optimization of “electronics–structure–mass transfer‐energy”. This cross‐scale synergy not only significantly enhances catalytic activity and selectivity but also reveals the unique value of magnetic fields as an external regulatory tool: they can precisely control the electronic behavior of single‐atom sites, improve the electrolyte environment and reaction kinetics at the macro level, and even achieve efficient catalysis through spin polarization in non‐ferromagnetic systems. In the future, by precisely designing magnetic structures, external magnetic field parameters, and carrier geometric characteristics, the performance of catalysts can be controllably tuned, thereby providing new paradigms and guidance for other complex electrocatalytic processes.

External field‐assisted regulation provides a dynamic optimization approach for eCO_2_RR systems that transcends traditional static designs. Unlike the microscopic environment constructed through the “endogenous” interaction between catalyst structure and electrolyte environment, thermal fields, light fields, and magnetic fields act as “exogenous driving forces” capable of injecting additional energy in real‐time during the reaction process. This breaks the uniformity and inertia of interface conditions, enabling directed adjustment of reaction pathways and dynamic stabilization of intermediates. Thermal fields regulate activation energy barriers and mass transfer through localized heating, optical fields reconfigure interfacial charge distributions using non‐equilibrium electrons and transient polarization effects, and magnetic fields introduce new reaction degrees of freedom via spin selectivity and electromagnetic induction effects. Although the three fields have distinct physical essences, their common value lies in providing a dynamic control framework for eCO_2_RR systems that transcends the limitations of static design. However, external field regulation remains in the exploratory stage, with several challenges that require urgent breakthroughs. First, the spatiotemporal resolution mechanisms of external field effects have not yet been fully elucidated. At the nanoscale interface, how external fields precisely act on specific intermediates, solvent layers, or localized charge distributions requires stronger in situ/real‐time characterization and multiscale simulations for further analysis. Second, there may be nonlinear synergistic or competitive relationships between external fields and microenvironmental regulation. How to avoid thermal effects disrupting confined structures, and how to prevent interference between light fields or magnetic fields and electrolyte parameters, are critical issues that must be addressed in system optimization. Finally, from an application perspective, achieving controllability and scalability of external field application while ensuring energy efficiency is key to the practical implementation of external field regulation. Therefore, the future of external field‐assisted regulation lies not in the competition of a single field but in an innovative approach that integrates static microenvironments, dynamic external fields, and intelligent control.

## 5 Application of Artificial Intelligence in eCO_2_RR

**FIGURE 23:**
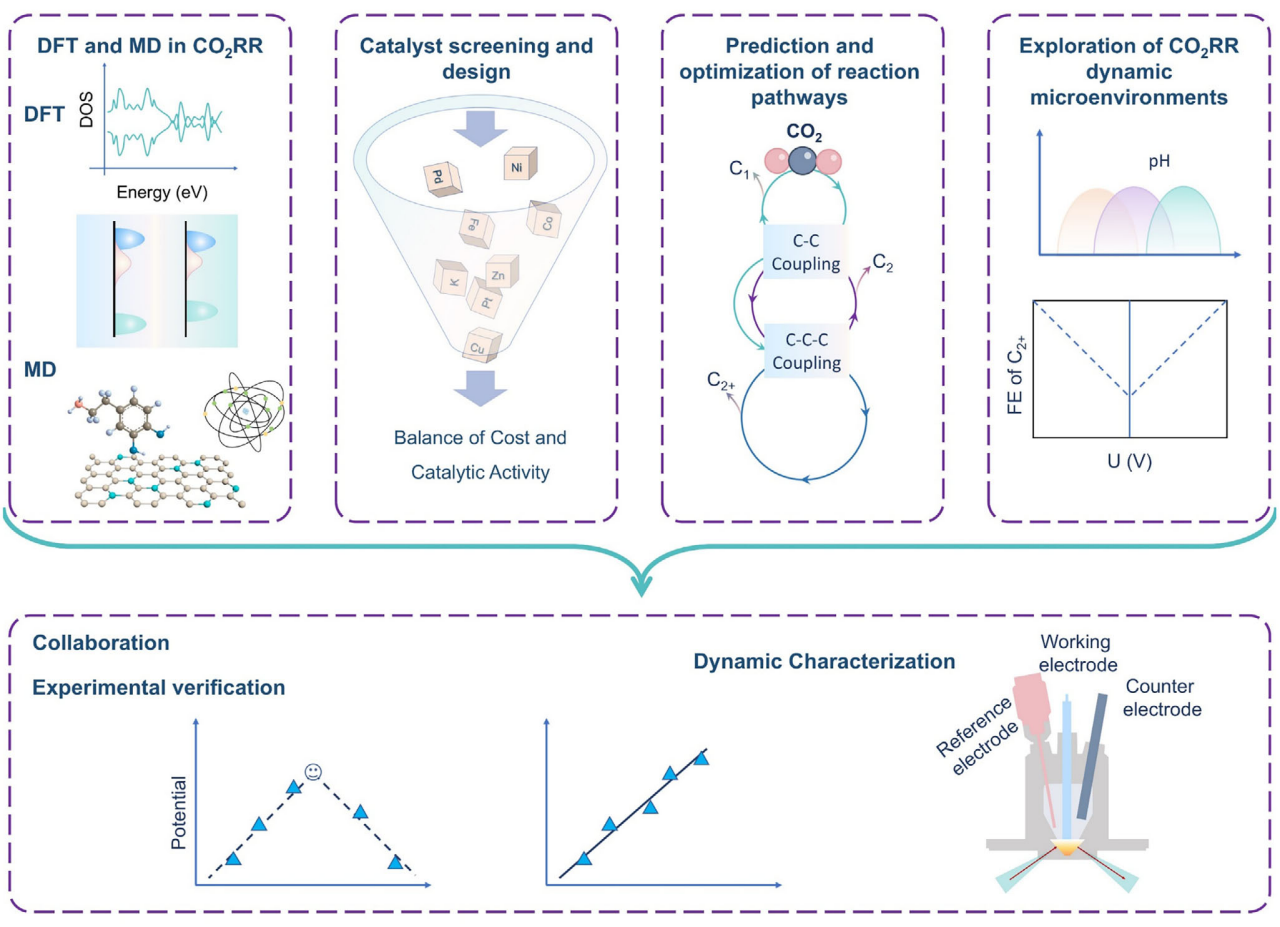
Schematic diagram of the application of AI in eCO_2_RR, including AI‐assisted catalyst screening, reaction pathway prediction, and dynamic micro‐reaction process modelling.

In recent years, theoretical computational methods have played an increasingly critical role in CO_2_RR. Traditional DFT and MD methods provide a solid physical foundation for understanding reaction mechanisms, constructing energy landscapes, and revealing intermediate adsorption behavior. These methods not only help researchers elucidate the mechanisms of active sites on catalyst surfaces but also provide quantitative evidence for the performance differences between different material systems. However, as system complexity and computational scale increase, relying solely on DFT or MD often fails to balance efficiency and accuracy. With the rapid development of AI and ML technologies, they have demonstrated significant application potential in the CO_2_RR field and have been closely integrated with traditional physical modelling methods, injecting new vitality into research in this area [277]. On one hand, AI/ML can assist traditional DFT/MD calculations by constructing efficient potential functions or predictive models, significantly reducing computational time and costs, enabling large‐scale, high‐throughput simulations, and accelerating the screening process for new catalysts. On the other hand, data‐driven AI/ML models can directly extract hidden information from massive experimental and theoretical computational data, learn the complex relationships between catalyst structure and performance, and rapidly predict catalyst activity and selectivity without the need for extensive first‐principles calculations, thereby opening up new avenues for rational catalyst design. This section (Figure 23) will systematically discuss the application of traditional DFT and MD methods in CO_2_RR, focusing on strategies for integrating AI, AI‐assisted catalyst screening, reaction pathway prediction, and dynamic micro‐reaction process modelling.

### 5.1 Application of DFT and MD in eCO_2_RR Mechanism Analysis

**FIGURE 24:**
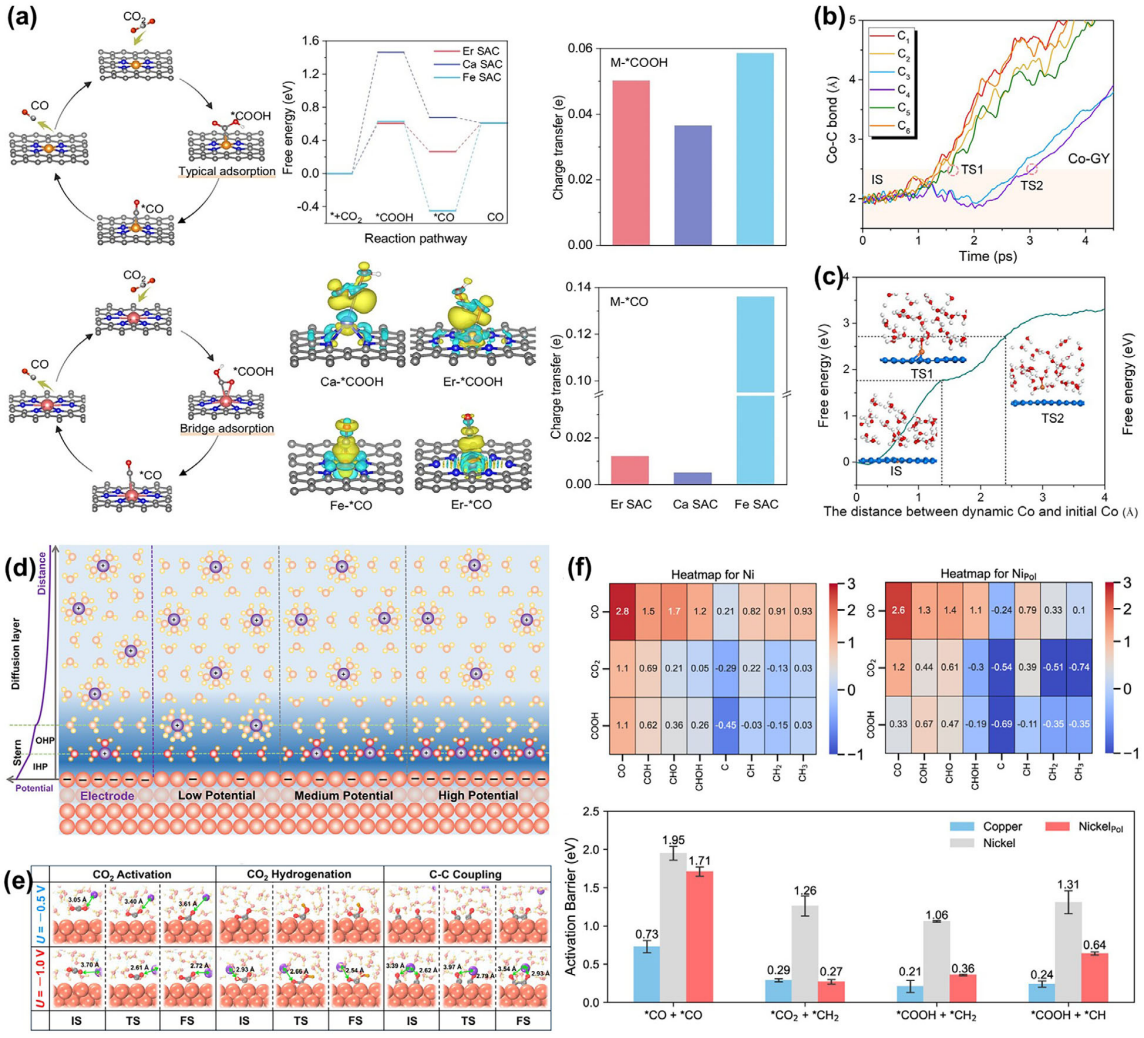
(a) DFT calculations. Reproduced with permission [17]. Copyright 2025, Nature Publishing Group. (b) Dynamic evolution of Co−C*_x_* bond length and (c) free energy change of Co‐GY SAC under 3% tensile strain. Reproduced with permission [278]. Copyright 2024, American Chemical Society. (d) Schematic of the electrical double‐layer (EDL) structure and the distinct K^+^ distribution states; (e) The structures of the initial state (IS), transition state (TS), and final state (FS) of CO_2_ activation/hydrogenation, and C−C coupling. Reproduced with permission [279]. Copyright 2025, American Chemical Society. (f) Heatmap analysis of thermodynamic reaction energies for various C−C coupling patterns on Ni and Ni_pol_. And, kinetic energy barrier analysis of *CO+*CO, *CO_2_+*CH_2_, *COOH+*CH_2_, *COOH+*CH on Cu, Ni, and Ni_pol_. Ni_pol_ means C−C coupling process occurs on Ni^0^–Ni^δ+^ domain. Reproduced with permission [280]. Copyright 2025, American Chemical Society.

Charge density difference and Bader charge analysis are important theoretical tools for studying the electronic structure and bonding nature of catalytic systems. The former can visually demonstrate the redistribution of electrons after the interaction between two fragments, thereby revealing the direction of electron flow and charge transfer behavior; the latter can quantitatively analyze the extent of charge gain or loss during the formation of chemical bonds, providing a solid basis for understanding the interaction between adsorption intermediates and metal centers. Wang et al. [17] used erbium (Er) as a representative catalyst and combined DFT calculations, in situ spectroscopy, and XAS to reveal its unique bridge‐type adsorption mechanism. DFT calculations revealed that the intermediate COOH exhibits bridge‐like adsorption at the Er site, while linear adsorption occurs at the Fe and Ca sites (Figure 24a). This bridge‐like adsorption reduced the activation energy barrier for CO_2_ (0.61 eV). Additionally, the desorption energy of CO on Er SAC was significantly lower than that on Fe SAC, demonstrating an advantage in facilitating product release. Charge difference and Bader charge analysis further confirm that Er SAC exhibited stronger electron transfer toward COOH but weaker toward CO, favoring CO generation and desorption. Furthermore, XAS results showed that Er remained in a single‐atom dispersed state during the reaction. This study not only achieved the universal application of 14 SACs among lanthanide elements but also demonstrated CO Faradaic efficiencies exceeding 90% for all SACs. Among them, Er SAC exhibited a TOF of approximately 130 000 h^−1^ at 500 mA cm^−2^ and achieved a total cell efficiency of 34.7% and a single‐pass conversion efficiency of 70.4% at 200 mA cm^−2^. Lanthanide single‐atom catalysts break the energy barrier coupling between COOH and CO in traditional SACs through a unique bridge‐like adsorption mechanism, providing a new approach for efficient CO_2_ electrocatalytic conversion.

Traditional DFT calculations are typically performed in vacuum or under fixed charge conditions, making it difficult to accurately reflect the potential effects and solvent interactions in actual electrochemical environments. Additionally, the structural stability of catalysts under strain conditions is critical to their practical application, and static DFT alone is insufficient to reveal the time‐dependent evolution process. Therefore, Liu et al. [278] employed constant‐potential DFT calculations and constrained AIMD simulations to systematically investigate the strain‐regulated effects and stability of γ‐graphyne (GY)‐supported single‐atom catalysts (M‐GY SACs) in the two‐electron CO_2_ reduction (2e^−^‐CO_2_RR). DFT results revealed that while Co‐GY exhibited high activity for CO_2_RR in its intrinsic state, its selectivity is limited by HER. Upon applying tensile strain, the charge density at the catalytic center undergoes rearrangement, leading to an increase in the valence state of the Co atom and a subsequent weakening of its binding to adsorbed species. As strain increases, both the CO_2_RR and HER ultimate potential (ULT) shift toward more negative values, but the HER becomes more sensitive to strain changes, increasing the potential difference between the two and thereby enhancing CO_2_RR selectivity. Further constant potential analysis revealed that strain primarily influences the energy changes of COOH and H by regulating the zero‐charge potential (UPZC), with the adsorption energy of *H decreasing more rapidly, becoming the key factor in achieving enhanced selectivity. AIMD simulation results demonstrated that although the Co−C bond gradually elongates over time under strain, complete dissociation requires a free energy exceeding 3.3 eV (Figure 24b,c), indicating that metal atoms are difficult to lose at room temperature. Additionally, H migration to the C site required overcoming an energy barrier of approximately 0.96 eV, making basal hydrogenation difficult; furthermore, the Co site exhibited strong resistance to poisoning by intermediates such as OH and *CO. This study combines constant‐potential DFT and AIMD to reveal the regulatory mechanism of strain on the competitive relationship between CO_2_RR and HER, clearly proposing that strain can serve as a universal and effective means to enhance the selectivity of single‐atom catalysts toward 2e^−^‐CO_2_RR, providing a theoretical basis for the design of strain‐sensitive electrocatalysts. Similarly, combining constant‐potential DFT and constrained AIMD simulations, Fu et al. [279] systematically elucidated the mechanism by which K^+^ distribution at the interface under different potentials influences the competitive relationship between HER and CO_2_RR. It was pointed out (Figure 24d) that at low potentials (−0.5 V), weak electrostatic interactions make it difficult for K^+^ to adsorb on the inner Helmholtz plane (IHP), leading to HER dominance; at moderate potentials (−1.0 V), an appropriate amount of K^+^ is stably distributed on the IHP, which can both stabilize the intermediate state of CO_2_RR and inhibit HER; at high potentials (−2.0 V), excessive aggregation of K^+^ blocks the CO_2_ mass transfer channel, thereby promoting the recurrence of HER. Additionally, they further demonstrated through energy barrier calculations and transition state structure analysis (Figure 24e) that at −1.0 V, K^+^ significantly reduces the coordination stability of the *CO−*CO significantly reduces the energy barrier for C−C coupling, making the rate‐determining step energy barrier for CO_2_RR (0.44 eV) lower than that for HER (0.92 eV), which well explains the experimentally observed CO_2_RR advantage. This study not only explains the long‐standing potential effect observed in experiments but also provides new insights for future efforts to suppress HER and enhance CO_2_RR by regulating interfacial cation concentration under high‐potential conditions.

For a long time, experiments have shown that alkali metal ions in electrolytes can significantly enhance the selectivity of C_2+_ products, but the underlying mechanism remains unclear. To address this, Qin et al. [39] employed constant‐potential AIMD simulations under constant potential and a “slow‐growth” sampling method to construct a rational electrode‐electrolyte interface model. They discovered that K^+^ ions undergo specific adsorption near *CO adsorption sites on the Cu surface and directly participate in the *CO−*CO coupling process. This interaction reduces the coupling energy barrier by approximately 0.20 eV, significantly accelerating the coupling kinetics. Experimental results also corroborated theoretical findings: when K^+^ specific adsorption was suppressed by adding the cationic surfactant CTAB to the electrolyte, the FE of the C^2+^ product decreased from 41.1% to 4.3%. This discovery provides crucial theoretical foundations and practical guidance for regulating interfacial cations to enhance the selectivity of multi‐carbon products in eCO_2_RR. Recently, to address the challenge of efficiently producing C_3+_ long‐chain hydrocarbons in CO_2_RR, Ding et al. [280] employed AIMD combined with the slow‐growth method to investigate the microscopic mechanism by which Ni^0^−Ni^δ+^ domains in Ni‐based catalysts promote carbon chain growth. It was found that by modulating the d‐band center of polarized Ni, the adsorption strengths of CO_2_ and CO can be significantly reduced, thereby mitigating CO poisoning in the catalyst and promoting the formation and stable existence of COOH. Concurrently, CO generated in non‐polarized regions migrates to polarized areas for further hydrogenation into CH*_x_* (*x *= 1, 2). C−C coupling occurs between COOH and CH*_x_*, exhibiting activation energies substantially lower than conventional *CO coupling pathways, resembling the chain‐growth mechanism in Fischer–Tropsch synthesis. They systematically evaluated the coupling reaction energy barriers between various carbon intermediates and CO_2_, COOH, and CO (Figure 24f). The results indicated that CO_2_ and COOH possess greater thermodynamic advantages as building blocks for C−C coupling. Based on this mechanism, they proposed that regulating oxidation to construct M^0^−M^δ+^ structures (M = Fe, Rh, Pd, Co, Ru) centered on d orbitals could also enable efficient C−C coupling on similar metals. Overall, this study reveals the microscopic mechanism by which metal polarization strategies promote long‐chain carbon formation by modulating the thermodynamic properties of intermediates, providing new insights for designing efficient Ni and other metal‐based CO_2_RR catalysts.

Theoretical studies based on DFT and AIMD have provided indispensable atomic‐scale insights into key elementary steps in CO_2_RR, including adsorption behavior, electric‐field effects, interfacial solvation, ion redistribution, and C−C coupling. The discussion in this section illustrates how the nature of electrolyte cations, local hydrophobic/hydrophilic structures, and the spatial electric‐field distribution generated by three‐dimensional electrodes act together to shape the electrocatalytic microenvironment, thereby governing product pathways and associated energy barriers. However, our survey of existing theoretical work also reveals an often overlooked issue: the main limitation of traditional theoretical approaches no longer lies in their accuracy, but rather in their “applicability” to realistic CO_2_RR systems.

### 5.2 AI‐Based Catalyst Screening and Design

**FIGURE 25:**
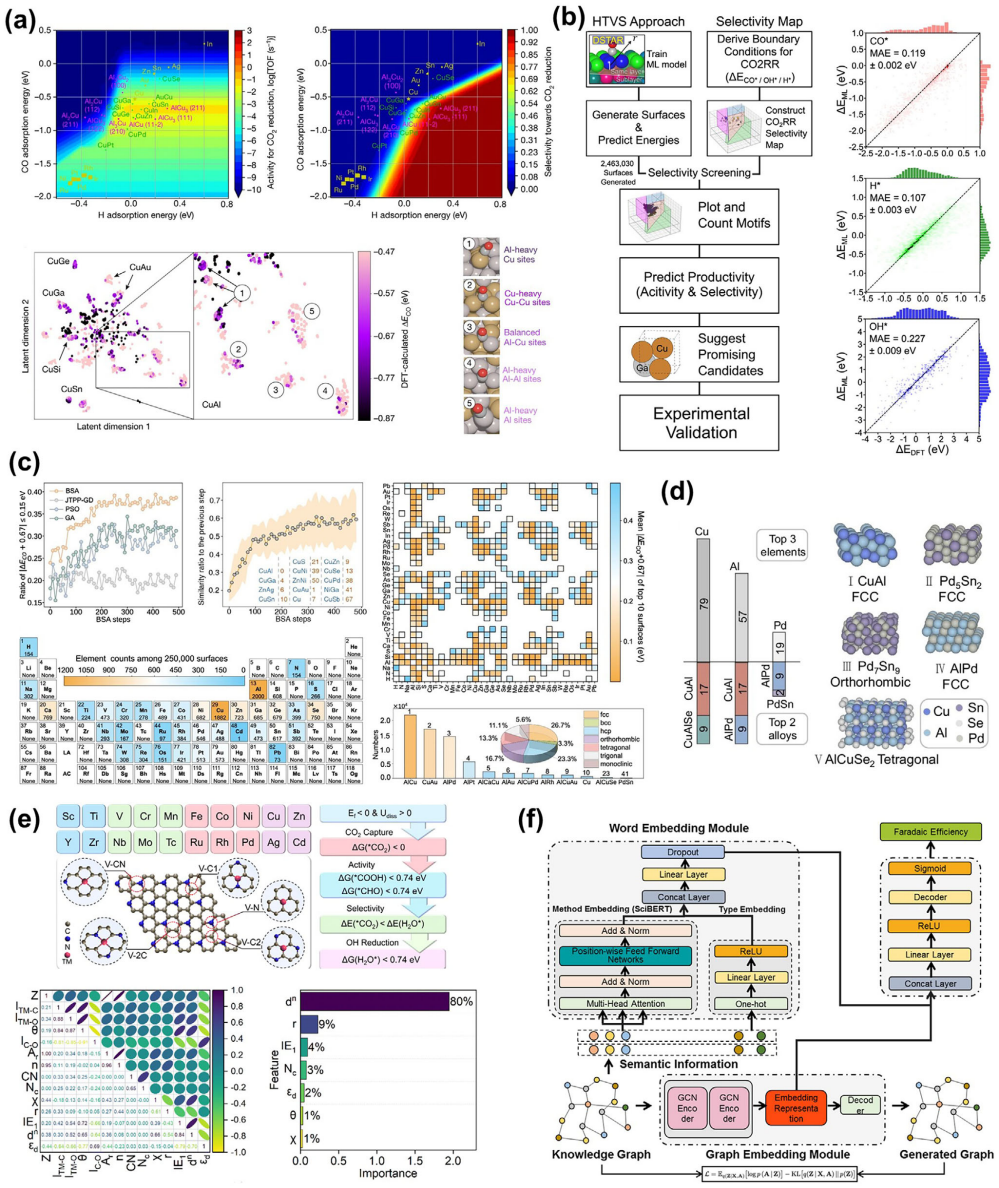
(a) Screening of Cu and Cu based compounds using computational methods. Reproduced with permission [46]. Copyright 2020, Nature Publishing Group. (b) High‐throughput virtual screening schematics and performance of the ML model. Reproduced with permission [281]. Copyright 2023, Nature Publishing Group. (c) Results of inverse design alloy surfaces for CO_2_RR; (d) Top 3 metal elements among 110 surfaces and top 2 alloys of Cu, Al, and Pd‐based surfaces. Reproduced with permission [47]. Copyright 2020, Nature Publishing Group. (e) Schematic of 20 transition metals and five different defects on C_5_N as loading sites along with screening criteria. Reproduced with permission [282]. Copyright 2025, Royal Society of Chemistry. (f) Overall architecture of the proposed framework for Faradaic efficiency prediction. Reproduced with permission [283]. Copyright 2023, American Chemical Society.

Although DFT/AIMD can accurately describe any given instantaneous state, they struggle to span the relevant spatial and temporal scales needed to construct a complete picture of interfacial evolution. In reality, the reaction interface is not the static, single‐layer adsorption structure assumed in conventional models, but a highly dynamic, coupled system: ion clusters continuously exchange, solvent networks undergo persistent rearrangement, electric‐field distributions shift in response to potential changes, and catalyst surfaces reconstruct under operating conditions. As a result, relying solely on first‐principles methods often enables post‐rationalization of experimental phenomena but provides limited predictive power under realistic reaction conditions. This gap has accelerated the rise of AI and ML in CO_2_RR research, where they have begun to demonstrate unique and irreplaceable advantages. These methods retain the physical rigor of first‐principles calculations while extending atomic‐level precision to much larger systems and longer time windows through neural network potential, graph neural networks, and related techniques. Thus, they permit simulations of interfacial dynamics beyond the reach of traditional AIMD. At the same time, AI models can automatically extract hidden variables from combined experimental and theoretical datasets, capturing interfacial behaviors, solvation effects, and many‐body interactions that are difficult to explicitly encode in conventional models. It will drive catalyst design to shift from empirical exploration toward data‐driven and physics‐informed predictive design. For instance, Zhong et al. [46] employed a combined DFT calculation and active machine learning approach to perform high‐throughput screening among thousands of Cu‐based intermetallic compounds, ultimately identifying Cu–Al alloys as the most promising catalysts. Computational results demonstrated that Cu–Al alloys provide active sites with near‐optimal CO adsorption energies (−0.67 eV) across diverse surface structures, balancing high activity and selectivity. As shown in Figure 25a, the volcano plot of activity versus selectivity reveals peak catalytic activity when CO adsorption approaches −0.67 eV. Further analysis via t‐SNE dimensionality reduction maps reveals that the Cu–Al system contains a large number of sites with adsorption energies close to the optimum. Moreover, the proportion of overly strong CO‐binding sites is low, helping to avoid CO poisoning. Both experimentally synthesized ion‐implanted and evaporated‐etched Cu–Al catalysts exhibited outstanding performance, achieving over 80% FE for C_2_H_4_ under flow cell conditions, significantly outperforming pure Cu (∼66%), while maintaining high product selectivity even at a current density of 400 mA cm^−2^. This study demonstrated an effective pathway that combines machine learning and theoretical calculations to accelerate the discovery of novel multimetallic catalysts, providing new insights for CO_2_RR to C_2+_ products.

To address the limitations of exploration space and the difficulty in simultaneously predicting multi‐product selectivity in ML approaches for CO_2_RR catalyst development, Back and his collaborators proposed a high‐throughput virtual screening (HTVS) strategy integrating pre‐trained ML models with CO_2_RR selectivity maps [281]. This approach utilized the structure‐free active motif‐based representation (DSTAR) model for rapid prediction of adsorption energies (ΔE_CO*_, ΔE_OH*_, ΔE_H*_) and combined it with selectivity maps to simultaneously obtain activity and product distribution information (Figure 25b). Employing this strategy, they systematically predicted 465 metal catalysts, identifying two previously underreported alloy categories, Cu–Pd and Cu–Ga, exhibiting outstanding selectivity for C_1+_ products and HCOOH generation, with experimental validation of the predictions. This study proposed a high‐throughput screening method that overcomes database limitations and significantly expands the chemical space, paving a new path for accelerating the discovery of highly efficient and selective catalysts for CO_2_RR.

In material inverse design, traditional generation models are often confined to localized chemical spaces, struggling to efficiently explore the global chemical space. To address this, Song et al. [47] proposed a unified framework called Material Generation with Efficient Global Chemical Space Search (MAGECS), integrating Bird Swarm Algorithm (BSA), Crystal Diffusion Variational Autoencoder (CDVAE), and supervised Graph Neural Networks (GNNs) to achieve target‐performance‐driven material structure generation. In designing CO_2_RR electrocatalysts, MAGECS generated approximately 250 000 alloy surface structures, with the proportion of highly active structures increasing by 2.5 times compared to random generation (Figure 25c). They further screened 110 potentially high‐performance surfaces from 250 000 candidates using metrics including CO adsorption energy, H adsorption energy, and formation energy, validated via DFT calculations, revealing high prediction accuracy with most generated surfaces exhibiting superior activity to training data. Ultimately, they synthesized and characterized five predicted alloys (CuAl, AlPd, Sn_2_Pd_5_, Sn_9_Pd_7_, and CuAlSe_2_), whose bulk structures and surface orientations matched the generated structures (Figure 25d). Among these, CuAl exhibits particularly outstanding performance in producing C_2_ products. This work has proven that MAGECS can efficiently perform inverse design across vast chemical spaces, advancing the intelligent discovery of functional materials. It also indicates potential for future improvements in multi‐objective optimization and synthesizability prediction.

In recent years, single‐atom catalysts (SACs) have emerged as promising candidates for CO_2_RR, but the lack of efficient predictive methods has hindered their systematic exploration. Zhou et al. [282] combined DFT calculations with ML to systematically investigate SACs formed by 3d and 4d transition metal atoms anchored on five C_5_N defect substrates (Figure 25e). They first employed a five‐step rigorous screening strategy to evaluate 100 candidate SACs based on structural stability, CO_2_ adsorption capacity, catalytic activity (CO production and hydrogenation), selectivity, and OH intermediate reduction reactions. This process identified nine stable catalysts exhibiting superior catalytic activity and selectivity compared to conventional Cu(211), with Pd@C_5_N_C2 demonstrating the best performance, with a limiting potential of only 0.42 V. It discovered that in certain systems, the often‐neglected OH reduction step could become a potential decisive step (PDS). By simultaneously coordinating C and O atoms at the active site, a linear energy relationship in the CO‐to‐CHO conversion was broken, thereby lowering the free energy barrier and enhancing catalytic efficiency. Furthermore, utilizing XGBoost and random forest models, they performed ML predictions of reaction energy barriers. Analysis revealed that the number of d electrons (d^n^), first ionization energy (IE_1_), d‐band center (ε_d_), and atomic radius (r) are key parameters determining CO_2_RR performance. This study establishes an efficient, reliable screening strategy. This provides theoretical guidance for rapidly designing and discovering high‐performance single‐atom CO_2_RR catalysts, with potential for extension to other two‐dimensional material catalysts.

Interestingly, a knowledge graph construction and Faraday efficiency prediction method based on the language‐enhanced SciBERT framework was proposed by Gao et al. [283] to rapidly uncover catalyst design patterns while reducing reliance on extensive DFT computational data. They extracted diverse entity information, including material type, regulation method, product, Faraday efficiency, cell configuration, electrolyte, synthesis method, current density, and voltage, from 757 relevant publications. By constructing an interconnected knowledge graph, they visualized the decade‐long evolution of copper‐based catalysts, the relationship between catalytic performance and material properties, and emerging trends in catalyst design. The framework comprises four modules: data preprocessing, named entity recognition, knowledge graph construction, and Faraday efficiency prediction. Subsequently, SciBERT was employed for word embedding of literature, combined with Variational Graph Autoencoder (VGAE) for graph embedding of the knowledge graph. This fusion of linguistic knowledge and structured relational information enabled the prediction of Faraday efficiency for specific materials, achieving superior performance compared to traditional methods (Figure 25f). Results demonstrated that this approach not only reveals activity patterns and design trends for copper‐based electrocatalysts but also provides researchers with references for catalyst selection and optimization, showcasing the potential of integrating artificial intelligence with catalytic science to guide novel material design.

Overall, the unique value of AI and ML in CO_2_RR catalyst screening and materials design can be seen across several aspects: from high‐throughput calculations aided by active learning, to rapid adsorption energy prediction models, generative frameworks for exploring global chemical spaces, and knowledge‐graph‐based extraction of empirical rules. These methods form a closed‐loop design pathway covering broad chemical space exploration, structure generation, performance prediction, and experimental validation. Compared with traditional DFT‐based point‐by‐point calculations, AI significantly enhances exploration efficiency across vast material spaces and uncovers many hidden structure–performance relationships that are difficult to obtain through explicit computations. Examples include multi‐active‐site characteristics, the coupling between structural stability and electronic properties, and cross‐study statistical trends affecting Faradaic efficiency in copper‐based systems. This means that AI has evolved beyond merely being a computational accelerator and is increasingly serving as a knowledge organizer and pattern extractor in the materials discovery process.

### 5.3 AI‐Driven Intelligent Prediction and Optimization of Reaction Pathways

Catalyst design represents only the first step in CO_2_RR research. The actual product distribution and selectivity are determined by complex, multi‐step reaction networks and their corresponding energy landscapes. The role of AI/ML naturally extends from materials screening to the intelligent prediction and optimization of reaction pathways. Beyond catalyst screening, big data and machine learning enable intelligent prediction and optimization of reaction pathways. In complex multi‐step, multi‐intermediate systems like CO_2_RR, ML not only rapidly identifies the lowest‐energy, most favorable reaction routes among numerous possibilities but also reveals competitive relationships between different pathways by analyzing the correlation between intermediate adsorption energies and reaction selectivity. This approach not only accelerates mechanistic elucidation but also provides a holistic optimization strategy for C−C coupling and C_2+_ product formation, advancing the rational design of highly efficient and selective electrocatalysts.

**FIGURE 26:**
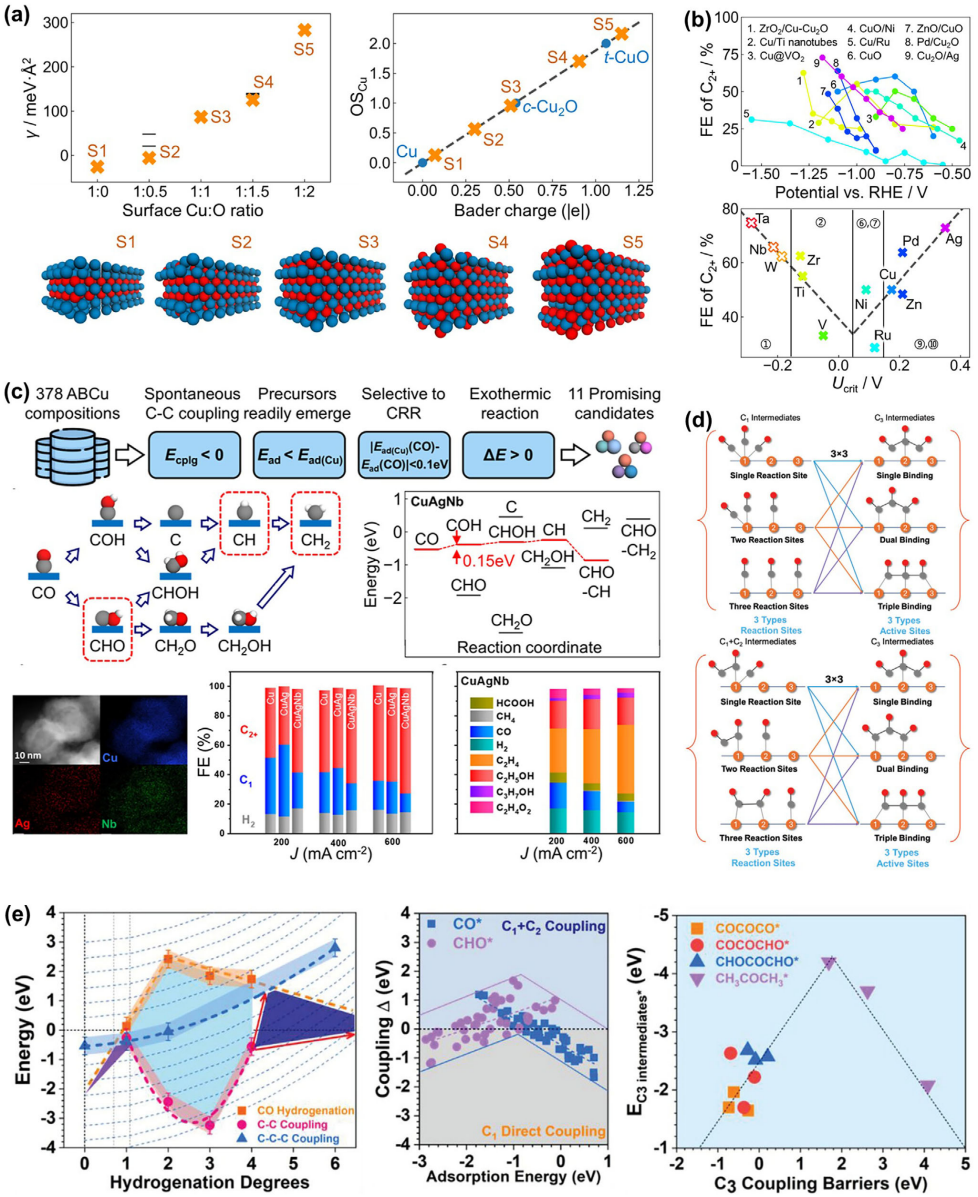
(a) Benchmark of Cu oxidation states (OSCu) across surface structures; (b) Experimental C_2+_ FE for Cu‐based catalysts, and relationship between a selected “best” FE value. Reproduced with permission [284]. Copyright 2023, American Chemical Society. (c) Experimental validation of big data‐predicted C−C coupling catalysts. Reproduced with permission [285]. Copyright 2024, American Chemical Society. (d) C−C−C couplings by C_1_ intermediates and C_1_+C_2_ intermediates; (e) Key correlations governing C−C coupling pathways and reaction barriers, including hydrogenation effects, coupling route competition, volcano relationships. Reproduced with permission [286]. Copyright 2024, The Authors.

At present, it lacks a clear theoretical understanding of the selectivity issue in the formation of the C_2+_ products, and relying solely on the volcano‐shaped relationship between adsorption energy and activity is insufficient to explain the selectivity patterns in complex reaction processes. Therefore, Li et al. [284] first constructed different oxides of copper (OSCu) structures based on the CuO(001) surface model. The stepwise evolution from +0 to +2 revealed the distribution pattern of Cu valence states regulated by surface oxygen content (Figure 26a). Through surface energy calculations, they verified the structural stability and uniform distribution characteristics of OSCu = +0.5, demonstrating its ability to significantly promote the critical C−C coupling step and favor the formation of C_2+_ products. They introduced transition metal doping to regulate the surface oxidation state of Cu. By integrating Pourbaix diagrams, they determined the critical potential for Cu transitioning from +0 to +0.5 under different doping conditions. Based on experimental data and AI clustering results, they discovered an inverted volcano relationship between the FE of C_2+_ products and the critical potential: on the left side of the curve, dopants tend to increase the Cu oxidation state, enhancing C_2+_ selectivity; on the right side, excessive oxidation states inhibit C_2+_ formation (Figure 26b). This pattern not only aligns closely with experimental data but also provides a theoretical basis for predicting outcomes in understudied metal‐doped systems. Subsequently, they constructed a comprehensive dataset exceeding 45 000 data points, encompassing all possible coupling configurations of six precursors and their adsorption conformations on different active sites [285]. They efficiently scaled DFT computational data using iterative sampling and a 2D−3D integrated machine learning strategy. Data analysis revealed that asymmetric coupling (e.g., CHO with CH or CH_2_) outperforms symmetric coupling. Additionally, Cu‐based catalysts demonstrated significantly enhanced C_2+_ selectivity through bimetallic or ternary doping, with CuAgNb exhibiting optimal performance. Figure 26c presents their proposed four catalyst screening criteria (low coupling energy, easy precursor adsorption, exothermic precursor formation, and CO adsorption energy close to Cu to avoid hydrogen evolution). Based on these criteria, 11 potential combinations superior to Cu were identified, with energy analysis confirming the optimal coupling pathways as CH−CHO or CH_2_−CHO. Further experiments validated the CuAgNb catalyst, revealing significantly higher FE for C_2+_ products like ethylene and ethanol compared to Cu and CuAg, alongside markedly reduced CO byproduct yields, which are highly consistent with theoretical predictions. Collectively, Li's works integrate big data with experimentation to uncover key C−C coupling mechanisms while providing an actionable framework for rational CO_2_RR catalyst design.

Similarly, current electrocatalytic research on C_3_ products remains limited, plagued by unclear mechanisms and difficulties in catalyst screening. Sun et al. [286] proposed a first‐principles machine learning (FPML) technique to systematically predict C−C−C coupling processes and C_3_ reaction pathways in graphyne‐supported atomic catalysts (GDY‐ACs). Unlike previous approaches relying solely on extensive density functional theory (DFT) calculations, this study constructed a learning database based on existing C_1_ and C_2_ reaction data, achieving the first direct prediction of C_3_ product pathways. By integrating neighboring effect and the small‐large integrated cycle mechanisms, they examined the competitive dynamics among C_1_, C_2_, and C_3_ pathways (Figure 26d), revealing that C_3_ large‐cycle selectivity is constrained by competition from C_1_ small‐cycle and C_2_ medium‐cycle pathways. Machine learning‐based energy barrier predictions further revealed trends in C−C−C coupling under varying degrees of hydrogenation and summarized correlations between key intermediate adsorption energies and reaction barriers (Figure 26e). Results indicate that direct C_1_ coupling is energetically favored, while C_1_+C_2_ coupling may occur under highly hydrogenated states. Concurrently, distinct rate‐determining step (RDS) energy barrier characteristics were observed across different metal GDY‐ACs, identifying Cu, Mn, Pd, Pt, Pr, and Pm as potential high‐efficiency electrocatalysts for C_3_ products. This work not only proposed an efficient new method for predicting C_3_ pathways but also provides novel insights into mechanism understanding and catalyst screening.

The above framework demonstrates the potential of AI in predicting and optimizing reaction pathways in CO_2_RR. Unlike traditional approaches that rely solely on static adsorption energies and local volcano plots, AI/ML techniques can handle the complex correlations in multi‐step, multi‐intermediate systems, rapidly identifying the energetically most favorable pathways and revealing selectivity patterns for C−C and C−C−C coupling. By integrating high‐throughput DFT data, experimental observations, and big data analysis, researchers can not only predict reaction pathways for different metals or doped systems but also quantify the relationships between hydrogenation levels, intermediate adsorption modes, and final product distributions, achieving a systematic understanding from atomic‐scale mechanisms to macroscopic product selectivity.

### 5.4 AI‐Assisted Exploration of eCO_2_RR Dynamic Microenvironments

It should be noted that the optimization of reaction pathways represents only one part of the overall performance prediction in CO_2_RR. As previously discussed, in CO_2_RR, reaction performance is influenced by multiple factors, including voltage, pH, temperature, solution composition, and catalyst surface state. Traditional experiments or DFT calculations struggle to systematically analyze the coupled effects of all variables. AI‐assisted exploration of the dynamic microenvironment in CO_2_RR not only enables rapid prediction of catalytic performance under different operating conditions but also reveals the complex coupling relationships between voltage, pH, temperature, solution composition, and catalyst surface state. By constructing high‐dimensional data models, machine learning establishes quantitative correlations between microscopic features, such as adsorption energies, electronic structures, and spectral signals obtained from experiments or computations, and macroscopic reaction performance. This enables dynamic predictions of reaction pathways, active site evolution, and side‐reaction competition. Such approaches guide optimizing conditions, enhancing selectivity and stability, thereby achieving intelligent monitoring and regulation of CO_2_RR.

**FIGURE 27:**
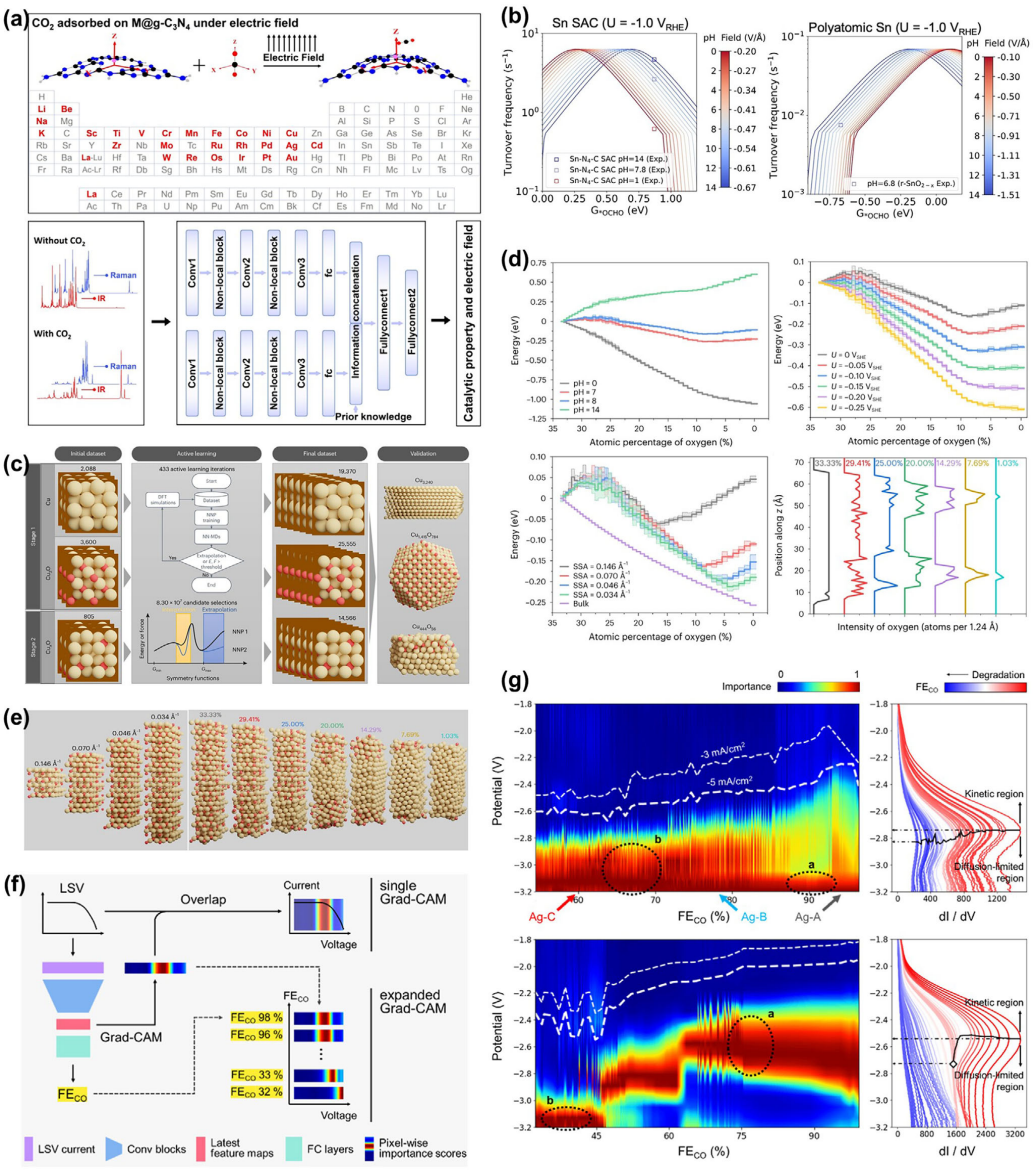
(a) Flowchart for predicting the adsorption energy of M@g‐C_3_N_4_ for CO_2_ through machine learning. Reproduced with permission [287]. Copyright 2023, American Chemical Society. (b) pH‐dependent modelling for CO_2_RR on Sn−N_4_−C SAC and polyatomic Sn. Reproduced with permission [288]. Copyright 2024, The Authors. (c) Computational modelling approach for constructing the NNP; (d) The reduction of Cu_2_O to Cu under different pH, potential, and SSAs, as well as the distribution of oxygen along the z axis for the last frame of OD‐Cu (SSA = 0.034 Å^−1^) with different oxygen concentrations; (e) The model of the Cu_2_O slab with different SSAs, and the configurations of OD‐Cu structures. Reproduced with permission [289]. Copyright 2024, Nature Publishing Group. (f) Workflow of expanded Grad‐CAM generation; (g) Expanded Grad‐CAM attention map for Ag and Ni−N/C. Reproduced with permission [45]. Copyright 2025, American Chemical Society.

Electric fields play a crucial role in regulating the electrocatalytic adsorption and conversion of CO_2_, yet they are difficult to quantify. To address this, Cui et al. [287] proposed using infrared/Raman spectral signals from CO_2_ molecules as descriptors, combined with machine learning, to quantify the influence of electric fields on catalytic reactions (Figure 27a). Using metal‐doped graphitic C_3_N_4_ (M@g‐C_3_N_4_) as the catalytic system, they constructed saturated cluster models for 27 different metal single‐atom catalysts. They analyzed CO_2_ adsorption configurations, adsorption energies, and charge transfer behaviors under electric fields of varying orientations and intensities, establishing a spectral‐property dataset comprising 972 data points. This ML model predicted adsorption energies and electric field strengths from spectral signals using a convolutional neural network (CNN). The attention mechanism revealed intrinsic correlations between spectral features and adsorption patterns, enabling reverse prediction of electric field strengths from spectra. Results indicated that Zr@g‐C_3_N_4_ exhibited the strongest CO_2_ adsorption free energy and charge transfer, with catalytic activity further enhanced under appropriate electric fields. This model not only enables digital interpretation of electric field effects but also provides a novel approach for monitoring and regulating electrocatalytic reaction environments at the molecular level.

The influence of pH on catalyst performance is crucial in CO_2_RR. For instance, Sn‐based catalysts generally exhibit high activity and selectivity toward HCOOH formation, yet significant variations are observed among different structural types of Sn, with the underlying mechanisms remaining unclear. Wang et al. [288] employed large‐scale data mining, ab initio computations, ML force field accelerated MD simulations, and pH‐field coupled modelling to unravel the adsorption differences of the key intermediate *OCHO on Sn catalysts with different structures at the microscopic level. They first conducted data mining and analysis of over 2300 experimental literature reports from the past decade, further emphasizing the advantages of Sn‐based catalysts in HCOOH production. They also discovered a general trend where HCOOH selectivity on Sn‐based catalysts increases with rising pH across different pH conditions. Subsequently, they systematically investigated the response of single‐atom tin (e.g., Sn−N−C) and polyatomic tin (e.g., SnO*_x_* and SnS*_x_*; *x *= 1, 2) to the adsorption behavior of the key intermediate *OCHO under varying pH conditions. Sn−N_4_−C and polyatomic Sn exhibited opposite directions of dipole moment changes during *OCHO formation, leading to entirely different trends in their response to electric fields. Sn−N_4_−C exhibited greater stability under alkaline conditions, whereas polyatomic Sn is weakened in such environments. Consequently, they displayed opposite pH dependencies in volcano plot evolution: the vertex of Sn−N_4_−C shifts to the right with increasing pH, while that of polyatomic Sn shifts to the left (Figure 27b). Further experimental verification of turnover frequency and current density results strongly corroborates theoretical predictions, confirming the model's reliability. This work demonstrates that single‐atom Sn enhances activity by strengthening *OCHO adsorption, whereas polyatomic Sn improves performance through moderate adsorption weakening, providing explicit theoretical guidance for differentiated catalyst design.

Catalyst surfaces undergo dynamic reconstruction during electrocatalytic processes, and the true surface state directly determines catalyst activity and selectivity. However, experimental characterization is often constrained by reoxidation and dynamic changes, leading to long‐standing debates regarding its true reaction structure and active sites. Lian et al. [289] constructed and validated a chemically accurate neural network potential (NNP) using DFT data (Cu, Cu_2_O, and intermediate Cu*_x_* O systems) as training sources, in combination with active learning methods. This NNP was employed to simulate the structural evolution of oxide‐derived copper (OD‐Cu) under electrochemical conditions (Figure 27c). By incorporating experimental conditions such as potential, pH, and specific surface area (SSA), the reduction process of OD‐Cu and oxygen distribution patterns were systematically revealed (Figure 27d). At low pH, OD‐Cu was reduced completely; at high pH, Cu_2_O was the most stable phase; under neutral conditions, partially reduced states exhibited the lowest energy. Applied potential was able to drive the reduction process, with –0.39 VSHE being the calculated limiting potential at which all reduction steps became exothermic reactions. Simultaneously, SSA significantly influenced the degree of reduction: smaller particle size (larger SSA) facilitated oxygen retention, with residual oxygen primarily enriching at the surface to form Cu_2_O (Figure 27e). When surface capacity was insufficient, oxygen accumulated in the bulk phase. Kinetics analysis revealed that oxygen diffusion from the bulk to the surface is quite slow, requiring timescales ranging from seconds to hours for complete reduction. Overall, this work systematically reveals the dynamic structural evolution of OD‐Cu during electrochemical reduction through high‐precision neural network potentials and large‐scale molecular dynamics simulations. It explains the thermodynamic and kinetic origins of residual oxygen and clarifies the nature of stable structures and active sites under different experimental conditions, providing theoretical foundations for understanding and regulating its unique performance in CO_2_RR.

Similarly, to address the high cost and complex mechanisms involved in monitoring catalyst degradation during electrocatalytic CO_2_ reduction reactions, Shin et al. [45] recently developed an end‐to‐end interpretable machine learning framework. This approach accurately predicts catalyst status and identifies degradation mechanisms within sub‐second time scales using simple LSV curves. They first collected 5196 LSV experimental data, covering different membranes, electrodes, and catalyst types, and trained a convolutional neural network (CNN) model based on these data to achieve high‐precision prediction of total current and Faraday efficiency. To enhance model interpretability, the expanded Grad‐CAM attention map was generated by integrating the Grad‐CAM method from explainable artificial intelligence (XAI) technology (Figure 27f). By overlaying Grad‐CAM importance scores across each LSV curve, a panoramic view of critical regions emerged, revealing the dynamic impact of different voltage zones on catalyst performance during degradation. Taking the Ag catalyst as an example, high‐importance regions predominantly clustered in the negative voltage zone around −3.2 V, indicating external factors like bicarbonate deposition and pH shifts as primary degradation drivers. In contrast, attention for the Ni–N/C catalyst progressively shifted toward the kinetic region within the negative voltage zone, indicating degradation primarily stemmed from active site loss, such as Ni single atoms agglomerating into nanoparticles, leading to reduced catalytic activity (Figure 27g). Furthermore, they validated the reliability of XAI interpretations through multiple characterization techniques, confirming that predicted key degradation mechanisms aligned with actual physicochemical changes. By establishing an end‐to‐end analytical framework that integrates CNN prediction with XAI interpretation using LSV curves as a low‐cost universal descriptor, this work enables real‐time prediction of catalyst performance and systematic analysis of degradation mechanisms. This work provides an effective tool for catalyst design, optimization of operating conditions, and online monitoring, while significantly reducing reliance on costly experiments.

It is also worth noting that, as discussed in Section 4, external fields (light, heat, magnetic fields, and pressure) have attracted increasing interest for regulating the microenvironment in eCO_2_RR. These external stimuli can modify interfacial electronic structures, alter local electric‐field distributions, induce solvent reorganization, and even trigger catalyst surface reconstruction, thereby influencing reaction pathways and product selectivity. However, the effects of external fields typically involve multi‐scale coupled processes, making experimental exploration highly labor‐intensive and associated with a large parameter space. Conventional theoretical tools (e.g., DFT or MD) also face inherent limitations in simulating phenomena such as photo‐excited carrier dynamics, spin‐magnetic field interactions, or pressure‐driven structural evolution, thus falling short of capturing the full complexity of field‐dependent interfacial behavior.

Under this background, artificial intelligence presents new opportunities for predicting field‐assisted microenvironment regulation. Through learning multi‐modal datasets that contain external‐field variables (e.g., operando spectra, electrochemical responses, structural evolution trajectories), AI models can construct data‐driven relationships linking external field inputs, interfacial responses, and catalytic outcomes. In particular, GNNs, NNPs, and physics‐informed deep learning enable efficient large‐scale approximations of many‐body effects, including photo‐induced dynamics, thermally driven structural relaxation, and magnetic‐field‐dependent electron spin behavior. Moreover, active learning and Bayesian optimization frameworks can autonomously identify optimal external‐field conditions within a vast parameter space, thereby reducing experimental trial‐and‐error and enabling a predictive‐first strategy for field regulation. We believe AI‐driven prediction of external‐field effects is not only a useful supplement to traditional theoretical methods but also an important step toward overcoming current barriers in understanding complex CO_2_RR microenvironments. Since external fields reshape the interfacial energy landscape and reaction channels, AI offers a powerful means to uncover the hidden couplings embedded in high‐dimensional data, helping shift external‐field research from empirical exploration toward a more designable and predictive paradigm. As more datasets integrating operando spectroscopy, electrochemical measurements, and field‐response information become available, AI is expected to further clarify causal mechanisms and promote more precise and controllable microenvironment engineering in CO_2_RR.

Overall, by integrating AI with DFT/MD, AI has not only improved computational efficiency but also enabled functions such as high‐throughput screening, prediction of complex reaction pathways, and modelling of dynamic micro‐reaction networks. These advancements have enabled researchers to establish a closer connection between the atomic scale and the system scale, providing new theoretical tools and insights for designing efficient and stable electrocatalysts. However, current AI models still have limitations in terms of data dependency, interpretability, and cross‐system generalization capabilities, particularly in accurately describing complex interface reactions (such as electrode‐electrolyte interfaces). Future developments should focus on constructing high‐quality, multi‐scale theoretical and experimental datasets to enhance the reliability of AI predictions. Additionally, exploring the deep integration of AI with DFT/MD is essential to achieve precise simulations of reaction kinetics, catalyst structural evolution, and microenvironmental effects under operational conditions. Through these efforts, AI is expected to become a vital tool for driving the design of efficient CO_2_RR catalysts, optimizing reaction selectivity, and understanding complex interfacial mechanisms, opening a new chapter in the study of electrochemical CO_2_ conversion.

## 6 Perspectives and Suggestions

### 6.1 From Laboratory to Industrialization Process

**FIGURE 28:**
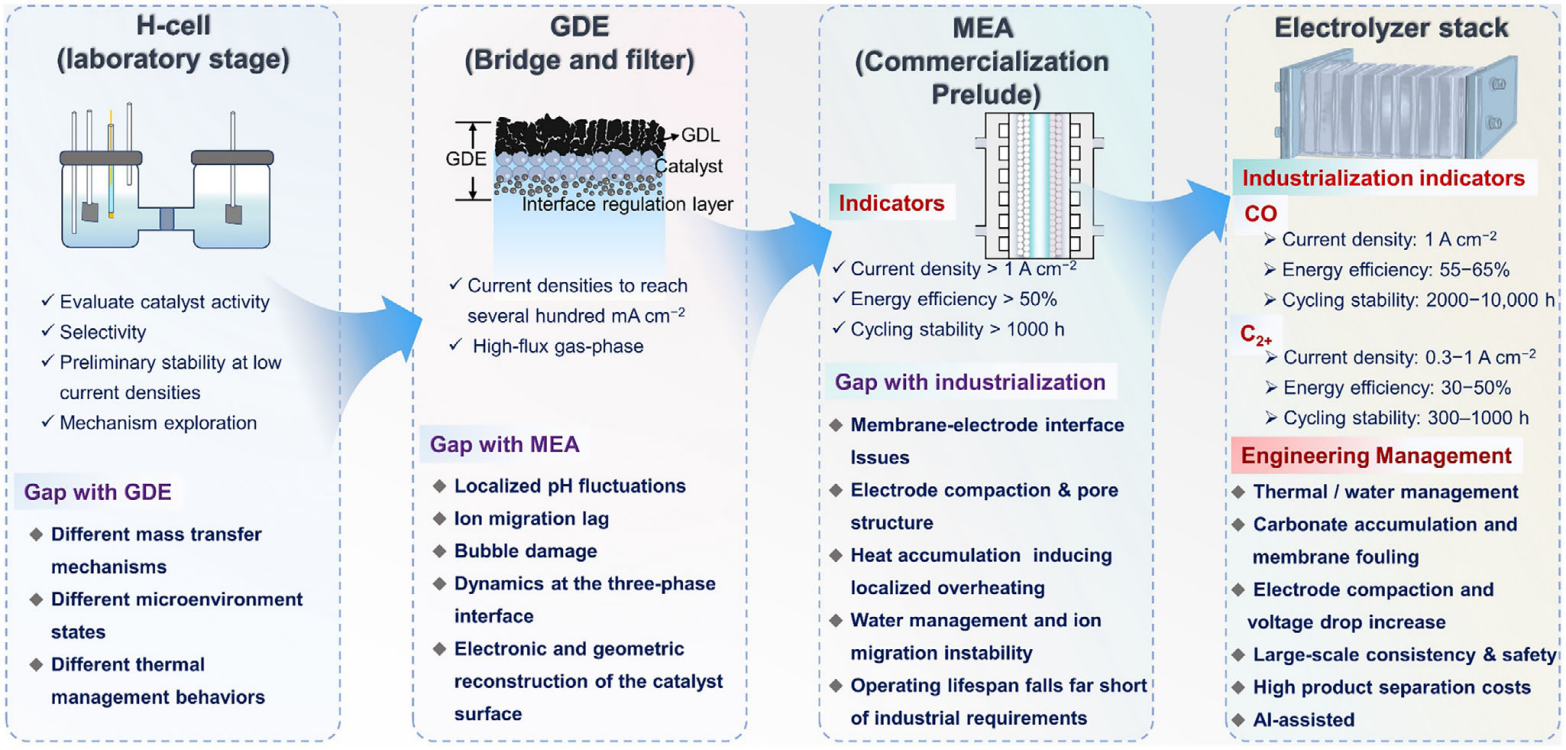
Comparison of industrialization processes.

The progress of eCO_2_RR from laboratory fundamental research to industrialization is still in a critical development stage. Despite remarkable progress in catalyst design, interfacial regulation, and mechanism analysis, these achievements are difficult to directly translate into practical applications. This is because the transition from micro‐ to macro‐scale systems not only alters the operating conditions but also shifts and complicates the dominant factors controlling the reaction. In this process, H‐cells, GDEs, and MEAs play distinct roles in fundamental mechanistic studies, high‐current‐density validation, and exploration of industrial‐scale pathways, respectively (Figure 28). But the differences among them also constitute the challenge that prevents laboratory catalyst performance from naturally extending to industrial settings.

In the laboratory stage, H‐cell systems serve as the most commonly used research platform. Due to their simple operation, controllable data, and ease of catalyst characterization, H‐cells are primarily used to evaluate catalyst activity, selectivity, and preliminary stability at low current densities (<100 mA cm^−2^). It also provides a foundation for analyzing intermediate evolution, surface reconstruction, and C−C coupling pathways. Nevertheless, the overall environment in H‐cells differs significantly from that in industrial systems, making it difficult to directly extrapolate conclusions. The main points are as follows: (i) Different mass transfer mechanisms. H‐cells rely on dissolved CO_2_, which is limited by solubility and diffusion rates, whereas industrial systems employ direct gas‐phase supply, resulting in completely different CO_2_ activities and concentration gradients. (ii) Different microenvironment states. pH changes slowly in H‐cells and are buffered by the solution, while in GDEs or MEAs, local pH can rise sharply within milliseconds and form strong gradients, altering intermediate stability and the dominant mechanistic steps. (iii) Different thermal management behaviors. H‐cells are approximately isothermal, whereas in high‐current‐density systems, Joule heating and reaction exothermicity accumulate nonlinearly, causing significant increases in interfacial temperature that affect CO_2_ solubility, adsorption energies, and catalyst reconstruction rates. These deviations collectively lead to phenomena observed in H‐cells (e.g., stable oxidation states of Cu‐based catalysts or high C_2+_ selectivity) that no longer persist under industrial conditions, and may even undergo mechanism shifts. Relying solely on H‐cell data for catalyst evaluation is vulnerable to failures in translating laboratory findings to industrial applications. To advance catalysts from mechanistic studies to engineering applications, it is essential to re‐evaluate the effects of these factors on reaction pathways and stability.

GDE serves as the first screening platform bridging laboratory mechanistic studies and industrial conditions. The high‐flux gas‐phase supply significantly promotes CO_2_ availability, allowing current densities to reach several hundred mA cm^−2^. While this addresses the limitations of H‐cells, it also shifts the interfacial environment from the relatively steady‐state conditions to a highly dynamic, non‐steady‐state regime: (i) local pH within the catalyst layer rises sharply over micrometer scales; (ii) ion transport lags considerably behind the reaction rate, leading to uneven potential distribution at the membrane interface; (iii) bubble nucleation and detachment induce transient resistance fluctuations; (iv) fluctuations in electrode water content continuously change the structure of the three‐phase interface; (v) the catalyst surface undergoes ongoing electronic and geometric reconstruction under strong overpotentials and high flux. These dynamic effects change the steady‐state concentration of intermediates, the probability of C−C coupling, and the direction of competing reaction pathways. Consequently, numerous catalysts exhibiting outstanding performance in H‐cells experience reduced selectivity, intensified restructuring, or heightened side reactions under GDE conditions. Common issues such as electrode drying, salt precipitation, and carbonate accumulation further narrow the effective operating window, resulting in a highly time‐dependent evolution of structure–performance relationships. Therefore, GDEs are not only used to verify actual reaction pathways at industrial current densities but also to assess whether catalysts possess transferability. Current studies indicate that even Cu‐Al alloys or multimetallic catalysts struggle to simultaneously achieve high activity, high selectivity, and long‐term stability in GDEs, highlighting the much greater complexity of industrial conditions compared to laboratory models.

At MEA stage, system performance is determined by the interplay among the catalyst layer, the proton or anion exchange membrane, and the flow field structure, shifting the research focus from enhancing mass transport to overall system coordination. Successful MEA operation requires three industrial benchmarks: high current density (>1 A cm^−2^), high energy efficiency (>50%), and long‐term cycling stability (>1000 h). However, most current studies remain limited to operation times under 200 h, and achieving high energy efficiency, selectivity, and stability simultaneously remains challenging. In addition to the issues observed in GDE systems, MEAs introduce new engineering challenges: chemical stability at the membrane‐electrode interface; pore structure changes caused by electrode compaction; dynamic control of local water content; and accumulation of byproducts (e.g., OH^−^, ${\mathrm{CO}}_{2}^{-}$) at the membrane‐electrode interface, leading to contamination. Although zero‐gap MEA designs are advantageous for modular scale‐up and high‐efficiency operation, they also introduce new challenges that become critical constraints in the industrialization process.

During scale‐up, as the operating current density increases, Joule heating and reaction heat accumulate within the catalytic layer. It causes local temperatures to rise by several tens of degrees Celsius in less than one second, even reaching as high as 40−80°C. The temperature increase not only reduces CO_2_ solubility, but also alters the adsorption/desorption rates of intermediates, the transport of protons and OH^−^, and the stability of active sites, which influences C_2+_ selectivity and accelerates the degradation of membranes and catalyst layers. Both theoretical and experimental studies have shown that moderately elevated operating temperatures can improve mass transfer and ionic conductivity. However, without effective thermal management, localized overheating may induce performance fluctuations and shorten device lifetime. To quantify these thermal effects, researchers have employed embedded micro‐thermocouples, infrared thermography, operando Raman peak shifts, or fluorescence‐lifetime imaging, and fiber Bragg grating (FBG) sensors to obtain spatial temperature distribution within the catalyst layer. Combined with current distribution, interfacial spectroscopic data, and product analysis, these temperature measurements enable the construction of coupled thermal, mass‐transport, and electrochemical models, providing quantitative guidance for industrial MEA design and cooling strategies, while also offering reliable experimental support for AI‐driven optimization and prediction.

In the multi‐scale scale‐up from H‐cells to GDEs and MEAs, artificial intelligence (AI) serves as a bridge connecting fundamental mechanistic studies with engineering applications. Because H‐cells, GDEs, and MEAs operate under different boundary conditions and dominant physical parameters, traditional empirical approaches are often insufficient for achieving transferable performance. AI can integrate multi‐scale data, apply transfer learning, and identify high‐dimensional reaction descriptors to extract intrinsic features that remain consistent across platforms, such as adsorption energy gradients, local solvation patterns, and ion transport resistances. At the engineering level, AI can enable closed‐loop process control based on temperature, pressure, flow rate, and online product signals, dynamically adjusting CO_2_ flow, humidity, or pulsed potentials to maintain interface stability. At the system design level, digital twins combining three‐dimensional multiphysics simulations with experimental data can predict pressure drops, hotspot formation, and cross‐membrane contamination during stack operation. From an economic perspective, AI can couple electrode geometry and operating windows with Techno‐Economic Analysis/Life Cycle Assessment (TEA/LCA) models to optimize yield, energy efficiency, and cost simultaneously. Furthermore, NNPs and GNNs facilitate cross‐scale predictions from catalyst surface structures to reactor parameters, establishing a unified data framework that links materials screening, interface design, and system engineering.

From the perspective of economic and technical feasibility, the transition from H‐cell to GDE/MEA involves not only scaling up catalyst performance but also balancing system energy efficiency, operational stability, and overall cost. Operating at high current densities helps increase single‐pass productivity, but it may also introduce mass transfer limitations, heat accumulation, and accelerated membrane degradation, ultimately reducing system efficiency. Achieving a balance among high productivity, high energy efficiency, and long‐term durability has become an indispensable consideration in industrial design. This balance relies on cross‐scale modeling, AI‐assisted optimization, and experimentally validated strategies. Industrial assessments should go beyond Faradaic efficiency or current density alone, incorporating energy consumption per unit of product, product separation costs, catalyst lifetime, and broader lifecycle carbon and energy metrics (TEA/LCA). AI can package experimental parameters, reactor geometry, thermal management strategies, and economic models to perform multi‐objective trade‐offs. For example, it is possible to maximize net system energy efficiency and minimize operational costs while maintaining high C_2+_ selectivity. This interdisciplinary assessment will reshape the prioritization of early‐stage catalyst screening, aligning it more closely with industrial feasibility.

In general, the industrialization of eCO_2_RR is not a simple matter of scaling up an electrochemical reaction, but rather a system‐level challenge that spans interface science, reaction engineering, thermal management, membrane technology, and intelligent control. H‐cells offer fundamental mechanistic insights, GDEs reveal how high‐flux interfaces behave in practice, and MEA systems serve as the engineering platform for developing viable industrial routes. As experimental methodologies, in situ/operando characterization, thermal‐management approaches, and data‐driven frameworks continue to advance, the transition from laboratory studies to industrial operation will increasingly shift from empirical trial‐and‐error toward a combination of mechanism‐based understanding and data‐guided optimization. This comprehensive strategy is expected to accelerate the establishment of a practical industrial foundation for eCO_2_RR and support the development of a sustainable carbon‐cycle technology in the future.

### 6.2 Suggestions

With the rapid advancement of CO_2_RR electrocatalysis in catalyst development, interfacial regulation, and reaction system engineering, the research focus has gradually shifted from the initial question of “feasibility” to “how to achieve high efficiency, stability, and controllability”, and further to “realizing system‐level integration and transition to industrialization”. The challenges we face have also evolved from single‐property optimization to a systematic understanding and precise control of multi‐scale, multi‐physics, and dynamic interfacial behavior. However, reflecting on the past decade's progress reveals that the true constraints on further breakthroughs in CO_2_RR lie not only in the pace of material development but also in the depth of understanding of dynamic, complex reaction systems and the lack of interdisciplinary integration capabilities. The key to the future lies in transcending traditional steady‐state thinking to embrace a comprehensive framework integrating dynamic microenvironments, external field interventions, in situ observability, and intelligent regulation. This will enable CO_2_RR to evolve into programmable, scalable energy chemical systems.

At the level of performance balancing, the greatest scientific challenge in advancing CO_2_RR lies not in prioritizing reactivity, selectivity, or stability individually, but in resolving the inextricable trade‐offs between these three properties. High reactivity often relies on high‐energy sites or metastable surfaces, yet such sites are prone to reconstruction or deactivation during prolonged operation, compromising stability. High selectivity necessitates precise control over intermediates, often at the expense of reaction rate or operational window narrowing; while common stability enhancement methods (e.g., constructing inert structures or passivation layers) may directly weaken activity or limit intermediate conversion. In other words, these three properties are not merely parallel metrics but represent an inherently tension‐ridden, mutually exclusive systemic challenge. Future breakthroughs hinge on developing dynamically balanced systems where catalysts continuously self‐regulate between activity, selectivity, and stability during operation. For instance, maintaining sites in a highly active yet non‐oversaturated state via pulsed electric fields or multi‐field coupling, or achieving dynamic activity within steady states through flexible frameworks and adaptive interface designs, could establish new equilibrium points amidst these contradictions.

At the microscopic level, CO_2_RR is not a stable, homogeneous surface reaction but a complex system involving multiscale dynamic coordination, including charge transfer, mass supply, solvent rearrangement, and ion migration. Most existing studies rely on characterization under steady‐state conditions or idealized simulations, overlooking the intense non‐equilibrium characteristics inherent in real electrolytic environments. For instance, transient fluctuations in local pH, non‐uniform cation distribution, and dynamic reorientation of water molecules can influence intermediate formation and transformation across nanosecond to micrometer timescales. This dynamism determines that catalysts do not operate in isolation but are embedded within an evolving microenvironment. Consequently, future breakthroughs require shifting from steady‐state catalysis to dynamic regulation, constructing interfacial systems capable of adaptation, self‐feedback, and ultimately self‐healing.

Regarding product orientation, the highly selective synthesis of C_2+_ compounds remains an insurmountable bottleneck. Although Cu‐based catalysts exhibit unique advantages, the diversity of their product pathways reveals that the delicate balance of “coupling–protonation–deprotonation” remains elusive. The core challenge lies in precisely trapping *CO aggregation and coupling at metastable interfaces while preventing rapid hydrogenation toward methane and other side pathways. This challenge extends beyond catalyst site design to encompass the harmonization of electric field distribution, localized electrolyte environments, and dynamic electronic states. In other words, future C_2+_ regulation must be grounded in a systematic design principle involving the tripartite synergy of multifunctional sites, dynamic field control, and interfacial microenvironments, rather than relying solely on optimizing single‐metal structures.

Characterization serves as an essential means to address the invisible challenges required for achieving these breakthroughs. Existing operando techniques often remain confined to single signals and single scales, struggling to reconstruct the complete dynamic landscape of the interface. Future characterization should shift toward multimodal, correlated in situ platforms. These platforms must simultaneously track intermediate configurations, valence state evolution, ion distribution, and interface morphology within the same reaction environment. Data consistency is achieved through time‐stamp alignment and spatial correlation. Only then can characterization evolve from passive recording to active sensing, transforming CO_2_RR from post‐reaction analysis into an in‐process controllable system.

In terms of engineering scale‐up and long‐term operation, challenges become more tangible and complex. Electrode and reactor designs optimized for laboratory scales often maintain ideal performance under meticulous conditions. However, when electrode areas expand by a factor of hundreds or thousands, mass transfer limitations, heat accumulation, bubble entrapment, and amplified side reactions rapidly destabilize performance. More critically, active sites frequently form on fragile structures like defects, boundaries, or metastable crystal planes. Long‐term operation inevitably induces restructuring and poisoning, leading to performance degradation. The solution may not lie in blindly pursuing absolutely stable materials, but rather in introducing catalyst and electrode systems capable of dynamically adjusting their recovery abilities in response to reaction changes. This could involve utilizing pulsed electric fields, oscillating potentials, or external field interference to achieve in situ repair or regeneration, or designing catalytic materials with flexible structures and adaptive regulation capabilities.

Theoretical and computational approaches also face a turning point. Traditional DFT models predicting adsorption energies on idealized vacuum surfaces can no longer adequately describe the complexity at the triple interface of real electrolytes, electrodes, and electric fields. Future approaches must embrace multiscale multiphysics modelling, including explicit solvent dynamics, ion migration modelling, and AI‐driven high‐throughput prediction. Crucially, theory and experiment should transcend mutual validation, instead forming a cyclical mechanism of experimental inversion and theoretical correction to dynamically reconstruct real systems and uncover underlying mechanisms.

Concurrently, external field assistance and AI integration open new avenues for this field. External fields serve not merely as energy supplements but as additional degrees of freedom to overcome selectivity bottlenecks under conventional electric fields. AI may emerge as the core engine for achieving truly end‐to‐end self‐optimizing platforms, transforming CO_2_RR from passive experimentation into an intelligently regulated process. This trend signals that future CO_2_RR will transcend being merely a convergence point of materials science and electrochemistry, evolving into a frontier domain deeply integrated with data science, information science, and systems engineering.

In summary, the future of electrocatalytic CO_2_ reduction is no longer about single‐dimensional breakthroughs but represents a quintessential cross‐scale, cross‐disciplinary, and cross‐system integrated challenge. Particularly, the coupled trade‐offs between activity, selectivity, and stability necessitate moving beyond linear optimization approaches toward dynamic equilibrium and adaptive regulation. From electrons to interfaces, from microenvironments to reactors, from experimental characterization to digital twins, every component must be integrated into holistic, systemic thinking. Only then can CO_2_RR truly emerge from the laboratory to become a pivotal hub technology underpinning the carbon circular economy and green energy transition.

## Conflicts of Interest

The authors declare no conflicts of interest.
